# Energy transfer leaves fingerprints in cyanine photoswitching behavior

**DOI:** 10.1371/journal.pcbi.1014322

**Published:** 2026-05-18

**Authors:** Vincent Ebert, Markus Sauer, Sören Doose

**Affiliations:** 1 Department of Biotechnology and Biophysics, Biocenter, Julius-Maximilians University, Würzburg, Germany; 2 Rudolf Virchow Center, Research Center for Integrative and Translational Bioimaging, Julius-Maximilians University, Würzburg, Germany; University of Toronto Mississauga, CANADA

## Abstract

Super-resolution microscopy resolves molecular structures in biological systems but is limited by properties of the fluorescent labels. dSTORM experiments with multiple Cy5 fluorophores in sub-10 nm spaced labelling positions have shown distinct cumulative localization counts over time, depending on the distances between the fluorophores, that suggest Förster resonance energy transfer between ON states and long living OFF states. This photophysical effect hinders precise localization of the individual emitters but produces a photoswitching fingerprint that can be used to draw conclusions about sub-10 nm spatial conformations in molecular structures that are not spatially resolvable. Here we present a theoretical framework for analysing the photophysical systems that yield distinct fingerprint signatures. We developed a Python-based continuous-time Markov chain simulation software package that reproduces the photophysical processes of organic fluorophores. We show that the established photophysical models of Cy5 extended by Förster resonance energy transfer between the excited singlet state and the long living OFF state explain experimental fingerprint signatures as seen in dSTORM experiments. Matching experimental signatures including fluorescence lifetimes provides evidence for an additional energy transfer to the radical anion of cyanine dyes like Cy5. This work contributes to the understanding of proximity-based photophysical processes and paves the way for future development of sub-10 nm dSTORM imaging.

## Author summary

The spatial resolution in fluorescence microscopy is physically limited at a few hundred nanometres. State-of-the-art super-resolution microscopy techniques provide means to resolve smaller molecular structures at the nanometre length scale. However, superior image resolution requires dense labelling schemes such that fluorescent labels are often spaced at distances that allow for non-radiative energy transfer. In single-molecule localization microscopy (SMLM), a super-resolution technique that builds on stochastic activation of single-molecules for precise spatial localization, Förster resonance energy transfer can report on sub-10 nm distances and influence single-molecule fluorescence blinking. The notion that Förster resonance energy transfer between the excited singlet state of organic fluorophores and long living OFF states that are required for SMLM, can explain altered blinking dynamics is fundamental for understanding experimental observations and improving SMLM imaging capabilities. Here we present a photophysical model for the organic fluorophore Cy5 in different conditions including those for SMLM to quantify sub-10 nm interactions. Precise knowledge about the photophysical model with proximity-based photophysical processes is used to gain information about the local densities of fluorescent labels in sub-10 nm SMLM imaging and thus improve SMLM imaging at the molecular scale.

## Introduction

Resolution in optical super-resolution microscopy now reaches molecular length scales under favorable conditions. Single-molecule localization microscopy (SMLM) is at the forefront in terms of resolution, but relies on stochastic observation of single fluorophores, which is limited by sample condition, multiplexing, and acquisition time [1]. The SMLM technique ‘direct stochastic optical reconstruction microscopy’ (dSTORM) is based on a stochastic ON/OFF switching mechanism (also known as blinking) that results in small subpopulations of fluorophores in their ON state and hence in separable point spread functions (PSF) in a wide field microscope [2]. Depending on label density, localization precision and localization accuracy, spatial resolutions of around 20 nm are commonly achieved. Many other super-resolution microscopy techniques, including variants of PAINT, STORM, PALM and MINFLUX, are constantly being developed to improve the resolution limits to a few nanometers [3–5]. However, achieving a resolution in the sub-10 nm domain remains challenging because it often requires advanced experimental procedures and equipment; and because the required label density results in inter-fluorophore distances that might allow for energy transfer or electronic interactions between fluorophores.

In dSTORM, sample preparation, choice of fluorophores, measurement, and subsequent analysis are well established. The work of Helmerich et al. [6] showed that Cy5 molecules with inter-fluorophore distances of 3 – 6 nm exhibit an altered blinking behavior in dSTORM. It is characterized by an early, steep increase in the empirical cumulative distribution function (eCDF) for localizations (see their Fig 1g). We call the quantitative analysis of the localization/ photon arrival times eCDF by photoswitching fingerprint analysis (PFA). The effect observed in such PFA on DNA-origami [6] and in a protein environment [7] (PicoRuler) increases as the distance between the fluorophores decreases down to 3 nm, suggesting an energy transfer-like mechanism as the cause. Distance-dependent effects were also seen through shortened fluorescence lifetimes and shorter OFF times at the beginning of time-resolved fluorescence lifetime (FLIM) measurements (see Fig 2 in Helmerich et al. [6]).

**Fig 1:**
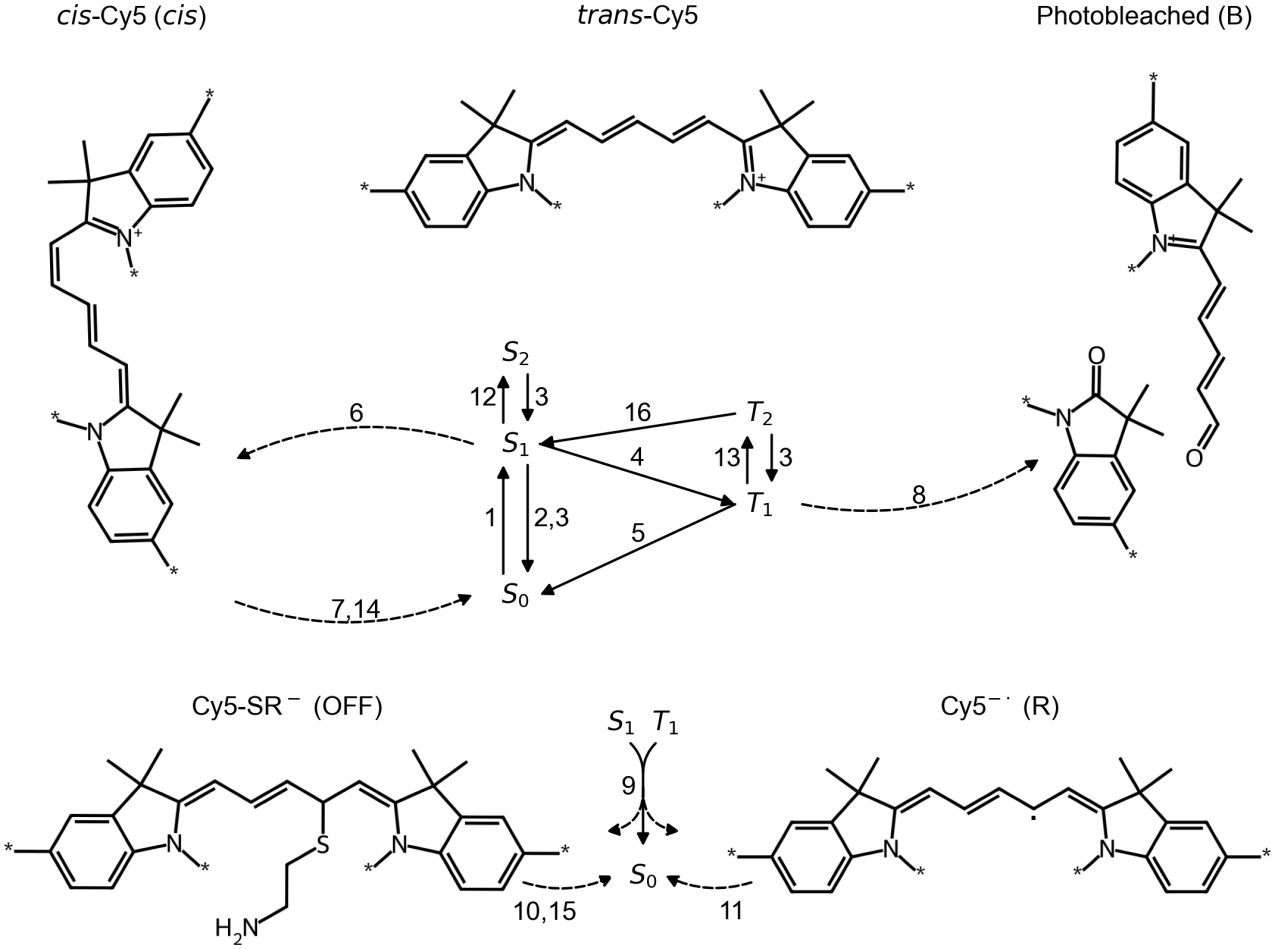
Photophysical model of Cy5 with transitions 1 to 16. General photophysical processes: 1 – photoexcitation (EXC), 2 – fluorescence (FLU), 3 – internal conversion (IC) + vibrational relaxation (VR), 4 – intersystem crossing (ISC) + VR, 5 – ISC + VR, 6 – isomerization (ISO), 7 – back-isomerization (BISO), 8 – photobleaching (BLE); dSTORM-specific photophysical processes: 9 – photoinduced electron transfer from thiol (PET) to either S_1_ or T_1_ and ending up in either Cy5-SR^-^, Cy5^-∙^ or S_0_, 10 – OFF to ON via thermal elimination (TE) or photoinduced uncaging (PU), 11 – oxidation (OXI); Energy transfers (only acceptors vary, donor transitions always from S_1_ to S_0_): 12 – singlet-singlet annihilation (SSA), 13 – singlet-triplet annihilation (STA), 14 – FRET to *cis*-Cy5 (CET), 15 – FRET to OFF (OET), 16 – reverse ISC (RISC). Note the specific implementations described in the text.

**Fig 2:**
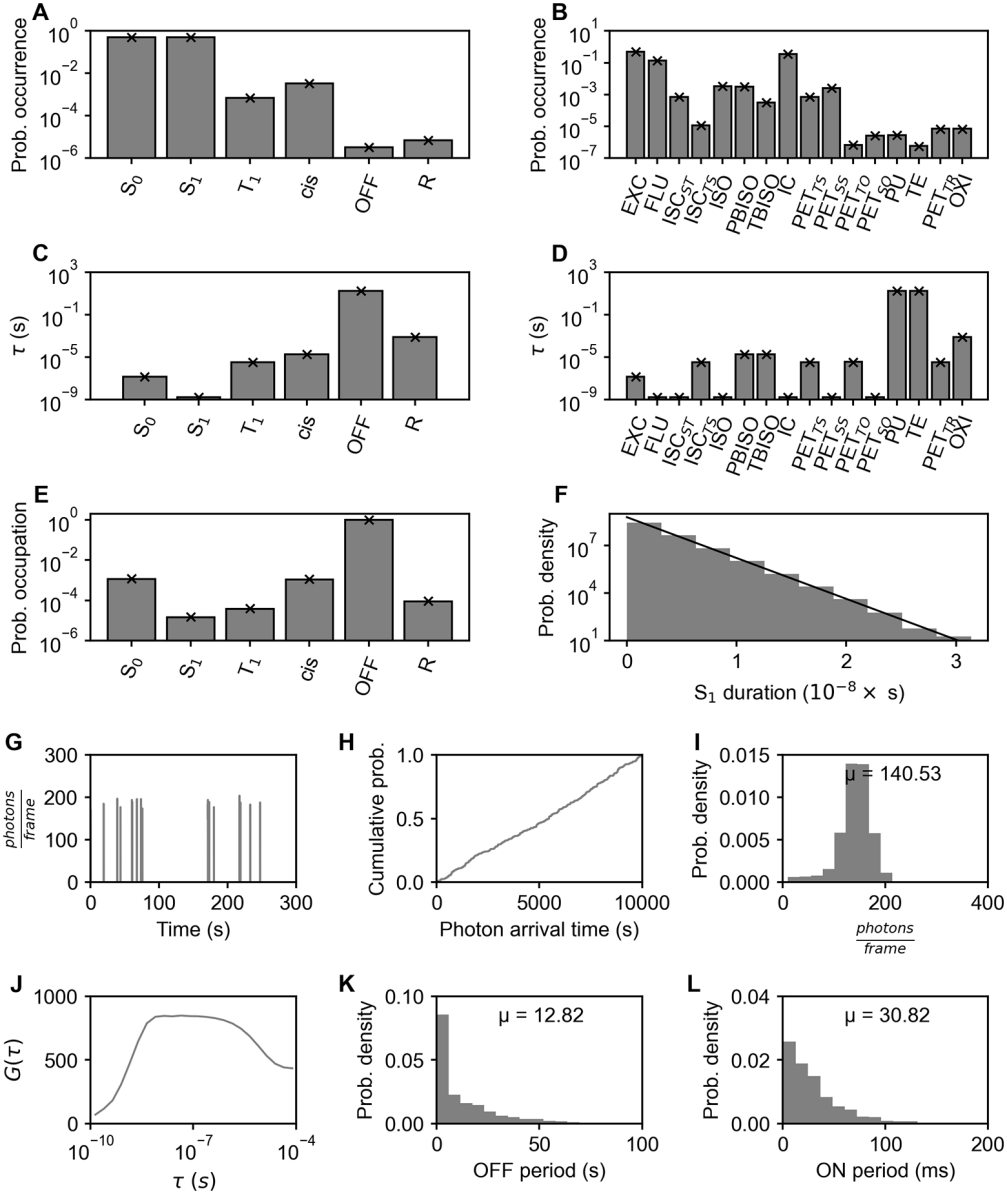
Photophysical statistics of a single Cy5 fluorophore with continuous excitation. Photobleaching is disabled. **(A – F)** Statistics that are experimentally not available. They can also be predicted (crosses for A – E, line for F) using tools like the limiting distribution of the Markov chain. **(A)** The probability of occurrence of each photophysical state. **(B)** The probability of occurrence of each photophysical transition. **(C)** The lifetime of each photophysical state. **(D)** The transition lifetime of each photophysical transition. **(E)** The probability of occupation of each photophysical state. **(F)** The ePDF of durations of the photophysical state S_1_ overlaid with an exponential decay of corresponding relaxation time. **(G – L)** Analyses that are experimentally available. **(G)** The fluorescence trajectory showing photons per frame over time. **(H)** The eCDF of photon arrival times. **(I)** The ePDF of photon counts per frame. **(J)** The fluorescence correlation curve G(τ). **(K)** The ePDF of OFF periods (consecutive frames with photon count < 10). **(L)** The ePDF of ON periods (consecutive frames with photon count ≥ 10).

Cy5 is a well-known fluorophore belonging to the class of cyanine dyes that is frequently used in dSTORM experiments due to its strong absorption, favourable photon output, good photostability, spectral properties that differ from cellular autofluorescence, and its ability to photoswitch in respective buffer [8]. As a cyanine dye, photophysical models of Cy5 introduce many photophysically accessible and energetically different states [9,10], which are further extended in dSTORM by thiol adduct and radical-state formation. Such a complex system of photophysical states and possible transition pathways, including a photochemical reaction with thiols, is well described by Gidi et al. [11] and earlier studies [12].

The aim of this work is to derive a rigorous theoretical explanation that can explain experimental data, particularly the PFA signatures, based on fundamental photophysical principles. To test different hypothetical photophysical models, we developed a simulation machinery based on a continuous-time Markov chain simulation with the stochastic simulation (Gillespie) algorithm [13]. This allows us to test systems of up to four fluorophores in close proximity, with multiple FRET pathways possible. We show that a specific photophysical model for Cy5 that has been largely established qualitatively and quantitatively by other groups [9,11,14,15] and extended with FRET between the first excited singlet state as donor and the dSTORM specific OFF state as acceptor, leads to simulated PFA that agree well with experimental PFA [6].

Close comparison between simulated and experimental fluorescence lifetimes revealed a discrepancy that could not be resolved without introducing another so far not considered FRET pathway. We show that the dSTORM-specific radical anion [16–18] as acceptor is an exclusive candidate to explain the observed fluorescence lifetimes without compromising any other agreement between simulation and experiments.

## Theory

To simulate fluorescence emission patterns resembling those observed in experiments, we set up a photophysical model of Cy5 based on the following processes (Fig 1). Cy5 can be photoexcited (EXC) (transition 1) from its ground state S_0_ to its first excited singlet state S_1_ (1.97 eV) upon irradiation with visual light of around 640 nm. From there, it can undergo radiative deexcitation, i.e., fluorescence (FLU) (2), emitting a photon of around 1.84 eV, or it can undergo non-radiative deexcitation via internal conversion (IC) followed by vibrational relaxation (VR) (3), ending up back in S_0_. Alternatively, S_1_ can undergo intersystem crossing (ISC) followed by VR (4) ending up in the first excited triplet state T_1_, corresponding to around 1.34 eV [19]. ISC and VR (5) let Cy5 return to S_0_. Here, transitions among rotational and vibrational states by VR are not considered separately since their decay occurs ~1000-fold faster than electronic transitions. And since reverse ISC (RISC, delayed fluorescence) from T_1_ to S_1_ is very inefficient it is not further considered [20].

Cy5 is a red-absorbing cyanine dye consisting of a conjugated system with a polymethine chain and therefore exhibits isomerization processes between isoforms. Here, the exact photophysical pathways [9] are simplified to the isomerization (ISO) (*trans*-S_1_-to-*cis* transition) (6) and back-isomerization (BISO) (*cis*-to-*trans*-S_0_ transition) (7). Both ISO and BISO are thermally and photoinducible. However, at 640 nm, only the *trans* isomer absorbs significantly [11], so that the thermal component of ISO can be neglected. Additionally, radiative deexcitation of *cis* is assumed to be negligible [9,21,22]. Note that in our model, the internal conversion rate of *trans* S_1_ to S_0_ also includes contributions of twisted state relaxations that end up in *trans* again.

Photobleaching (BLE) is the irreversible photoinduced conversion of a dye into a non-fluorescent compound. This is most commonly initiated by a reaction with molecular oxygen (that has a non-reactive triplet ground state ^3^O_2_), activated either via photoinduced electron transfer or via energy transfer [23]. Hypothetically, photobleaching can affect any excited state; however, in this study, we condense photobleaching pathways into a single photobleaching transition that is implemented as the conversion of the first excited triplet state [24] T_1_ into a photobleached state B (8).

Cy5 in dSTORM imaging conditions populates non-radiative Cy5-SR^-^ (OFF) via thiol-mediated photoinduced electron transfer (PET) (9) either onto T_1_ followed by thiyl radical-assisted ISC and geminate radical combination (GRC) or onto S_1_ followed by GRC [11]. With a probability of 99.9%, the intermediate geminate radical pair in its singlet state undergoes geminate recombination (i.e., back electron transfer), repopulating the ground state directly [25]. The OFF state can undergo thermal elimination (TE) (10) to repopulate native Cy5 in its ground state. It can also be excited by various wavelengths promoting the conversion to Cy5 S_0_ via photoinduced uncaging (PU) [11,26] (10). If radical escape (RE) outcompetes thiyl radical-assisted ISC, the radical anion Cy5^-∙^ (R) is formed, which returns to S_0_ via oxidation (OXI) (11). Unless explicitly stated, we do not take the radical cation Cy5^+∙^ into account, as it requires special experimental conditions to be formed in significant yields [11,27]. We also do not consider an additional, chemically distinct OFF state, where the thiol group sits at a different C of the polymethine chain, as its contribution to the total OFF state population was shown to be either less than 10% or its lifetime to be on the same order of magnitude [11]. Note that whenever we refer to the ON state, we are summarizing all other photophysical states that are not the OFF state. This is because even though other states may be considered ‘dark’, their lifetimes are still short compared to that of Cy5-SR^-^ under standard dSTORM conditions. In addition to ON and OFF states of individual fluorophores, we also define ON and OFF periods. These are consecutive time bins (frames) where each frame’s photon count does or does not exceed a certain threshold. Note that a frame can hypothetically be considered OFF even if one or more fluorophores are in the ON state, but the emitted number of photons does not cross the detection threshold (Fig D in S1 Text). Factors that influence the probability to observe an ON frame include, but are not limited to, the number of fluorophores, the lifetimes and entering probabilities of non-emitting photophysical states, the irradiance, the probability of fluorescence per S_1_, the photon collection probability, the duration of a frame and the photon count threshold.

If multiple Cy5 molecules are in proximity of less than ~10 nm to each other, FRET influences their photophysical behaviour significantly. Singlet-singlet annihilation [28,29] (SSA) (12) transfers the energy of the donor S_1_ onto the acceptor S_1_, thereby exciting the acceptor to the second excited singlet state S_2_. IC and VR quickly return it to S_1_. Homo-FRET [30] transfers the energy of the donor S_1_ onto the acceptor S_0_, exciting it to S_1_. This energy transfer is not considered here, because it doesn’t influence any properties probed in our simulations and corresponding experiments. Singlet-triplet annihilation [28,29] (STA) (13) transfers the energy of the donor S_1_ onto the acceptor T_1_, exciting it to the second excited triplet state T_2_. Spectral overlaps indicate FRET of *trans*-Cy5 S_1_ onto *cis*-Cy5 [11] S_0_ (CET) (14), exciting *cis*-Cy5 S_0_ to *cis*-Cy5 S_1_. Finally, the proposed FRET of donor S_1_ onto acceptor OFF [6] (OET) (15) is considered. Energy transfers with T_1_ as donor are disregarded, since their efficiency depends on the rate of phosphorescence [31], which is relatively low if measurements are carried out at room temperature [32].

It is worth emphasizing that energy transfers can lead to a recycling of the acceptor on a relatively small timescale. In the case of STA, T_2_ can either deexcite to S_1_ via RISC (16) or to T_1_ via IC and VR. Unless otherwise stated, we disregard RISC because its probability is much lower and treat STA as [S_1_, T_1_] → [S_0_, T_1_], since the lifetime of T_2_ is negligible [33] compared to S_0_ and T_1_. The rate of back-isomerization from *cis*-Cy5 S_1_ to *trans*-Cy5 S_0_ is increased by CET, so we differentiate the energy transfer in two pathways: [*trans*-Cy5 S_1_, *cis*-Cy5] → [*trans*-Cy5 S_0_, *trans*-Cy5 S_0_] and [*trans*-Cy5 S_1_, *cis*-Cy5] → [*trans*-Cy5 S_0_, *cis*-Cy5], where $k_{CET}$ is the sum of the two transition rates. The recycling efficiency of acceptor states is important to consider, as it determines the acceptor state lifetime, the availability of FRET, and hence the impact of FRET on fluorescence lifetimes and number of emitted photons.

## Results

We used Python-based custom code to run a stochastic simulation algorithm for a continuous-time Markov chain simulation that resembles the photophysical system of Cy5 in all detail or with hidden states as observed experimentally. We assume that photophysical transitions are well described by a continuous-time stochastic process {s(t):t≥0} with s(t)∈S denoting the state at time t in state space S or by a process {u(t):t≥0} with u(t)∈U denoting the transition at time t in transition space U. Both the state and the transition space depend on the total number of interacting fluorophores that make up a photophysical system. Details on the simulation code are given in the Methods section. Details on the available state and transition spaces and corresponding transition matrices that encode the transition rate constants are given in section 1 in S1 Text.

By integrating the current understanding of accessible photophysical states, literature knowledge on rate constants for individual state transitions, and educated guesses for unknown rate constants, precise time traces can be created for all theoretically possible transitions and for observable transitions, which can then be compared with experimental outcomes.

### I. Simulation of single- and multi-fluorophore systems

To demonstrate the output of our simulation machinery, we start with a single reference fluorophore that can populate states and undergo transitions as outlined in Table B in S1 Text (for non-interacting multiple fluorophores, see Fig E in S1 Text; for pulsed excitation, see Fig F in S1 Text). Our presentation scheme for simulation results shows aggregated statistics on all individual states and photophysical transitions (Fig 2A – 2F) as well as on experimentally observable photon emission events and their time series characteristics (Fig 2G – 2L). Simulations cover between 300 and 10000 s depending on the desired sampling accuracy or limited by an irreversible photobleaching step.

Having the full chain of transition times available, one can reduce the data to those transitions that result in observable photon emission events (i.e., fluorescence). The exact time points of fluorescence can be transferred into time traces of binned photon detection times (Fig 2G) to resemble experimentally measured signals from single molecules. They can be analyzed following established experimental techniques, including photoswitching fingerprint analysis (PFA) [6], fitting analytical fluorescence correlation spectroscopy [34] (FCS) models, and thresholding procedures to determine ON/OFF intermittency (Fig 2H – 2L).

We verified the output of our simulation of Cy5 photophysics for models of increasing complexity by comparing it to literature-based experimental data (Figs B and C in S1 Text). We show that the autocorrelation function $g^{\left(\text{2}\right)}$ for photon arrival times indicates sub-Poissonian statistics as expected for a single-photon quantum emitter [35], with $g^{\left(\text{2}\right)}(\text{0})=\text{0}$ for single fluorophores in continuous and pulsed excitation; $g^{\left(\text{2}\right)}(\text{0})\neq \text{0}$ for multi-fluorophore systems; and $g^{\left(\text{2}\right)}(\text{0})=\text{0}$ for multi-fluorophore systems with high SSA efficiencies [28,36]. We successfully simulated experimental data of Widengren et al. [9] and Gidi et al. [11] to verify correct implementation of Cy5 photophysics with and without dSTORM conditions.

For simple systems such as the one shown here, statistics can alternatively be predicted using the limiting distribution of the Markov chain (see section 1.3.2.2 in S1 Text). The output can be very helpful to quickly check for possible inconsistencies in the setup and to get an idea of which parameter changes might have the greatest impact on the observed data. For example, a small lifetime change can be significant if the respective state is visited frequently.

### II. Energy transfer from Cy5 S_1_ to Cy5-SR^-^ adduct can lead to photoswitching fingerprints

Since the simulation outcomes of literature-based models of Cy5 photophysics agree well with their corresponding experimental results, we focus on investigating the underlying photophysical mechanisms in dSTORM conditions that give rise to the experimentally observed different shapes of PFA. While in experiments shorter distances between four Cy5 fluorophores led to a steep curvature in the beginning of the eCDF, larger inter-fluorophore distances or fewer fluorophores led to a more uniform distribution of localization times (see Fig 1g in Helmerich et al. [6]). Fluorophores in close proximity displayed early photobleaching, reducing the effects on blinking behavior and fluorescence lifetime with increasing observation time (see Fig 2J in Helmerich et al. [6]). The question is whether it is possible to obtain information about inter-fluorophore distances in molecular systems from PFA.

**Fig 3:**
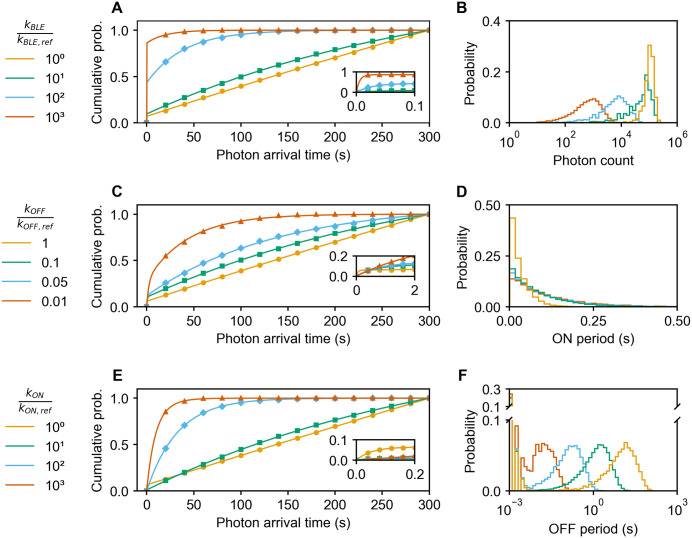
Parameters that control the shape of PFA of single Cy5 fluorophores. **(A, B)** Altering the photobleaching rate $k_{BLE}$. **(C, D)** Altering the rates associated with entering the OFF state ($k_{OFF}=k_{PET_{SO}}+k_{PET_{TO}}$, see Table B in S1 Text). **(E, F)** Altering the rates associated with entering the ON state ($k_{ON}=k_{PU}+k_{TE}$, see Table B in S1 Text). (**A, C,** **E)** The eCDF of photon arrival times. The markers depict simulated results. The analytical expression (solid lines) and fitting procedure are described in section 8 in S1 Text. **(B)** Probability histogram of total photon counts during 300 s. **(D)** Probability histogram of ON periods. **(F)** Probability histogram of OFF periods. A full set of all corresponding plots is provided in Fig H in S1 Text. For adjusting $k_{OFF}$ via thiol concentration, see Fig I in S1 Text.

To identify the principle behind the different shapes of PFA, we started with varying the parameters of a single fluorophore. Here we identified two major pathways to achieve PFA with steep curvatures: (1) Photobleaching rates are increased, the rates of photobleaching competitors are decreased, or additional photobleaching pathways become available (Fig 3A, 3B). (2) Increased number of excitation cycles per time (except homo-FRET induced cycles) due to a mechanism that does not compete with photobleaching. This can further be subdivided into (2.1) lower OFF state probability (Fig 3C, 3D) and (2.2) lower OFF state lifetime (Fig 3E, 3F). (Note that in general, (2) could be influenced by lifetimes and probabilities of states other than the OFF state, but since the measurements are on a timescale of several hundred seconds, the OFF state must be involved.) What all these pathways have in common is that they influence the time at which the fluorophore photobleaches, since (1) and (2.1) increase the chance of photobleaching per ON state and (2.2) increases the rate of occurrence of the ON state. In other words, the global bleaching rate is increasing (Fig G and section 8 in S1 Text). A linear eCDF indicates that there were no photobleaching events during the observation time.

We clearly see a dependence on inter-fluorophore distances indicating an energy transfer mechanism responsible for the increased global bleaching rate. Increased photobleaching rates (1) could then be due to photoionized fluorophores as a product of SSA and STA that have an increased probability of photobleaching [37]. This would also result in a significantly lowered number of total photons emitted by a fluorophore before photobleaching [38]. Lower OFF state probability (2.1) could be due to STA, where T_2_ can undergo RISC [39] to end up in S_1_, thereby lowering the chance to enter the OFF state via T_1_. Lower OFF state lifetime (2.2) could be due to OET [6], which (at least partially) yields S_0_. There are other effects such as fluorophore dimerization [40], photoinduced electron transfer [41] or the general alteration of the chemical environment that are also distance dependent. However, a shorter fluorescence lifetime in photoswitching compared to trolox buffer at inter-fluorophore distances of 3 nm and visible effects even at 6 nm distances provide further evidence of a Förster resonance energy transfer as origin for PFA signature. This also implies that the energy transfer is dSTORM-specific, promoting OET to be the most likely candidate. Furthermore, photoionization and RISC were ruled out by both simulation and theoretical considerations (Figs J and K in S1 Text). Therefore, we proceed under the assumption that OET is the main cause for inter-fluorophore distance dependent alterations of PFA.

**Fig 4:**
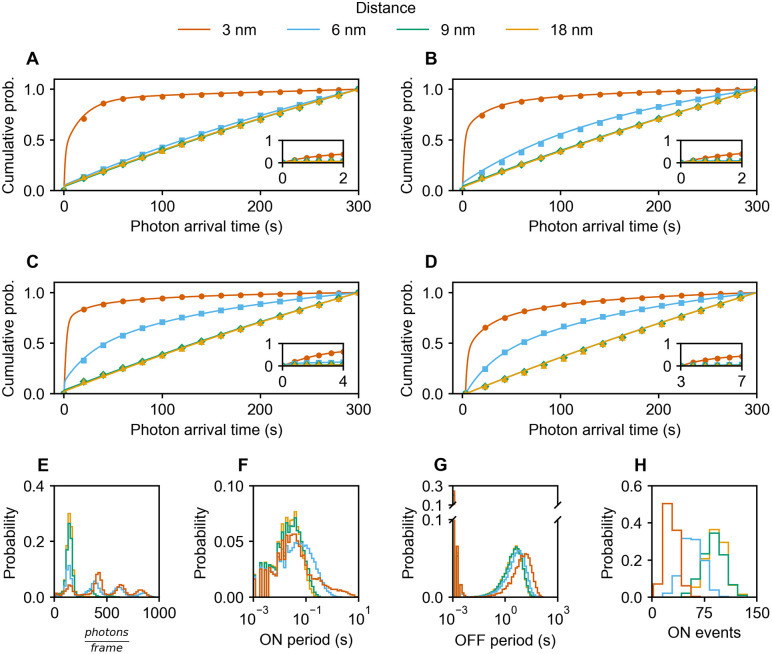
PFA of multi-fluorophore systems undergoing OET. **(A)** Two fluorophores, **(B)** three fluorophores, **(C – H)** four fluorophores. The photophysical model includes OET leading the OFF state to transition to S_0_ with a probability of 0.01%. **(A – D)** The eCDF of photon arrival times. In case of **(D)**, the data is truncated such that only times ≥ 3 s are included to mimic acquisition in the beginning of an experiment. The markers depict simulated results. The analytical expression (solid lines) and fitting procedure are described in section 8 in S1 Text. **(E)** Probability histogram of photon counts per frame. **(F)** Probability histogram of ON periods. **(G)** Probability histogram of OFF periods. **(H)** Probability histogram of number of ON periods, i.e., ON events. A full set of all corresponding plots is provided in Fig L in S1 Text. Representative fluorescence time traces are provided in Fig M in S1 Text. Representative photophysical statistics of four fluorophores at 3 nm are provided in Fig N in S1 Text.

The Cy5-SR^-^ absorption [11] at wavelengths corresponding to the Cy5 emission results in a small spectral overlap integral compared to that of standard energy transfer partners. However, given the long lifetime of the OFF state, inefficient FRET can have a significant impact. In fact, our simulations suggest an even lower efficiency of 0.01% of OET transitions that cause the OFF state to return to the ON state (Fig 4). While not quantitatively identical, our estimation remains within a reasonable order of magnitude compared to experimentally reported results (see section 7.1 in S1 Text).

The dependence of PFA shape on both the distance and the number of fluorophores is demonstrated for 2- (Fig 4A), 3- (Fig 4B) and 4-fluorophore systems (Fig 4C, 4D), where shorter distances and higher fluorophore numbers result in a steeper initial rise of the PFA. The fluorophore configurations are depicted in Fig A in S1 Text. The simulated PFAs can be accurately fit using an analytical description derived in Section 8 in S1 Text. Experimentally, information about the very beginning may be lost due to acquisition as mimicked in Fig 4D. An equivalent figure using localization detection times rather than photon arrival times is shown in Fig V in S1 Text.

Incorporating OET also leads to the experimentally observed effects of different irradiances (Fig O in S1 Text). Nevertheless, some quantitative aspects of the simulation deviate from the corresponding experimental observations: the simulation generated too large fluorescence lifetimes (Table 1, Table D in S1 Text), not enough short OFF periods (Fig 4G), and too high photon counts (Fig 4E) for short inter-fluorophore distances [6] (also see sections 7.2 and 7.4 in S1 Text). g^(2)^(0) of 6 nm also deviated from experimental results (Fig R in S1 Text). We maintained strict adherence to parameter values reported in the literature and derived from rigorous theoretical considerations. Only the photobleaching rate and the OET efficiency were fully adapted to our own experimental data. Given this approach, a certain degree of discrepancy is to be expected. However, we want to emphasize that these discrepancies do not challenge the central conclusion of our study, as OET is inevitably required to produce experimentally observed distance dependent PFAs in any case. Without OET, the ON states that coincide at the beginning of a measurement quickly fade as fluorophores enter the OFF state, where they can no longer serve as donor nor acceptor. Given the long lifetime of OFF and short lifetime of ON states, the probability of stochastically coinciding ON states is low, causing energy transfers to no longer occur, and hence no distance dependent effects would be observable.

**Table 1:** Fluorescence lifetimes in different simulated conditions. The fluorescence lifetimes are recorded using only S_1_ durations that deexcite via fluorescence. Each condition is simulated 10 times for 10^8^ steps. N is the number of fluorophores, the distance is set to 3 nm, photobleaching is disabled. The adjustment numbers represent different changes to the photophysical model, either qualitatively or quantitatively: 1 – $k_{CET}$ × 0.01 and $\eta_{CET_{2}}$ is 0, $k_{STA}$ × 0.1, i.e., CET and STA are set such that their impact on fluorescence lifetime is maximized (see Fig Q in S1 Text); 2 – energy transfer to R ($k_{\;3nm}=10^{9}s^{-1}$), where 2.1 only considers S_1_|R to S_0_|R, 2.2 also considers S_1_|R to S_0_|S_0_ with efficiency 10^-4^ and 2.3 with efficiency 10^-3^; 3 – $k_{PET_{TR}}$ × 10; 4 – $k_{PET_{TR}}$ × 100. A more detailed version of this table is provided in Table D in S1 Text.

ID	N	Adjustment	Energy transfer	${\overset{―}{\tau }}_{FLU}\;(10^{-9}s)$	$\sigma_{\tau_{FLU}}\;(10^{-13}s)$
1	4	–	–	1.69	4.9
2	4	–	+	1.63	19.6
3	4	1	+	1.17	14.4
4	4	2.1, 3	+	1.14	47.5
5	4	2.1, 4	+	0.72	37.0
6	3	2.1, 4	+	0.92	32.5
7	2	2.1, 4	+	1.07	41.2
8	4	2.2, 4	+	0.81	32.9
9	4	2.3, 4	+	1.24	39.8

### III. Energy transfer with the radical anion as acceptor explains fluorescence lifetimes

To resolve the remaining discrepancies, we focused on fluorescence lifetimes $\tau_{FLU}$, as they are largely independent of instrumental parameters such as photon collection efficiency, frame integration time or detection threshold. While experimentally observed fluorescence lifetimes dropped from 1.7 ns for single fluorophores to 0.7 ns for 4-fluorophore systems at 3 nm distance, simulated fluorescence lifetimes dropped only to 1.63 ns (Table 1 ID1, ID2). We first relaxed the strict adherence to literature parameter values that guided previous analyses and explored a broader parameter space. The energy transfers CET and STA have large spectral overlaps with Cy5 emission [11] and have corresponding rate constants that largely suppress photon emission. However, even when setting their acceptor state recycling efficiency to 1 and varying their rates such that the effect on fluorescence lifetime is at a maximum, $\tau_{FLU}$ does not decrease below 1 ns (Table 1 ID3). Therefore, the only way to further increase the influence of CET and STA on $\tau_{FLU}$ is by increasing the lifetimes or formation rates of *cis* and T_1_, making CET and STA unlikely to be the reason for the experimentally observed $\tau_{FLU}$. The rate constant of OET and its efficiency of converting the acceptor OFF state to an ON state must result in a certain minimum rate for $k_{OET_{\text{2}}}$ such that OFF state rescue can occur before the donor ON state also transitions to OFF. Therefore, without increasing $k_{OET}$ to a degree that any photon emission becomes unlikely, the OFF to ON efficiency cannot be decreased further. We conclude that our currently implemented energy transfers alone cannot explain the experimentally observed fluorescence lifetimes.

Instead, our simulations show that only when considering an additional energy transfer with the radical anion R as acceptor, $\tau_{FLU}$ can indeed drop to 0.7 ns (Table 1 ID5). The spectral overlap of R absorption and Cy5 emission was assumed to be 5-fold smaller than that of OFF, corresponding to molar extinction coefficients of around 10^3^ M^-1^ cm^-1^. It was further assumed that efficient IC and VR take place [42], allowing for acceptor state recycling following energy transfer (Table 1 ID5, ID8, ID9); and that the rate for the formation of R is increased (Table 1 ID4 – 9), which may be the case for MEA-mediated PET (see section 1.2 in S1 Text). Incorporating FRET to R does not only solve our issue with $\tau_{FLU}$, but also decreases photon counts (Figs T and U in S1 Text) and provides the basis for shorter OFF period populations (Fig T in S1 Text) and improved agreement of second order coherence analyses (Fig R in S1 Text) at short inter-fluorophore distances, while maintaining the PFA shapes (Fig T in S1 Text).

## Discussion

PFA led to the discovery that multiple fluorophores in dSTORM conditions communicate at inter-fluorophore distances of less than 10 nm. Our study shows that this communication can be attributed to a FRET from Cy5 S_1_ to the long living OFF state Cy5-SR^-^, thereby increasing its rate to return to Cy5 S_0_. The energy transfer that we call OET in turn leads to an increased global bleaching rate, which is clearly visible in PFA. With simulations of various photophysical models, we confirmed that alternative energy transfer processes different from OET (e.g., with T_1_ as acceptor) are insufficient to reproduce experimental observations.

Our results support the idea that a very small FRET efficiency is sufficient to significantly alter PFA signatures, which is in line with absorption cross sections of photoinduced uncaging of Cy5-SR^-^ as inferred from data reported by Gidi et al. [11] More generally, our results emphasize blinking fluorophores with long living OFF states that absorb many orders of magnitude less than standard FRET acceptors to be possible candidates for sub-10 nm reporter systems.

In dSTORM, spatial resolution is increased via temporarily separating ON states of individual fluorophores. OET disrupts this separation as it results in coinciding ON states. Therefore, if labeling densities are high, OET prevents reliable assignment of localizations to individual fluorophores. However, PFA reveals the presence of OET providing information about fluorophores with sub-10 nm interfluorophore distances. Furthermore, our findings indicate that additional energy transfers are necessary to explain all experimental data. We propose the radical anion of Cy5 to be the most likely candidate as FRET acceptor for full agreement with experimental data. The combination of a vanishingly small absorption overlap integral and the high availability promote FRET to R as the major contributor to decreased fluorescence lifetimes at 3 nm distances.

The simulation machinery can be used not only to verify that OET-including photophysical models agree with experimental data, but also to predict PFA signatures for various fluorophore arrangements. Hence, our simulation tool can be used to identify the best fluorescent labels and arrangements for optimized sub-10 nm dSTORM imaging. It expands the photophysics simulation software landscape [43–46] by providing a Python-based option to investigate effects of multiple energy transfer mechanisms applied to systems of up to four different or identical fluorophores.

## Conclusion

In conclusion we developed a simulation package to derive photon arrival statistics for multi-fluorophore systems that can be compared to observed signals in dSTORM experiments. A comprehensive discussion of all relevant electronic states and transition probabilities and comparison of simulation results from various photophysical models for Cy5 confirmed that energy transfer between ON and OFF states results in photon arrival statistics that report on inter-fluorophore distances and the number of fluorophores in multi-fluorophore systems. Taking fluorescence lifetimes into consideration, simulations provide unambiguous evidence for the existence of an additional energy transfer to the radical anion. The detailed simulation results or alternatively an analytical description of the overall photon arrival times in multi-fluorophore systems will serve as foundation for further, possibly artificial intelligence-based analysis of experimental PFA signatures.

## Methods

### Computer simulation

Single-molecule fluorescence signals were simulated as continuous-time Markov chains (see section 1.3 in S1 Text for more details) with custom-made Python code and with use of libraries from the scientific Python stack (NumPy [47], SciPy [48], Matplotlib [49], Pandas [50]). The analysis pipelines were set up in Jupyter notebooks. For random number generation we used the NumPy PCG-64 pseudo-random number generators. When running multiple simulations in parallel we made use of ray (https://github.com/ray-project/ray) with appropriate seeding for the random number generation in parallel jobs. From all simulated transition chains and corresponding time points, the observable emission events (photon detection times) were extracted and treated by further analysis procedures resembling those for experimental data. This includes multiple-tau correlation in FCS [51,52] and time trace blinking analysis [53]. Photophysical models were displayed using NetworkX [54].

All simulations were carried out on a personal computer equipped with Intel Core Ultra 7 265 CPU and 32 GB RAM. Simulations were implemented in Python 3.13. There were no GPU requirements.

### Simulation parameters

The simulation results depend on imaging-related parameters as well as on the rate constants of photophysical transitions. Precise values of the latter are given in the code and Table B in S1 Text. If not stated differently, the following remarks apply: (i) The excitation laser is set to 640 nm with an irradiance of 2.5 kW cm^-2^. The excitation light is assumed to be randomly polarized. (ii) Rotational diffusion is assumed to take place on the picosecond timescale (rotational correlation time $\tau_{c}\approx \text{5}\text{0}\text{0}\;ps$) without any hinderance. Due to the fast rotation, photons are assumed to be emitted in random direction. (iii) The numerical aperture NA of the objective is set to 1.45, the refractive index n of the medium to 1.51. The photon collection efficiency is calculated as $\text{2}\pi *\frac{\text{1}-\text{cos}\theta }{\text{4}\pi }=\text{0}.\text{3}\text{6}$, where $\theta ={\text{sin}}^{-\text{1}}\frac{NA}{n}$. The optical path consists of a bandpass filter (665 nm – 731 nm, corresponding to an efficiency of 0.54), a 90/100 mirror and two lenses, each with an assumed transmittance of 0.99. The quantum efficiency of the camera is set to 0.85. Each of the values are used as the probability parameter in a binomial distribution to yield the number of detected photons. The total detection efficiency is hence 0.146. In this study we do not convert the photons into ADUs and hence neglect the effect of first applying the EMCCD gain distribution and then calculating back the number of photons. However, we do consider the effect of dark current noise (Poisson noise) and the application of a photon count threshold. The frame integration time is set to 1 ms. (iv) We consider attributes of mercaptoethylamine to calculate the rate constants with which fluorophores enter the OFF state. The pK_a_ is set to 9.0, the concentration to 100 mM and the pH of the buffer to 7.5.

### PFA estimation

PFA signatures were derived from simulated data for the various fluorophore systems. Note that within a simulated PFA, no variation of fluorophore count was applied. For Fig 3, data from 100 to 15000 time series were combined for each condition, depending on the number of photons emitted during the time window (see insets of Fig G in S1 Text). For Fig 4, data from 500 time series were combined for each condition.

## Supporting information

S1 Text Methods, simulations, validation of the simulation framework, additional results, and analytical expression of PFA. Includes supplementary figures (A – X) and supplementary tables (A – E).
(PDF)
