# Supplementary material for: Energy transfer leaves fingerprints in cyanine photoswitching behavior
Source: PLoS Comput Biol. 2026 May 18;22(5):e1014322. doi: 10.1371/journal.pcbi.1014322 (PMC13197066; doi:10.1371/journal.pcbi.1014322)
Supplement: S1 Text — Includes supplementary figures (A – X) and supplementary tables (A – E). (PDF) [file pcbi.1014322.s001.pdf]

# - Supporting Information -

# Energy transfer leaves fingerprints in cyanine photoswitching behavior

Vincent Ebert[1], Markus Sauer[1,2], Sören Doose[1]*

**Affiliations**

[1]Department of Biotechnology and Biophysics, Biocenter, Julius-Maximilians University, 97074 Würzburg, Germany

[2]Rudolf Virchow Center, Research Center for Integrative and Translational Bioimaging, Julius-Maximilians University, 97074 Würzburg, Germany

* soeren.doose@uni-wuerzburg.de

## Sections

1 Methodological framework and simulation setup ........ 4

1.1 Multi-fluorophore configurations ........ 4

- Fig A. Multi-fluorophore configurations. ........ 4

1.2 Cy5 photophysical processes and kinetic rate constants........ 4

- Table A. Photophysical properties of Cy5. ........ 7
- Table B. Implemented transitions and their kinetic rate constants. ........ 8

1.3 Continuous-time Markov chain simulation ........ 9

1.3.1 Method description ........ 9

1.3.2 Continuous excitation ........10

1.3.3 Pulsed excitation ........11

1.3.4 Varying transition matrix ........12

1.4 Caveats ........12

2 Validation of simulation framework ........14

- Fig B. Validation of the simulation framework part I. ........14
- Table C. Irradiance, oxygen or thiol effect on the lifetimes of S0, cis and T1, and the rate of ISC from S1 to T1. ........15
- Fig C. Validation of the simulation framework part II. ........16

3 Photophysical statistics: Effects of analysis parameters, fluorophore multiplicity, and excitation mode ........17

- Fig D. OFF and ON periods vs. OFF and ON states. ........17
- Fig E. Photophysical statistics of four non-interacting fluorophores. ........18
- Fig F. Photophysical statistics of a single fluorophore with pulsed excitation. .20

4 PFA in single-fluorophore systems........22

- Fig G. Global bleaching times of different single-fluorophore systems. ..........22
- Fig H. Extended analysis of Fig 3: Parameters that control the shape of PFAs of single fluorophores. ..........23
- Fig I. Effects of alterations in thiol concentrations. ..........24

5 PFA in multi-fluorophore systems – disproving alternatives to OET ..........26

- Fig J. PFA of 4-fluorophore systems that yield photoionized fluorophores following STA. ..........26
- Fig K. PFA of 4-fluorophore systems undergoing RISC following STA. ..........27

6 Multi-fluorophore systems exhibiting OET ..........29

- Fig L. Extended analysis of Fig 4 part I: Statistics of multi-fluorophore systems undergoing OET. ..........29
- Fig M. Extended analysis of Fig 4 part II: Fluorescence time traces of multi-fluorophore systems undergoing OET. ..........30
- Fig N. Photophysical statistics of four fluorophores at 3 nm distance with energy transfers enabled. ..........31
- Fig O. Effects of different irradiances on fluorescence time traces. ..........32

7 Multi-fluorophore systems exhibiting OET and FRET to the radical anion ..........33

7.1 Effective absorption cross section of OET ..........34

- Fig P. PFA of 4-fluorophore systems exhibiting OET in different variations. ..........34

7.2 Fluorescence lifetimes ..........36

- Fig Q. Rate constants of STA and CET and corresponding fluorescence lifetimes. ..........36
- Table D. Fluorescence lifetimes in different simulated conditions. ..........36

7.3 Second order coherences ..........39

- Fig R. Second-order coherence of four fluorophores in different simulated conditions. ..........39
- Fig S. Fluorescence time traces of four fluorophores in different simulated conditions. ..........41

7.4 Adjusted photophysical model ..........42

- Fig T. PFA and statistics of 4-fluorophore systems exhibiting OET and energy transfer to R. ..........42
- Fig U. Fluorescence time traces of 4-fluorophore systems exhibiting OET and energy transfer to R. ..........43

8 PFA analytical expression ..........44

8.1 Assumptions ..........44

- Fig V. PFA of multi-fluorophore systems undergoing OET using localization times instead of photon arrival times. ..........45

8.2 Derivation ..........48

- Fig W. Modeling of PFAs. ..........48

8.3 Properties ........................................................................................................50
8.4 Fitting procedure and parameters ..................................................................51
- Table E. PFA Model parameters of simulated multi-fluorophore systems at 3 nm distance. ............................................................................51
- Fig X. Fitting PFA of n fluorophores at 3 nm distance with parameters yielded by fitting PFAs of m > n fluorophores. ...............................................52

9 References..........................................................................................................53

# 1 Methodological framework and simulation setup

## 1.1 Multi-fluorophore configurations

For simulations of multi-fluorophore systems, the 4-fluorophore arrangement is modeled as a square (Fig AA), where the specified distance corresponds to the side length ($r_1$). The 3-fluorophore arrangement is modeled as an isosceles right triangle (Fig AB), with the given distance again corresponding to $r_1$. The 2-fluorophore system includes only a single distance parameter (Fig AC). Notably, the 4- and 3-fluorophore configurations eventually decay into either the Fig AC or AD.

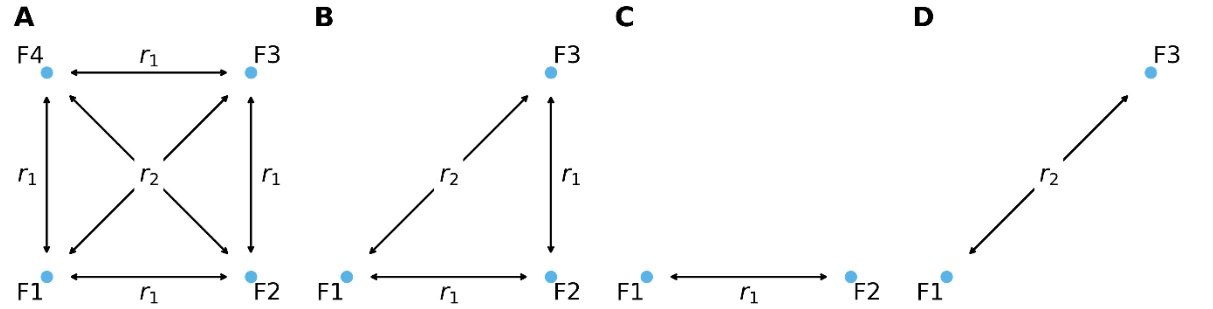


**Fig A. Multi-fluorophore configurations.** The 4- (A), 3- (B), and 2- (C) fluorophore configuration is shown. Given distances refer to $r_1$. As photobleaching progresses, (A) decays to (B); however, (B) can decay to either (C) or (D).

## 1.2 Cy5 photophysical processes and kinetic rate constants

The following photophysical processes and related kinetic rate constants are considered for explaining and simulating the photophysical system resembling Cy5:

**Excitation (EXC):** The excitation rate can be calculated using the excitation cross section $\sigma_{EXC}$. The scattering cross section of Cy5 is assumed to be negligible (as it is for FITC[1]). Hence, $\sigma_{EXC}$ is equal to the attenuation cross section $\sigma$ or can be expressed with the corresponding extinction coefficient ε with $\sigma = \frac{\varepsilon * \ln(10)}{N_A}$. The excitation rate is then $k_{EXC} = \sigma * \Phi$, where $\Phi$ is the photon flux that can be determined using the irradiance $E_e$ like $\Phi = \frac{E_e}{h\nu}$.

**Fluorescence (FLU):** The rate of fluorescent emission is $k_{FLU} = \frac{\Phi_{FLU}}{\tau_{S1}}$, where $\Phi_{FLU}$ is the fluorescence quantum yield and $\tau_{S1}$ is the $S_1$ lifetime.

**Internal conversion (IC):** The IC rate can be determined with $k_{IC} = \frac{k_{FLU}}{\Phi_{FLU}} - \sum_i k_{S1,i}$, where $k_{S1,i}$ are all S1-outgoing transitions except $k_{IC}$. It is followed by vibrational relaxation (VR), which is fast enough to be neglected here. The transition also represents twisted state relaxations of *trans* $S_1$ to *trans* $S_0$.

**Intersystem crossing (ISC):** ISC followed by VR can occur from $S_1$ to $T_1$ or from $T_1$ to $S_0$. The total $T_1$ to $S_0$ rate depends on the concentration of molecular oxygen. Here we mimic an oxygen-depleted environment.

**Isomerization (ISO):** The rate of isomerization from *trans* to *cis* is a sum of thermally and photoinduced transitions. However, at standard excitation conditions for Cy5, the contribution of thermally induced ISO is negligible. Note that we mimic Cy5 coupled to DNA (the coupling decreases the ISO rate compared to free Cy5[2]).

**Back-isomerization (BISO):** The rate of effective back-isomerization consists of a thermally induced (TBISO) and a photoinduced (PBISO) component, where the photoinduced component can be calculated as $k_{cis \rightarrow trans} = \sigma_{BISO} * \Phi$ with $\sigma_{BISO}$ being the effective back-isomerization cross section assuming the excited state lifetime of *cis* is negligible. We used an estimate of $\sigma_{BISO,640\ nm} = 0.6 * 10^{-17} cm^2$ since we mimic Cy5 coupled to DNA (DNA decreases the cross section compared to free Cy5, 640 nm increases the cross section compared to 632 nm). Note that lifetime measurements of *cis* hence depend on potential illumination, e.g., the difference in Widengren et al.[2] and Gidi et al.[3] can be attributed to the former using continuous excitation whereas the latter using flash photolysis.

**Photobleaching (BLE):** Here it is important to consider whether the environment is oxygen depleted or not, since oxygen-mediated photobleaching is the main pathway in non-depleted environments. Collisional $T_1$ quenching by oxygen is an important factor for determining the triplet lifetime[4]. Here we assume an oxygen-depleted environment and use a photobleaching rate that is consistent with the survival duration of single fluorophores. To be precise, linear PFA of single fluorophores over a duration of 300 s provide an upper bound for the photobleaching rate. A lower bound is established by the requirements imposed by the effective absorption cross section of OET (see section 7.1).

**OFF state formation via photoinduced electron transfer (PET):** The dSTORM-specific photoinduced electron transfer rate $k_{PET}$ is dependent on the base concentration of the reducing agent, which can be determined using the Henderson-Hasselbalch equation $[A^-] = 10^{pH-pKa} * \frac{c}{10^{pH-pKa}+1}$, where $c$ is the concentration of the reducing agent[3]. It is then $k_{PET} = k_{PET,M} * [A^-]$, where $k_{PET,M}$ is the concentration independent rate constant of PET. PET can occur in $S_1$ or $T_1$ and eventually gives rise to either BET or GRC, where BET occurs 3 orders of magnitude more often than GRC. In the case of $T_1$, PET can alternatively be followed by RE. We describe all these processes – |PET($S_1$),GRC| ($PET_{SO}$), |PET($S_1$),BET| ($PET_{SS}$), |PET($T_1$),ISC,GRC| ($PET_{TO}$), |PET($T_1$),ISC,BET| ($PET_{TS}$), |PET($T_1$),RE| ($PET_{TR}$) – as PET, since their rates scale proportionally (we assume GRC, BET and RE to be constant throughout different experimental conditions). Note that only |PET($S_1$),GRC| and |PET($T_1$),ISC,GRC| lead to OFF state formation.

**ON state formation:** This transition consists of thermal elimination (TE) and photoinduced uncaging (PU). We estimated the effective uncaging cross section to be $\sigma_{PU} = 6 * 10^{-24}\ cm^2$ (corresponding to $\varepsilon = 1.6 * 10^{-3} M^{-1} cm^{-1}$) given the increase in $k_{OFF \rightarrow S_0}$ by 0.02 $s^{-1}$ using 1 kW $cm^{-2}$ (Fig 4 c of Gidi et al.[3]), neglecting the excited state lifetime of OFF.

**Radical escape (RE) following PET:** Gidi et al.[3] observed no significant formation of Cy5 radical anion (R) via β-mercaptoethanol-mediated PET. Srambickal et al.[5] assigned a rate constant of $3 * 10^5 s^{-1}$ for formation of R via $T_1$ using AF647 and 10 mM mercaptoethylamine. We calculated the rate $k_{PET}^{T_1} = 3 * 10^5 s^{-1}$ assuming 100 mM mercaptoethylamine, however this rate represents the sum of |PET($T_1$),ISC,GRC|, |PET($T_1$),ISC,BET|, |PET($T_1$),RE|. Hence, we need to estimate the efficiency of RE. We assume that the choice of fluorophores has a greater impact on the electron transfer behavior than the specific thiol employed. Therefore, we choose $\eta_{RE} = \frac{k_{RE}}{k_{ISC,BET}+k_{ISC,GRC}+k_{RE}} = 10^{-2}$, which is small enough to be insignificant in transient absorption spectroscopy[3].

**Oxidation (OXI):** The lifetime of the radical anion depends on the presence of oxidizing agents such as molecular oxygen. Here we mimic an oxygen-depleted environment.

**Förster Resonance Energy Transfer (FRET) rates:** FRET rates can be calculated using $k_{FRET} = 8.875 * 10^{-11} * \frac{\kappa^2 * k_{FLU}}{n^4 * r^6} * J$, where $\kappa^2$ is the dipole orientation factor, $k_{FLU}$ is the

fluorescence rate of the donor, $n$ is the refractive index, $r$ is the distance and $J$ is the spectral overlap integral[6]. Except in Table D ID10 and ID11, $\kappa^2$ is set to $\frac{2}{3}$, assuming freely, fast rotating and hence randomly oriented pairs of molecules. In Table D ID10 and ID11, the value of $\kappa^2$ varies for different $S_1$. $J$ depends on the emission spectrum $F(\lambda)$ and the absorption spectrum $\varepsilon(\lambda)$ according to $J = \int F(\lambda) * \varepsilon(\lambda) * d\lambda * \lambda^4$. We estimated $J$ using the absorption spectra of Cy5 and its related species[3] (e.g., *cis*, OFF, $T_1$).

**Singlet-singlet annihilation (SSA):** This energy transfer's rate is difficult to be determined since there is no information about the absorption spectrum of $S_1$ available. The energy associated with $S_2$ is around 3.3 to 4 eV, resulting in an energy gap to $S_1$ of $\Delta E \approx 1.8\ eV$. Since this corresponds to the energy of photons emitted by $S_1$, the relative spectral overlap is expected to be high. However, without the transition dipole moment of $S_1$ to $S_2$, the absolute value can only be estimated. We treat $S_2$ to instantly transition to $S_1$ via VR, such that the energy transfer can be described with $S_1|S_1 \rightarrow S_0|S_1$. Given the low lifetime of $S_1$, the acceptor is only very rarely available. Hence, it plays a role for determining the degree of photon antibunching but other than that, SSA is negligible.

**Singlet-triplet annihilation (STA):** Except in Fig J and K, we treat STA[7] as $S_1|T_1 \rightarrow S_0|T_1$, i.e., with an instant transition of $T_2$ to $T_1$ following the energy transfer. In Fig J, a subset of all STA transitions is treated as $S_1|T_1 \rightarrow S_0|B$, resembling photoionization and subsequent photobleaching. In Fig K we simulate RISC via STA as $S_1|T_1 \rightarrow S_0|S_1$.

**FRET of *trans*-$S_1$ to *cis*-$S_0$ (CET):** This energy transfer is differentiated in two components to neglect the exact photophysical states of the *cis* isomer. Hence, the energy transfer can either lead *cis* to transition to *trans* $S_0$ or to stay in *cis*, resembling IC/VR. However, the ratio of *cis* $S_1$ undergoing VR to BISO remains to be estimated. Gidi et al.[3] report a molar extinction coefficient $\varepsilon_{cis,645\,nm} = 1 * 10^4 M^{-1} cm^{-1}$, corresponding to $\sigma_{cis,645\,nm} = 3.8 * 10^{-17} cm^2$. Widengren et al.[2] report a $\sigma_{BISO,645\,nm} = 1.7 * 10^{-17} cm^2$. This would correspond to $\Phi_{cis \rightarrow trans} = \frac{\sigma_{BISO}}{\sigma_{cis}} = 0.44$, whereas $\Phi_{trans \rightarrow cis} = 0.02$ (both for free Cy5). Of course, there can be a difference between their quantum yields, e.g., when looking at established energy landscapes the rotational barrier for *cis* is depicted smaller[8]. However, there is also the argument that *cis* is dim due to efficient internal conversion, which would imply a low $\Phi_{cis \rightarrow trans}$. Finally, the absorption spectrum for *cis* given by Gidi et al. could underestimate the absorption at around 645 nm due to the assumption of no species except Cy5 absorbing at this wavelength. Hence, estimating $\Phi_{cis \rightarrow trans}$ comes with a great amount of uncertainty. We decided to set $\Phi_{cis \rightarrow trans}$ to 0.04, assuming $\varepsilon_{cis,645\,nm}$ to be $1 \times 10^5\ M^{-1}\ cm^{-1}$. Either way, we can expect small *cis* populations at short inter-fluorophore distances (in the case of dSTORM conditions, given that different fluorophore's ON states coincide) due to the large spectral overlap.

**FRET of $S_1$ to OFF (OET):** Like CET, we differentiate OET in two components, one causing the OFF state to transition to $S_0$ and another one inducing no change in the OFF state, resembling OFF* undergoing VR. The ratio of these two was estimated comparing PFAs to corresponding experimental data (for details, see main article and SI section 7.1).

**Table A. Photophysical properties of Cy5.** E = Estimated (the value was estimated based on observations and assumptions described in SI section 1.2).

| Parameter | Value | Reference |
|---|---|---|
| $\sigma_{EXC,640\,nm}$ | $9 \times 10^{-16}$ cm$^2$ | 2 |
| $\Phi_{FLU}$ | 0.27 | 9 |
| $\tau_{S1}$ | $1.7 \times 10^{-9}$ s | 10 |
| $\sigma_{BISO,640\,nm}$ | $0.6 \times 10^{-17}$ cm$^2$ | 2 |
| $\sigma_{PU,640\,nm}$ | $6 \times 10^{-24}$ cm$^2$ | E |
| $k_{PET,T_1,M}$ | $1 \times 10^{8}$ s$^{-1}$ M$^{-1}$ | 3 |
| $k_{PET,S_1,M}$ | $1 \times 10^{9}$ s$^{-1}$ M$^{-1}$ | 3 |
| $\eta_{RE}$ | 0.01 | E |
| $J_{OFF}$ | $1.2 \times 10^{15}$ nm$^4$ M$^{-1}$ cm$^{-1}$ | E |
| $J_{T_1}$ | $8.9 \times 10^{15}$ nm$^4$ M$^{-1}$ cm$^{-1}$ | E |
| $J_{cis}$ | $3 \times 10^{16}$ nm$^4$ M$^{-1}$ cm$^{-1}$ | E |
| $J_{S_0}$ | $1.6 \times 10^{16}$ nm$^4$ M$^{-1}$ cm$^{-1}$ | E |
| $J_{S_1}$ | $3 \times 10^{16}$ nm$^4$ M$^{-1}$ cm$^{-1}$ | E |

**Table B. Implemented transitions and their kinetic rate constants.** The values of energy transfers (transitions with multiple initial and final states) are given for 3 nm distances. The irradiance dependent transitions (*) are given for irradiances of 2.5 kW cm$^{-2}$ at 640 nm. The thiol-mediated PET transitions are given for 100 mM mercaptoethylamine. I = Inferred (the value was calculated using Table A and methods described in SI section 1.2), E = Estimated (the value was estimated based on observations and assumptions described in SI section 1.2).

| ID | Transition | Initial state | Final state | ID in Fig 1 | Value ($s^{-1}$) | Reference |
|---|---|---|---|---|---|---|
| 1 | EXC (*) | $S_0$ | $S_1$ | 1 | $7.3 \times 10^6$ | I |
| 2 | FLU | $S_1$ | $S_0$ | 2 | $1.6 \times 10^8$ | I |
| 3 | IC | $S_1$ | $S_0$ | 3 | $4.2 \times 10^8$ | I |
| 4 | $ISC_{ST}$ | $S_1$ | $T_1$ | 4 | $8.3 \times 10^5$ | [3] |
| 5 | $ISC_{TS}$ | $T_1$ | $S_0$ | 5 | $5 \times 10^3$ | [3] |
| 6 | ISO | $S_1$ | *cis* | 6 | $4 \times 10^6$ | [2] |
| 7 | TBISO | *cis* | $S_0$ | 7 | $5 \times 10^3$ | [3] |
| 8 | PBISO (*) | *cis* | $S_0$ | 7 | $4.8 \times 10^4$ | I |
| 9 | BLE | $T_1$ | B | 8 | $1 \times 10^1$ | E |
| 10 | $PET_{SS}$ | $S_1$ | $S_0$ | 9 | $3 \times 10^6$ | I |
| 11 | $PET_{SO}$ | $S_1$ | OFF | 9 | $3 \times 10^3$ | I |
| 12 | $PET_{TS}$ | $T_1$ | $S_0$ | 9 | $3 \times 10^5$ | I |
| 13 | $PET_{TO}$ | $T_1$ | OFF | 9 | $3 \times 10^2$ | I |
| 14 | $PET_{TR}$ | $T_1$ | R | 9 | $3 \times 10^3$ | I |
| 15 | TE | OFF | $S_0$ | 10 | $1 \times 10^{-2}$ | [3] |
| 16 | PU (*) | OFF | $S_0$ | 10 | $4.8 \times 10^{-2}$ | I/E |
| 17 | OXI | R | $S_0$ | 11 | $1.3 \times 10^3$ | [3] |
| 18 | SSA | $S_1$\|$S_1$ | $S_0$\|$S_1$ | 12 | $1.2 \times 10^{11}$ | I/E |
| 19 | STA | $S_1$\|$T_1$ | $S_0$\|$T_1$ | 13 | $3.6 \times 10^{10}$ | I/E |
| 20 | $CET_1$ | $S_1$\|*cis* | $S_0$\|*cis* | 14 | $1.2 \times 10^{11}$ | I/E |
| 21 | $CET_2$ | $S_1$\|*cis* | $S_0$\|$S_0$ | 14 | $4.9 \times 10^9$ | I/E |
| 22 | $OET_1$ | $S_1$\|OFF | $S_0$\|OFF | 15 | $5 \times 10^9$ | I/E |
| 23 | $OET_2$ | $S_1$\|OFF | $S_0$\|$S_0$ | 15 | $5 \times 10^5$ | I/E |

# 1.3 Continuous-time Markov chain simulation

## 1.3.1 Method description

The simulation is based on the direct method of the stochastic simulation (Gillespie) algorithm[11]. Usually, propensities $a_i(x) = k_i * x$ of species with population $x$ are determined for each transition $i$. Here, species $s \in S$ is a combination of all $n_{fl}$ fluorophores each in a specific photophysical state $p \in P$, such that $S = P_1 \times P_2 \times \ldots \times P_{n_{fl}} = \{(p_1, p_2, \ldots, p\_n_{fl}) | p_1 \in P_1, p_2 \in P_2, \ldots, p_{n_{fl}} \in P_{n_{fl}}\}$ and the size of $S$ is $|S| = |P|^{n_{fl}}$ (this is similar to Pati et al.[12]). Since each $s$ involves all $n_{fl}$, $x$ is always 1, so $a_i = k_i$. Hence, we are computationally limited to small numbers of fluorophores, ensemble simulations are not possible. On the other hand, it enables simulation of energy transfers between fluorophores at different distances simultaneously and yields accurate statistics about all photophysical states and their transitions over time.

Photophysical transitions $j \in J$ like internal conversion or fluorescent deexcitation involve the same initial and final photophysical states. Due to this ambiguity, simulating the states over time would involve subsequent statistical determination of the transitions that took place. On the other hand, simulating transitions over time contains explicit information about the photophysical states involved and hence is computationally favourable. Note that these transitions $u \in U$ transform one $s$ into another $s$, so if $n_{fl} > 1$, $U \neq J$ (see example below), however physically speaking, every $u$ is also one of $J$.

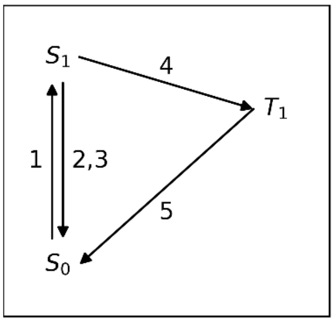


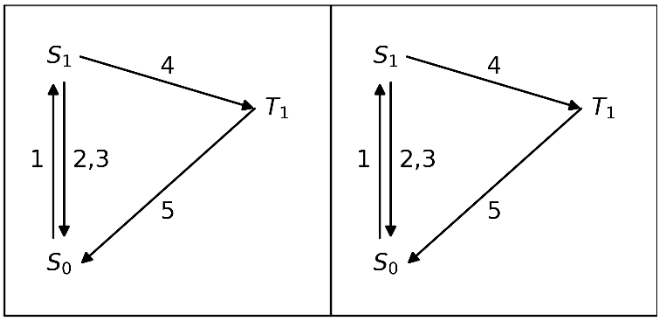


$P = \{S_0, S_1, T_1\}$ | $P = \{S_0, S_1, T_1\}$

$J = \{1, 2, 3, 4, 5\}$ | $J = \{1, 2, 3, 4, 5\}$

$S = \{S_0, S_1, T_1\}$ | $S = \{S_0|S_0, S_0|S_1, S_0|T_1, S_1|S_0, T_1|S_0, S_1|S_1, S_1|T_1, T_1|S_1, T_1|T_1\}$

$U = \{1, 2, 3, 4, 5\}$ | $U = \{1_{S_0|S_0 \rightarrow S_0|S_1}, 1_{S_0|S_0 \rightarrow S_1|S_0} 1_{S_0|S_1 \rightarrow S_1|S_1}, 1_{S_1|S_0 \rightarrow S_1|S_1}, 1_{S_0|T_1 \rightarrow S_1|T_1}, \ldots\}$

The transition matrix $T: U \times U$ contains point probabilities $p_{m,n} = \frac{k_{u_{m,n}}}{\sum_n k_{u_{m,n}}}$ with $k_{u_{m,n}} = \begin{cases} k_{u_n}, if\ s_{initial}(u_n) = s_{final}(u_m) \\ 0, otherwise \end{cases}$, that is the probability of a transition $u_n$ following the previous transition $u_m$. Hence, $T$ is nonzero, if the final species $s_{final}$ of a transition $u_m$ is the initial species $s_{initial}$ of another transition $u_n$.

Two pseudorandom numbers $0 \leq r_1, r_2 \leq 1$ are drawn. The time interval after which the next transition occurs (following transition $u_m$) is determined like $t = \frac{1}{\sum_n k_{u_{m,n}}} \ln\left(\frac{1}{r_1}\right)$, which resembles inverse transform sampling of the exponential distribution. The transition $u_n$ is chosen by looking up the first entry in $T_{m,:}$ that satisfies $\sum_{z=1}^{n} p_{m,z} > r_2$. This way, the continuous-time Markov chain simulation puts together a sequence of transitions $u$ and the

corresponding point in time. A sequence of states for each fluorophore can directly be inferred given the transition sequence. Note that simultaneous changes in the photophysical states of multiple fluorophores are only possible if the underlying photophysical transition is an energy transfer between those fluorophores.

## 1.3.2 Continuous excitation

### 1.3.2.1 Variants

The stochastic simulation algorithm is carried out step by step. This enables monitoring the processes in detail yielding the photophysical transitions, their exact time points and the corresponding photophysical state of each fluorophore. However, this comes at the expense of memory requirements (RAM). One solution for running simulations that need a lot of steps is the use of memory-maps that keep RAM usage low but increase the computation runtime. An alternative solution is to repeatedly discard all the detailed information that is unavailable in an experiment. In the most reduced approach, only the frame numbers and their photon counts are stored.

### 1.3.2.2 Predicting special cases with the limiting distribution

Predictions of photophysical statistics are possible using the limiting distribution of the Markov chain. It can be determined by using repeated matrix multiplication to solve $1\pi = \lim_{n\to\infty} T^n$, where 1 is a column vector filled with 1's and each row represents the stationary distribution $\pi$ containing the state probabilities $x_i$. The inverse of the sum of all outgoing transitions $k$ of a state resembles the mean of the exponentially distributed durations (i.e., lifetime $\tau$) of the state given that all transitions are available whenever the state is occupied, i.e., $\tau = \frac{1}{\sum k}$. The total occupation time can then be calculated as $\tau * x * n$, where $n$ is the number of steps of the Markov chain.

However, too large transition matrices introduce large errors due to floating point precision and thus making the limiting distribution difficult to obtain for systems with multiple fluorophores and transitions. Additionally, some transitions (energy transfers) are not always available which can lead to complicated mixtures of exponential distributions for state lifetimes. Therefore, predictions are only feasible for rather simple systems.

The limiting distribution needs the Markov chain to be irreducible and aperiodic. Fluorophores with the cycles $S_0$ – $S_1$ – $S_0$ and $S_0$ – $S_1$ – $T_1$ – $S_0$ have denominators 2 and 3, making the Markov chain aperiodic. If photobleaching is possible, the Markov chain is not irreducible. Modifying the transition matrix such that photobleaching is no longer available easily converts it to being irreducible. The resulting statistics are not expected to be altered due to the low probability of photobleaching. Alternatively, the fundamental matrix $N = (I - Q)^{-1}$, where $Q$ is $T$ without considering the Markovian absorbing states and $I$ is the identity matrix of $Q$, can be used to obtain the number of state visits before Markov chain absorption via $x_i = \frac{N_{j,i}}{\sum_k N_{j,k}}$. $j$ denotes the starting state, which is expected to have no significant influence due to low probabilities of photobleaching.

### 1.3.2.3 Approximation of special cases

We provide an algorithm that is very fast and that approximates a step-by-step simulation if the following requirements are met. The Markov chain must be aperiodic and irreducible, i.e., the prediction must be available. There cannot be other back- and forth-transitions between

two states except $S_0$ and $S_1$ (i.e., $S_0$ and $S_1$ are connected). Finally, all simple cycles must contain $S_0$.

The steps of the algorithm are the following:

1. Predict the relative number of occurrences of each state via $1\pi = \lim_{n\to\infty} P^n$, determine the state $s_I$ with maximum occurrences $occ_{max} = \max(\pi) * n_{steps}$ and put it into a 1-D array like $A = [s_{I,1}, s_{I,2}, \dots, s_{I,occ_{max}}]$. If $s_I$ only has one follow up state $s_{II}$, an array of alternating $s_I$, $s_{II}$ can be initialized like $A = [s_{I,1}, s_{II,1}, \dots, s_{I,occ_{max}}, s_{II,occ_{max}}]$.
2. Since $s_I$ is part of each simple cycle of the graph, it can be excluded from each cycle to convert the graph into a directed acyclic graph (DAG). This enables the determination of a topological ordering, which establishes an order that prevents visiting a state several times.
3. Select the current state $s_{current}$ based on the order (neglecting $s_I$ if $s_{II}$ already in $A$) and determine the follow up states $S_{follow}$. If $s_I \in S_{follow}$, discard it. Else, compute each probability $p_{s_{follow}} = \frac{\sum k_{s_{current} \to s_{follow}}}{\sum k_{s_{current} \to s_{any}}}$. Shuffle the indices $i$ of $s_{current}$ and put the relative number of each $s_{follow}$ according to its probability at an index $i + 1$.
4. For each state in $A$, draw a duration like $t \sim Expon(\frac{1}{\tau_{state}})$.

Since most transitions of a fluorophore are between $S_0$ and $S_1$, the algorithm can start with an alternating $S_0/S_1$ array and needs to incorporate only a handful of other photophysical states, making it computationally feasible. Whilst this can be of advantage, it is not suited for fluorophore systems where energy transfers are possible. Photobleaching cannot be considered directly, because it leads to a stationary state distribution $\pi$ that is nonzero only at the photobleached state.

### 1.3.3 Pulsed excitation

Our simulations of pulsed laser excitation are also based on the Gillespie algorithm[11] as described in 1.2.1. However, we set all excitation transition rates to zero such that the Markov chain encounters $S_0$ as an absorbing state as it does the photobleached state B. The times when laser pulses occur can be determined beforehand given the pulse intervals and the number of pulses. A probability of excitation per pulse is calculated for each fluorophore. If a fluorophore is in $S_0$ whenever a pulse occurs, a random number $r \in [0, 1]$ is compared with the assigned probability to determine a potential excitation event. The laser pulses are approximated to be instantaneous. A single laser pulse can lead to multiple fluorophores getting excited. A fluorophore can only be excited once per laser pulse.

Whenever the Markov chain is interrupted by a pulse and the system is not currently all-$S_0$, a potential future transition is remembered in case the pulse does not alter the system. Note that this potential future transition may encounter multiple pulses before being carried out. If excitation probabilities are low, the simulation can be sped up by drawing the number of pulses until next excitation from a geometric distribution. Note that the potential future transition could be discarded followed by redrawing a transition from the same conditions whenever a pulse has not led to an excitation. However, in that case it wouldn't be possible to calculate the number of pulses until the next excitation.

The $S_1$ durations are calculated as the time difference of photon detection to previous laser pulse, i.e., as done experimentally. Additionally, the fluorophore origin is not considered, which

can be advantageous to explore effects like homo-FRET on the observed fluorescence lifetime. The instrument response function and subsequent reconvolution fitting is neglected.

The probability of excitation per pulse is calculated as follows: the mean irradiance during pulse is determined as $\overline{E_{e,pulse}} = \frac{\overline{E_e}}{t_p * f_{rep}}$, where $\overline{E_e}$ is the time-averaged irradiance over one full pulse period, including both the pulse duration and the interval with no laser emission; $t_p$ is the pulse duration; $f_{rep}$ is the pulse frequency. This is used to calculate a mean excitation rate as $\overline{k_{exc}} = \sigma * \overline{\Phi}$, where $\sigma$ is the excitation cross section and $\overline{\Phi} = \frac{\overline{E_{e,pulse}}}{h\nu}$ the mean photon flux during the pulse. The probability of excitation per pulse can then be calculated using the CDF of the exponential distribution: $1 - e^{-\overline{k_{exc}} * t_p}$.

Natively, the RAM usage is low due to only keeping track of $S_1$ durations and number of detected photons per frame. This is sufficient to mimic experimental data. A detailed version exists that extends the output with the photophysical transitions, their exact time points and the corresponding photophysical state of each fluorophore as seen for the continuous excitation simulation. The pulsed excitation simulation takes about 60-fold longer than the continuous excitation simulation to simulate the same number of transitions.

### 1.3.4 Varying transition matrix

For a scenario where a constant transition matrix is not a good approximation of reality (as with varying $\kappa^2$ in Table D ID10 and ID11), we also implemented the first reaction method of the Gillespie algorithm[11]. Instead of determining the next transition time by using the sum of all transition rates and choosing the transition with a binary search in a cumulative sum of a sorted array, here all transition rates are used individually to draw an exponentially distributed sample. The minimum value of those samples represents both the next transition time and the next transition. This approach is more time-consuming for constant sets of transitions and transition rates, however if they vary throughout the simulation, the repeated sorting required for the direct method creates an overhead such that the first reaction method becomes more feasible. Generally, the duration of simulating the first reaction method is more sensitive to the total number of transitions compared to the direct method.

## 1.4 Caveats

We tried to incorporate many photophysical details in our continuous-time Markov chain simulations. However, there are still parameters that might exhibit larger variations than assumed. Simulations based on the direct method use a set of transition rates that is constant over time. Some environmental changes (including fluctuations in inter-fluorophore distances and dipole orientation factors) might still influence the transition rates more than assumed.

There are also computational limitations. We tracked the time points as 64-bit floats. As dSTORM experiments reach $10^3$ s, the minimum time step to add is around $10^{-13}$ s. Time steps drawn below this threshold are treated as instantaneous. We decided against storing the time steps instead of absolute time points because it increases RAM requirements and computational complexity.

For analyzing state statistics in multi-fluorophore systems, we reduce the system to individual single-fluorophore components. If energy transfers are available, then the following rules are applied to the analysis: (1) Transitions 18, 19, 20 and 22 (Table B) interrupt the acceptor state duration as the acceptor transitions to a higher energy state. However, we neglect this

interruption assuming that the initial acceptor state is quickly returned to. Hence, the acceptor's duration stays uninterrupted. (2) A transition duration (e.g., Fig ED), that is the time until the transition occurs starting from the previous transition of that fluorophore, is collected only from the donor's point of view if the transition is an energy transfer. (3) The duration of transitions with $S_1$ as initial state does not differentiate whether energy transfer was also an option or not. (4) An occurrence of energy transfer resembles both the transition of the donor and the acceptor.

# 2 Validation of simulation framework

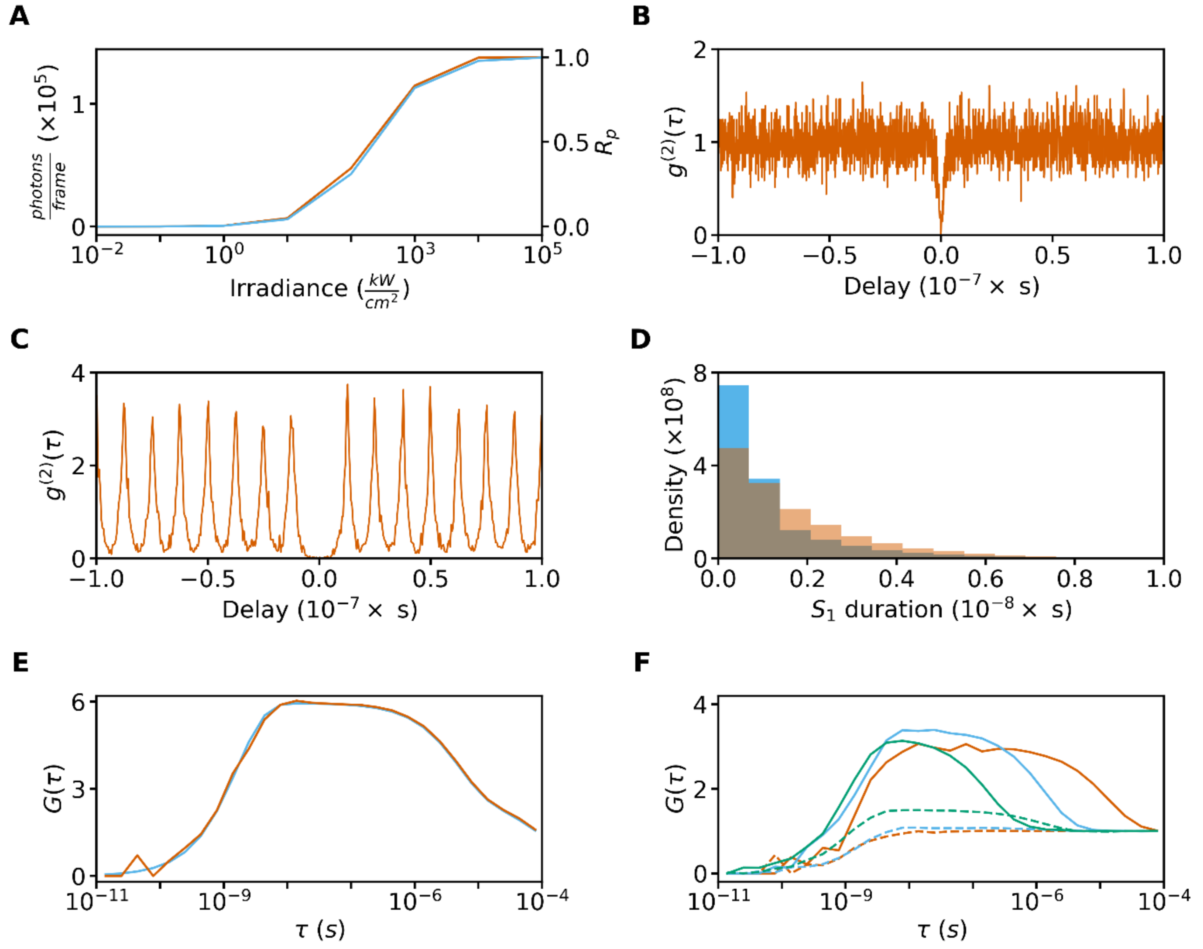


**Fig B. Validation of the simulation framework part I.** (A, B) A single-fluorophore system in continuous excitation only with states $S_0$ and $S_1$. (A) Determining the fluorescence saturation irradiance via simulation (red) and calculation (blue). (B) $g^{(2)}$ shows the antibunching dip at delay = 0 as expected for a single fluorophore. (C, D) A single-fluorophore system in pulsed excitation only with states $S_0$ and $S_1$. (C) $g^{(2)}$ shows the antibunching dip at delay = 0 and correlation peaks that correspond to the laser repetition rate. (D) Measured $S_1$ durations if the pulse interval is 12.5 ns (red) and 1 ns (blue) shows increased aliasing for 1 ns. (E, F) FCS analyses of single-Cy5 systems in continuous excitation (without dSTORM-specific conditions). (E) The simulated FCS curve (red) and the calculated FCS curve (blue) as described by Widengren et al.[2] (F) Simulated FCS curves with (solid lines) and without (dashed lines) isomerization as experimentally shown by Widengren et al.[2], red – 1 kW $cm^{-2}$, blue – 10 kW $cm^{-2}$, green – 100 kW $cm^{-2}$.

We validated our simulation framework by recreating some fundamental photophysical experiments relevant for our study.

**Single fluorophore, two-state model, continuous excitation.** The simulation of intensity as a function of irradiance returns the outcome predicted by calculating the fraction of maximum photons $R_p = \frac{k_{EXC}}{k_{EXC}+k_{S_1}}$ (Fig BA). Second order coherence of a single fluorophore reveals the expected photon antibunching dip at a delay of 0[13], given the bin resolution is sufficient (Fig BB). At larger delay times, $g^{(2)}$ fluctuates around 1 corresponding to uncorrelated photon arrivals.

**Single fluorophore, two-state model, pulsed excitation.** The second order coherence shows the expected periodic peaks corresponding to the laser repetition rate (Fig BC). $g^{(2)}(0) = 0$ can also be confirmed. In contrast to the continuous excitation, here the $S_1$ durations are determined using the previous laser pulse as starting point. Hence, increased

aliasing is expected for shorter pulse intervals, that indeed can be confirmed by simulation (Fig BD).

**Single Cy5 fluorophore, no dSTORM, FCS analysis, continuous excitation.** The simulated FCS curve aligns with the prediction as described in formula (24) in Widengren et al.[2] where prediction and simulation are based on the same photophysical model (Fig BE). Fig BF shows our simulated outcome when using a setup as described for Fig 2 in Widengren et al. Note that rate parameters for this simulation were adjusted such that they correspond to the parameter estimations of this specific reference to increase resemblance. For example, the observation of 50% *cis* and 50% *trans* relies on specific isomerization and $T_1$-associated rates that may not be met in another experimental setup. A smaller $T_1$ to $S_0$ ISC rate, as it is typical for oxygen-depleted environments, can drastically decrease *cis* fractions as $T_1$ accumulates significantly with increasing irradiance (see Table C).

**Table C. Irradiance, oxygen or thiol effect on the lifetimes of S0, cis and T1, and the rate of ISC from S1 to T1.** As irradiance increases, $T_1$ starts to accumulate – however, the effect is less if the lifetime of $T_1$ is shorter (e.g., air-saturated medium). Note that the rate of transitioning to $T_1$ is largely unaffected. Also note that in typical dSTORM experiments the medium is oxygen-depleted but in turn has increased thiol concentrations, hence the effects on $\tau_{T_1}$ may cancel each other out.

| Condition | $\boldsymbol{\tau_{S_0}}$ | $\boldsymbol{\tau_{Cis}}$ | $\boldsymbol{\tau_{T_1}}$ | $\boldsymbol{k_{ISC(S_1 \to T_1)}}$ |
|---|---|---|---|---|
| Irradiance ↑ | ↓ | ↓ | const | const |
| Oxygen ↑ | const | const | ↓ | const |
| Thiol ↑ | const | const | ↓ | const |

**Single Cy5 fluorophore, dSTORM, continuous excitation.** Gidi et al.[3] observed a linear relationship of the relaxation rate of $T_1$ and the concentration of the thiol (see their Fig S22). Our implementation confirms this relationship (Fig CA), as it is $k_{T_1}(c) = c * a * k'_{PET} + k_{ISC(T_1 \to S_0)}$, where $a$ is a constant (derived from the Henderson Hasselbalch equation), $k'_{PET}$ is the concentration-dependent PET rate with unit $s^{-1}M^{-1}$ and $c$ is the concentration of the thiol. Furthermore, they observed a linear relationship between the ON to OFF rate (i.e., the inverse ON duration) and the irradiance (see their Fig 2 h). As the irradiance mainly effects the lifetime of $S_0$ and *cis*, it can be written as $k_{ON \to OFF}(E_e) = \frac{1}{i_{S_0} * \tau_{S_0}(E_e) + i_{S_1} * \tau_{S_1} + i_{T_1} * \tau_{T_1} + i_{cis} * \tau_{cis}(E_e)}$, where $i$ is the number of visits of the respective state and is constant (also see Table C). Hence, $k_{ON \to OFF}(E_e)$ is approximately linear if $E_e$ is small, because this increases $\tau_{S_0}$ and $\tau_{cis}$ and their terms dominate. However, as $E_e$ increases, $k_{ON \to OFF}$ flattens as it mainly depends on the constants. We can confirm an approximately linear relationship for the chosen irradiance range (Fig CB).

**Multiple identical fluorophores, two-state model, no energy transfers, continuous excitation.** Without any energy transfers, the intensity emitted by fluorophores should scale linearly as a function of the number of fluorophores, which can be confirmed by simulation (Fig CC). Additionally, $g^{(2)}(0) = 1 - \frac{1}{N}$ should no longer be 0 but instead 0.75 in case of four fluorophores[14] (Fig CD).

**Multiple fluorophores, two-state model, energy transfers, continuous excitation.** In the first scenario we consider two identical fluorophores that have singlet-singlet annihilation available (Fig CE). As the rate of this transition increases, the probability for both fluorophores emitting a photon at the same time decreases[15,16], such that $\lim_{k_{SSA} \to \infty} g^{(2)}(0) = 0$. Finally, in a scenario where only the donor's emission is measured, the fluorescence intensity and $S_1$ lifetime are expected to increase as the distance between the fluorophores increases, which can be confirmed by our simulations (Fig CF).

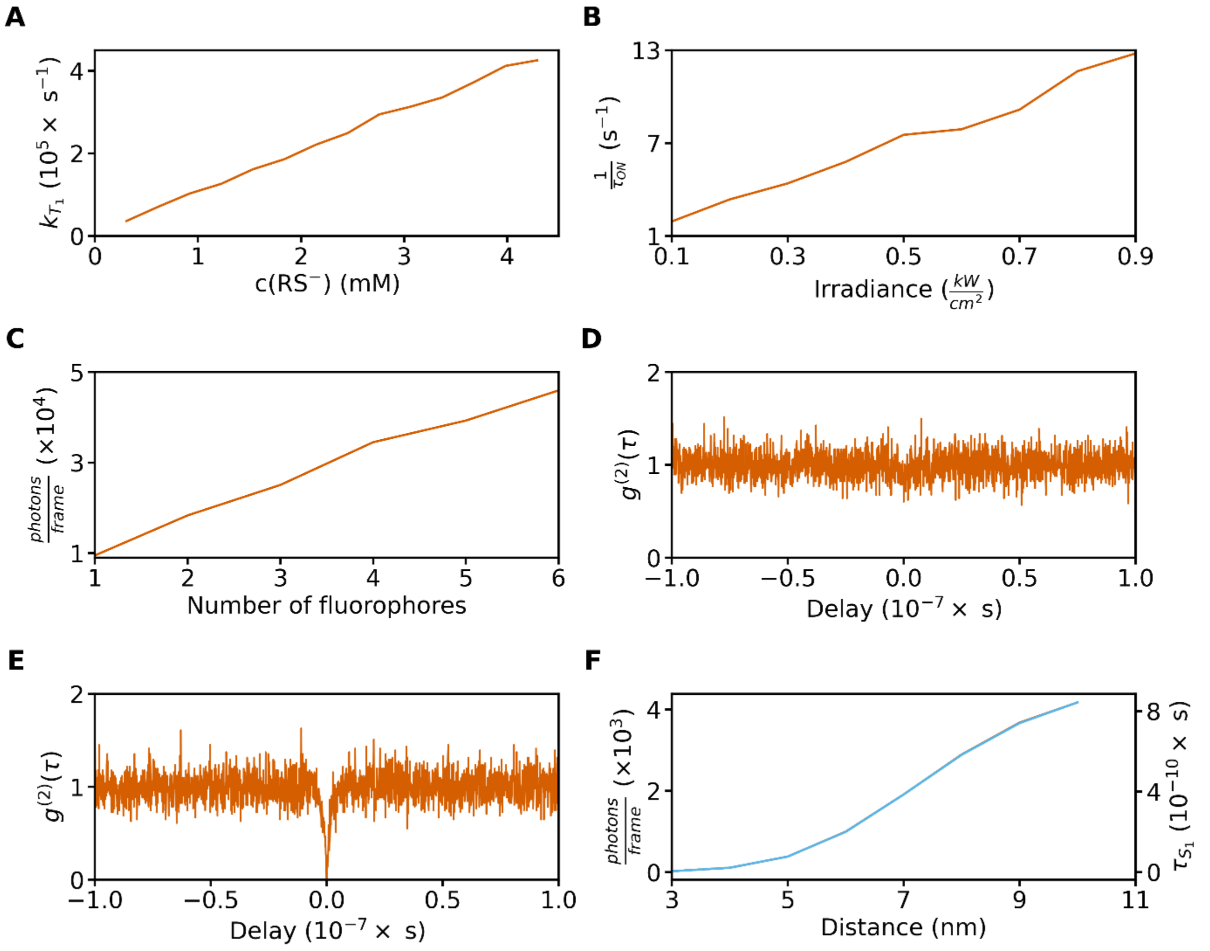


**Fig C. Validation of the simulation framework part II.** (A, B) A single-Cy5 system in continuous excitation and dSTORM-specific conditions. (A) Simulated $T_1$ relaxation rates as the concentration of thiol increases grow linearly as shown experimentally by Gidi et al.[3] (B) The inverse of the ON state lifetime increases as the irradiance increases as shown experimentally by Gidi et al.[3] (C, D) Multi-fluorophore systems (of the same kind of fluorophore) in continuous excitation only with states $S_0$ and $S_1$ and no energy transfers. (C) As the number of fluorophores contributing increases, the intensity increases additively. (D) $g^{(2)}$ shows a weakened antibunching dip at delay = 0 as expected for four fluorophores. (E, F) Multi-fluorophore systems in continuous excitation only with states $S_0$ and $S_1$, and energy transfers are available. (E) Two fluorophores of the same kind that can undergo singlet-singlet annihilation restore the antibunching dip at delay = 0. (F) Two fluorophores of different kinds that can undergo FRET, where fluorescence of only the donor is captured. As the distance increases, the intensity (red) and $S_1$ lifetime (blue) scale with the same factor.

# 3 Photophysical statistics: Effects of analysis parameters, fluorophore multiplicity, and excitation mode

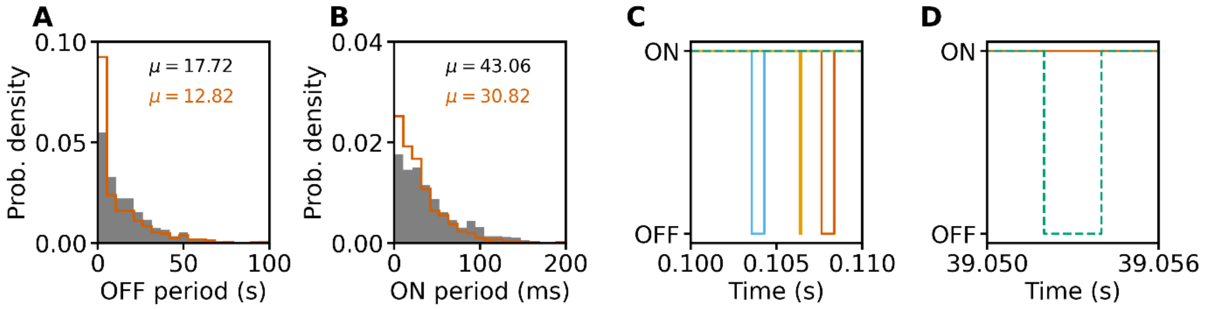


**Fig D. OFF and ON periods vs. OFF and ON states.** (A, B) OFF periods must have a duration of at least 1 (red) (see Fig 2) or 4 frames (grey). (A) The ePDF of OFF periods (consecutive frames with photon count < 10). (B) The ePDF of ON periods (consecutive frames with photon count ≥ 10). (C, D) The green dashed line shows whether an OFF or ON period is detected. The solid lines show whether the respective fluorophores are in the OFF or ON state. (C) A 3-fluorophore system (with energy transfers) showing that individual fluorophores (blue, orange, red) may be in the OFF state during an ON period. (D) A 1-fluorophore system showing that a fluorophore may be in the ON state during an OFF period.

OFF periods do not necessarily represent OFF states as shown in Fig DA, DD. Neglecting very short OFF periods (≤ 4 frames) dramatically changes the distribution of OFF periods such that it follows an exponential distribution. The exponential distribution is expected if OFF periods represent OFF states, where the OFF state has a constant relaxation rate. The population of very short OFF periods instead mainly represents transient non-emitting states such as R (as in Fig DD), $T_1$ and *cis* in combination with high temporal resolution (e.g., 1 ms frame integration time). Of course, each OFF period interrupts an ON period, such that increased temporal resolution is expected to decrease the duration of ON periods (Fig DB).

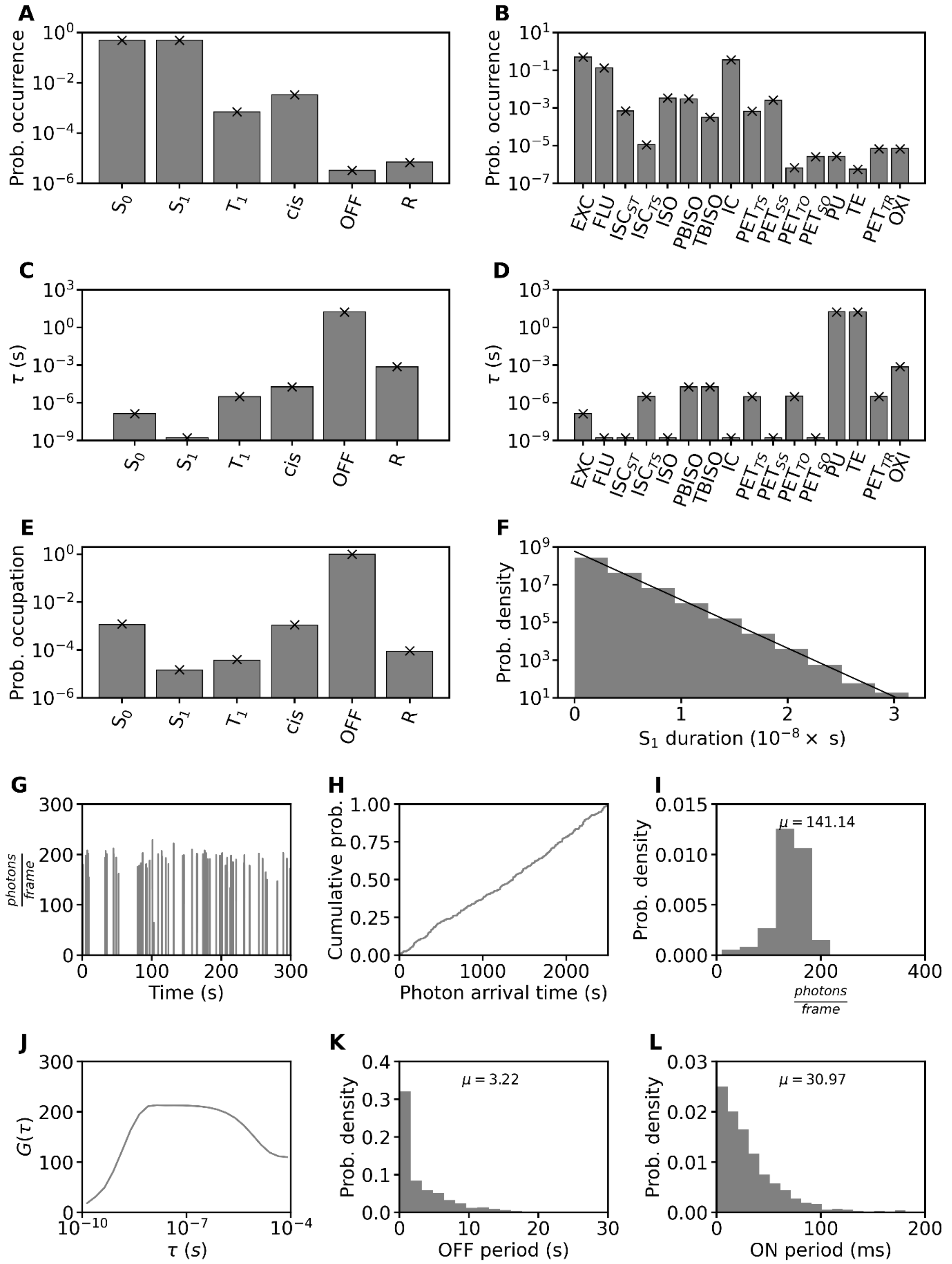


**Fig E. Photophysical statistics of four non-interacting fluorophores.** Photobleaching is disabled. (A – F) Statistics that are experimentally not available. They are predicted (crosses for A – E, line for F) using tools like the limiting distribution of the Markov chain assuming a single-fluorophore system. (A) The probability of occurrence of each photophysical state. (B) The probability of occurrence of each photophysical transition. (C) The lifetime of each photophysical state. (D) The transition lifetime of each photophysical transition. (E) The probability of occupation of each photophysical state. (F) The ePDF of durations of the photophysical state $S_1$ overlaid with an exponential decay of corresponding relaxation time. (G – L) Analyses that are experimentally available. (G) The fluorescence trajectory showing photons per frame over time. (H) The eCDF of photon arrival times. (I) The ePDF

of photon counts per frame. (J) The fluorescence correlation curve $G(\tau)$. (K) The ePDF of OFF periods (consecutive frames with photon count $< 10$). (L) The ePDF of ON periods (consecutive frames with photon count $\geq 10$).

Four spatially indistinguishable fluorophores that act independently (no energy transfers, no dimer formation) show shorter OFF periods as they are frequently interrupted by stochastically occurring ON states (Fig E). Coinciding ON states of multiple fluorophores are unlikely as seen by the unchanged ON periods compared to the single fluorophore (Fig 2).

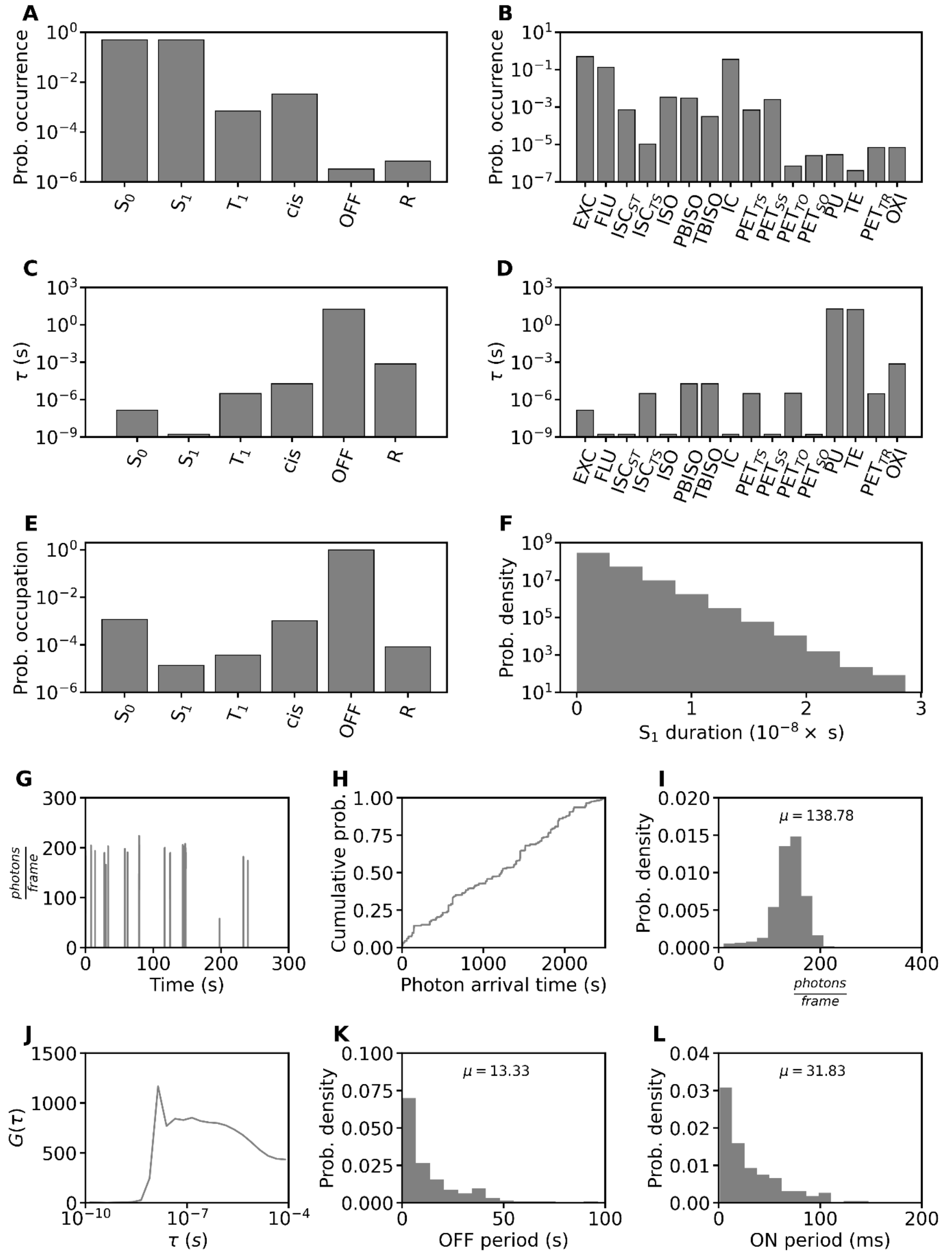


**Fig F. Photophysical statistics of a single fluorophore with pulsed excitation.** Pulse frequency is set to 80 MHz and time-averaged irradiance to 2.5 $kW\,cm^{-2}$. Pulse duration is set to 50 ps. Photobleaching is disabled. (A – F) Statistics that are experimentally not available. (A) The probability of occurrence of each photophysical state. (B) The probability of occurrence of each photophysical transition. (C) The lifetime of each photophysical state. (D) The transition lifetime of each photophysical transition. (E) The probability of occupation of each photophysical state. (F) The ePDF of durations of the photophysical state $S_1$. (G – L) Analyses that are experimentally available. (G) The fluorescence trajectory showing photons per frame over time. (H) The eCDF of photon arrival times. (I) The ePDF of photon counts per frame. (J) The fluorescence correlation curve $G(\tau)$. (K) The

ePDF of OFF periods (consecutive frames with photon count $< 10$). (L) The ePDF of ON periods (consecutive frames with photon count $\geq 10$).

Assuming all parameters other than those related to excitation remain constant between pulsed and continuous excitation, the similarity of the outcomes is primarily governed by the pulse frequency and pulse strength (i.e., excitation probability per pulse). However, even if the time averaged irradiance is equal (Fig F and Fig 2) and the pulse frequency is high, the difference in FCS analysis remains since photons are at least between-pulse-time apart.

# 4 PFA in single-fluorophore systems

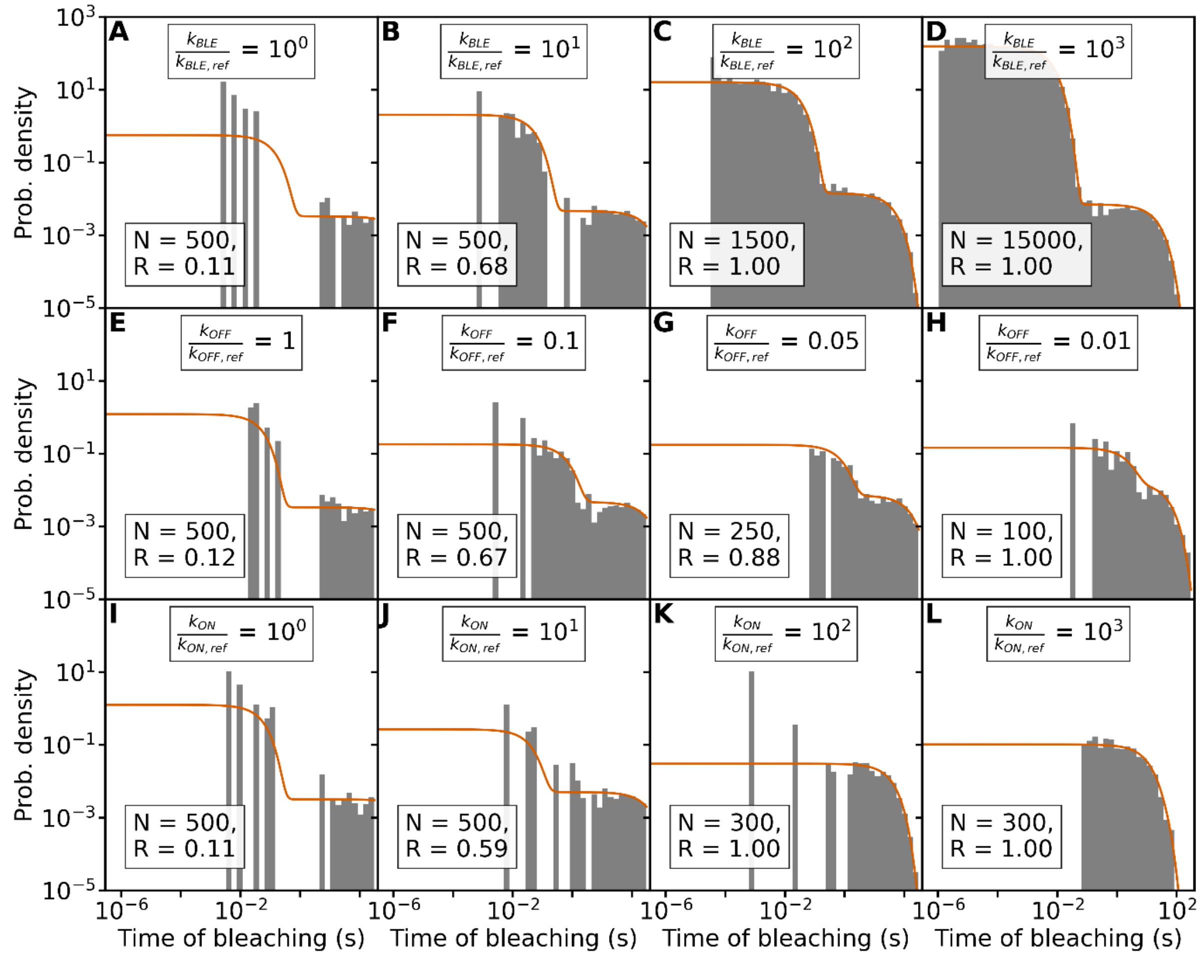


**Fig G. Global bleaching times of different single-fluorophore systems.** N is the number of simulation runs (each 300 s), R is the relative number of photobleached fluorophores. (A – D) Altering the photobleaching rate $k_{BLE}$. (E – H) Altering the rates associated with entering the OFF state ($k_{OFF} = k_{PET_{SO}} + k_{PET_{TO}}$). (I – L) Altering the rates associated with entering the ON state ($k_{ON} = k_{PU} + k_{TE}$). The analytical expression (red solid lines) and fitting procedure are described in SI section 8.

Global bleaching times of single fluorophores can be approximated with a two-exponential mixture distribution, where the parameters are estimated using the corresponding PFAs (Fig G). The global bleaching times are directly influenced by the different alterations ($k_{BLE}$, $k_{OFF}$, $k_{ON}$) introduced in the fluorophore systems and consequently dictate the shape of PFAs. It also demonstrates the distributions of photon arrival times and global bleaching times to be approximately equal in simulated single-fluorophore systems.

Increasing the rate of photobleaching $k_{BLE}$ reduces the total number of photons emitted during the measurement period (Fig HA) and before photobleaching (as the time of photobleaching decreases, see Fig GA – GD). At moderate changes, the ON period histogram remains unaffected as only one ON period per fluorophore gets interrupted by photobleaching (Fig HB). As the chance to photobleach per ON period is approaching 1, the number of uninterrupted ON periods is approaching 0 and the shift to shorter ON periods becomes more visible. The OFF periods are not affected (Fig HC).

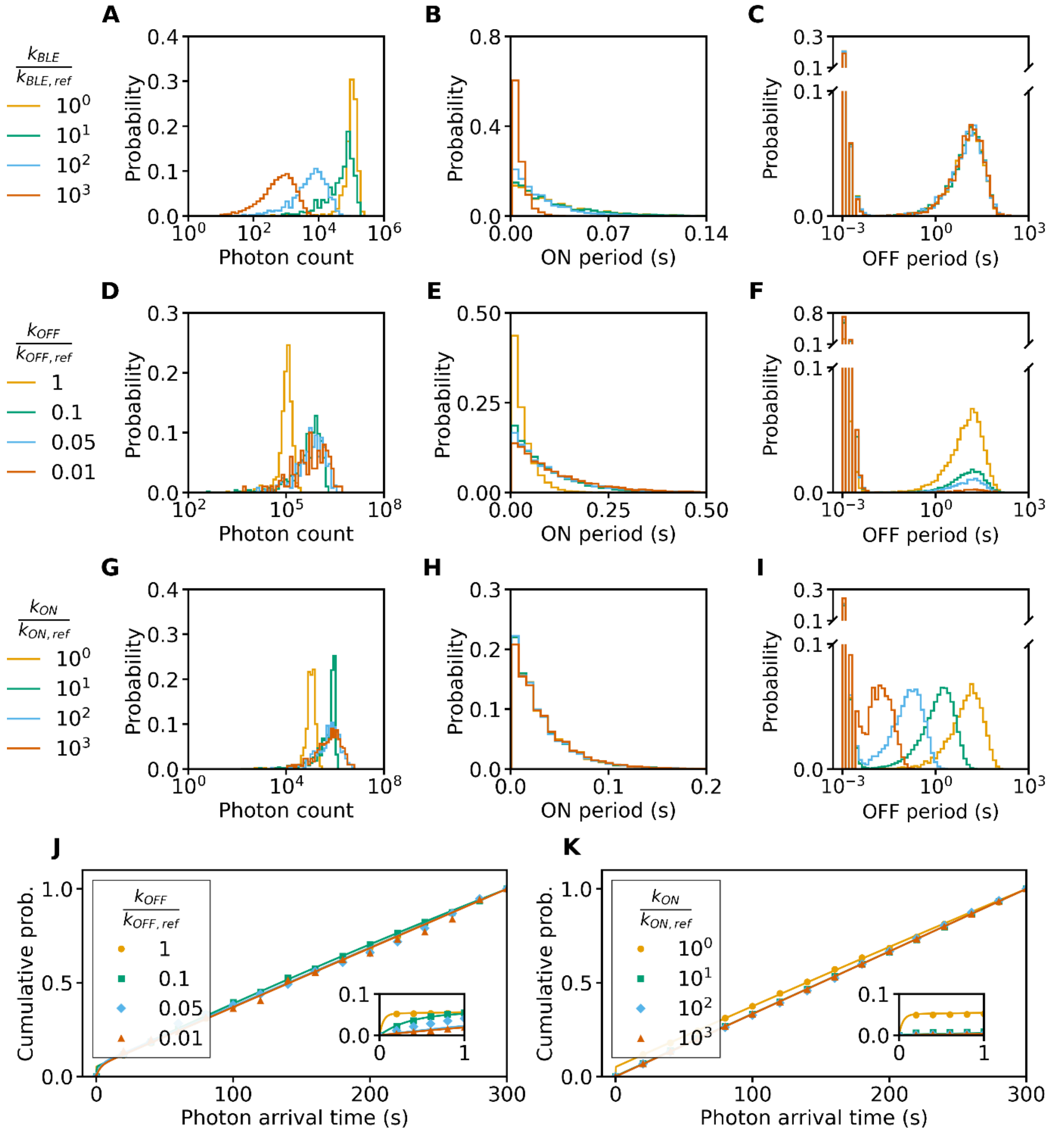


**Fig H. Extended analysis of Fig 3: Parameters that control the shape of PFAs of single fluorophores.** (A – C) Altering the photobleaching rate $k_{BLE}$. (D – F) Altering the rates associated with entering the OFF state ($k_{OFF} = k_{PET_{SO}} + k_{PET_{TO}}$). (G – I) Altering the rates associated with entering the ON state ($k_{ON} = k_{PU} + k_{TE}$). (A, D, G) Probability histogram of total photon counts during 300 s. (B, E, H) Probability histogram of ON periods. (C, F, I) Probability histogram of OFF periods. (J, K) The eCDF of photon arrival times of datasets (D – F) and (G – I) but without photobleaching. The markers depict simulated results. The analytical expression (solid lines) and fitting procedure are described in SI section 8.

Lowering the ON to OFF rate $k_{OFF}$ increases the probability of photobleaching during the duration of the measurement such that it approaches 1 and hence, the total number of photons emitted approaches the maximum (i.e., the number of photons emitted before photobleaching) (Fig HD). The ON periods approach the maximum which is limited by the probability of short OFF periods that do not represent the OFF state (Fig HE). Predictably, the distribution of OFF periods shifts to the left as the probability of long-lasting OFF states diminishes (Fig HF). It should be noted that decreasing $k_{OFF}$, i.e., $k_{PET_{TO}}$ and $k_{PET_{SO}}$, also increases the chance of photobleaching per excitation cycle as the transitions compete with $ISC_{ST}$ and BLE. However, the effect is negligible as the rates are small compared to other competitors. Hence, lowering

$k_{OFF}$ can be treated as increasing the chance of photobleaching per ON state by increasing the number of excitation cycles per ON state.

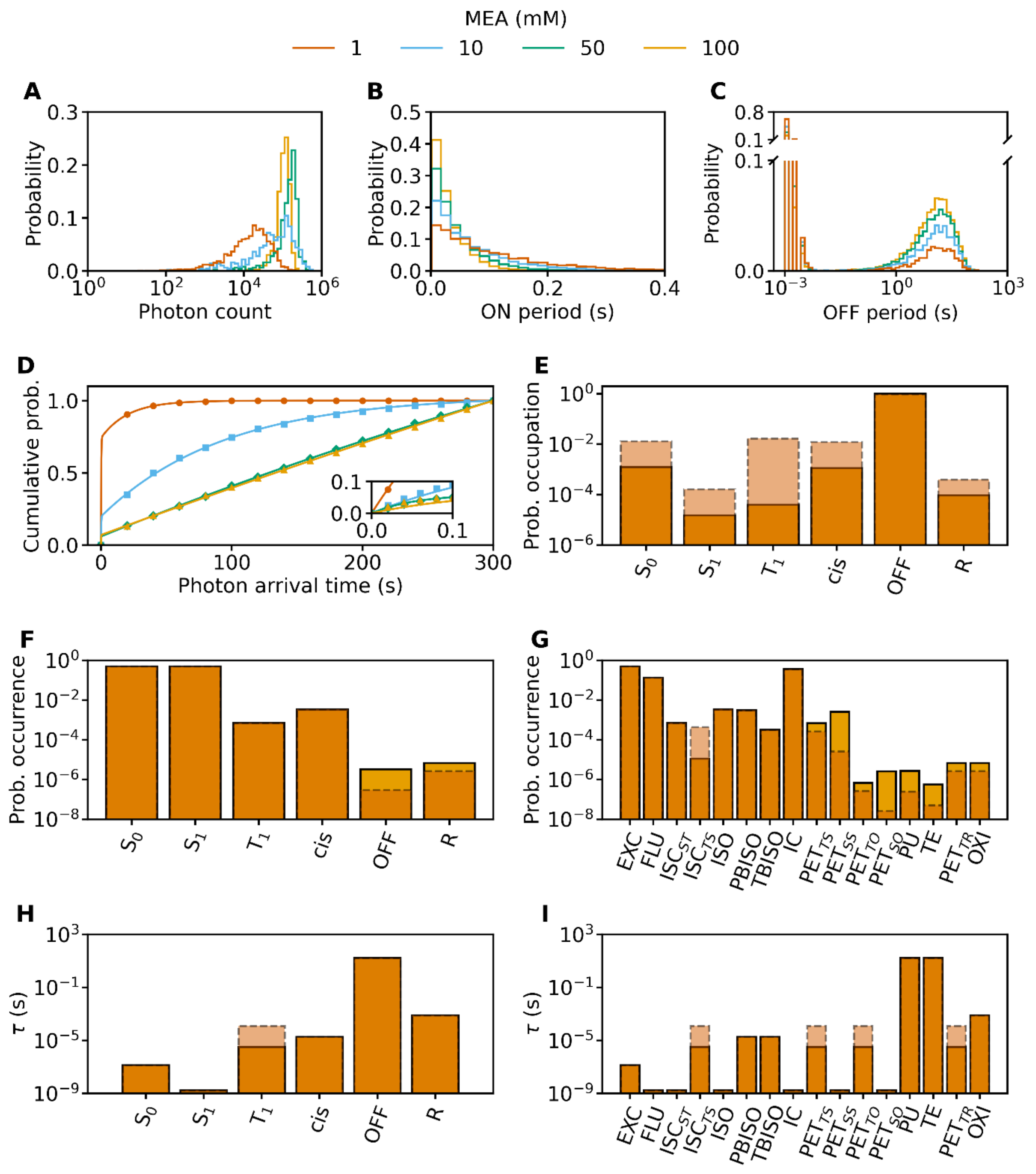


**Fig I. Effects of alterations in thiol concentrations.** The concentration of mercaptoethylamine (MEA) of 100 mM corresponds to thiol-mediated PET rates given in Table B, 50 mM to 0.5-fold, 10 mM to 0.1-fold and 1 mM to 0.01-fold. (A – D) 100 mM, 50 mM and 10 mM are simulated 500 times, 1 mM is simulated 2500 times. (A) Probability histogram of total photon counts during 300 s. (B) Probability histogram of ON periods. (C) Probability histogram of OFF periods. (D) The eCDF of photon arrival times. The markers depict simulated results. The analytical expression (solid lines) and fitting procedure are described in SI section 8. (E – I) Predicted photophysical statistics with 100 mM MEA (orange, solid) and 1 mM MEA (transparent red, dashed) that are not experimentally available. Photobleaching is disabled. (E) The probability of occupation of each photophysical state. (F) The probability of occurrence of each photophysical state. (G) The probability of occurrence of each photophysical transition. (H) The lifetime of each photophysical state. (I) The transition lifetime of each photophysical transition.

As the OFF state's duration is getting decreased by increasing the OFF to ON rate $k_{ON}$, the probability of photobleaching during the duration of the measurement approaches 1 like the

effect described for lowering $k_{OFF}$ (Fig HG). The ON periods are not affected (Fig HH). Unsurprisingly, the OFF periods get shorter as the rate to return to the ON state increases (Fig HI).

Fig HJ and HK show that without photobleaching, the PFAs are not altered despite changing the rates $k_{OFF}$ and $k_{ON}$, further indicating the connection of the shape of PFAs to global bleaching times.

In Fig 3 and H we show how the rate of entering the OFF state $k_{OFF} = k_{PET_{SO}} + k_{PET_{TO}}$ controls the shape of PFAs. While we can easily adjust the specific rate values in simulations, changes in $k_{OFF}$ in experiments are accompanied by alterations in all thiol-mediated PET rates, dictated by the concentration of the thiol[3]. Here we discuss the expected outcome of lowering $k_{OFF}$ by lowering $[A^-]$.

The probability of all dSTORM-specific transitions is decreased (Fig IG), where PU, TE and OXI are indirectly effected by the lower probability of occurrence of OFF and R (Fig IF). The probability of ISC from $T_1$ to $S_0$ is increased (Fig IG) as competing transitions get less likely. Accordingly, the probability of photobleaching (from $T_1$) is also increased. This is not shown as photobleaching can only occur once and is therefore difficult to be statistically evaluated. The lifetime of $T_1$ is increased as $PET_{TS}$ is the major triplet relaxation pathway at high thiol concentrations and in an oxygen-depleted environment (Fig IH). $PET_{SS}$ does not have a significant impact on $S_1$ lifetime as rates of IC and FLU are two orders of magnitude higher even at increased thiol concentrations. Interestingly, while OFF is $\frac{1}{11}$ as likely to occur in 1 mM MEA compared to 100 mM MEA, R is only at $\frac{1}{2.5}$ (Fig IF) due to OFF being formed via both $S_1$ and $T_1$ whereas R being formed only via $T_1$.

Therefore, lowering $[A^-]$ has a multifaceted impact on the global bleaching rate, it decreases $k_{OFF}$ and decreases photostability by increasing the probability of BLE per $T_1$. This is reflected in PFA by a stronger deviation from linearity at same $k_{OFF}$ values (see Fig 3 for comparison). Accordingly, the total number of photons emitted during the measurement period (Fig IA) and before photobleaching is reduced. At moderate PET rate changes, the reduced photostability is countered by the increased number of fluorophores that reach the time of photobleaching within the measurement duration. The ON periods approach the maximum which is limited by the probability of short OFF periods that do not represent the OFF state (Fig IB). Note that the increased $T_1$ lifetime at lower $[A^-]$ increases the probability of short OFF periods. On the other hand, the probability of long OFF periods invoked by OFF states decreases, leading to a shift to the left in the OFF period distribution (Fig IC). However, the probability of OFF states is still higher compared to only altering $k_{OFF}$ (Fig HF) since $PET_{SO}$ and $PET_{TO}$ must compete with $PET_{SS}$ and $PET_{TS}$.

# 5 PFA in multi-fluorophore systems – disproving alternatives to OET

In the following, we discuss energy transfer pathways that may initially appear as an alternative explanation for the experimentally observed PFAs. Note that we want to consider them isolated from other hypotheses and hence do not include OET in the set of transitions.

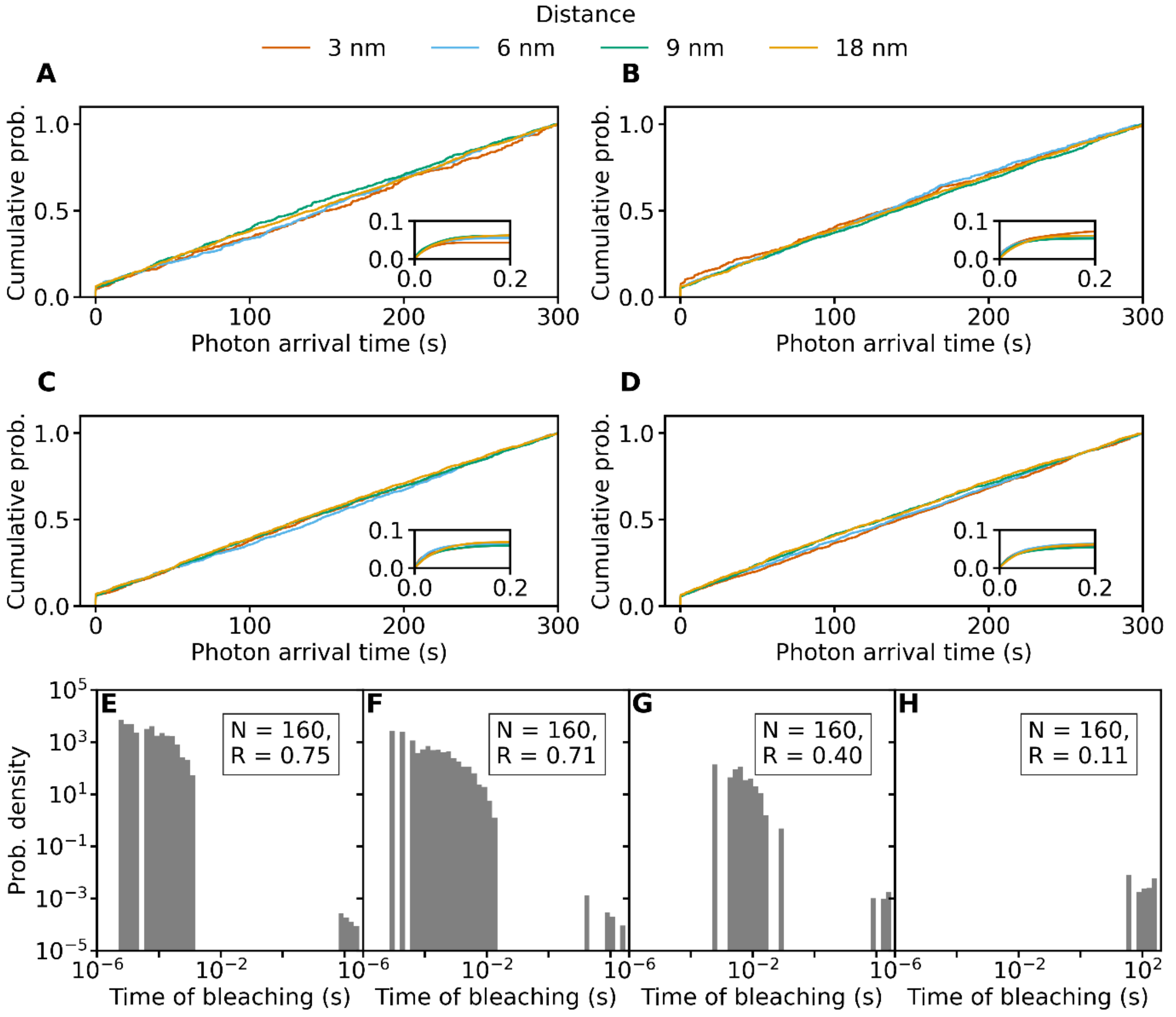


**Fig J. PFA of 4-fluorophore systems that yield photoionized fluorophores following STA.** The following photoionization efficiencies are simulated: 1% (A, E), 0.1% (B, F), 0.01% (C, G), 0% (D, H). (A – D) The eCDF of photon arrival times. (E – H) Global bleaching times for 3 nm distances. N is the number of fluorophores simulated (40 runs × 4 fluorophores, each run 300 s), R is the relative number of photobleached fluorophores.

Suppose STA to provide enough energy for Cy5 to get photoionized leading to the radical cation $Cy5^{+\cdot}$. By the same hypothetical reasoning, SSA could also induce electron ejection, however due to the low probability of coinciding $S_1$ states, we neglect this pathway. $Cy5^{+\cdot}$ is expected to have a high likelihood to undergo photobleaching. Hence, it resembles an energy transfer mechanism that may increase the global bleaching rate. Whether it does, depends on the probability of STA leading to $Cy5^{+\cdot}$ and subsequently $Cy5^{+\cdot}$ to B as it can also be viewed as a competitor to $T_1$-photobleaching.

Our demonstration assumes an instant conversion of $Cy5^{+\cdot}$ to B and a 100% efficiency, i.e., we neglect the reduction of $Cy5^{+\cdot}$ returning it back to $S_0$. The efficiency of STA leading to photoionization is set to 1% (Fig JA, JE), 0.1% (Fig JB, JF), 0.01% (Fig JC, JG) or 0%

(Fig JD, JH). It becomes clear that even though the global bleaching rate is increased (Fig JE – JH), the decreased total number of photons emitted by the affected fluorophores contribute only little to the cumulative sum (Fig JA – JD). Additionally, STA requires donor and acceptor to be in the ON state, a condition that is only certain in the beginning of a measurement and else unlikely. We can therefore discard the idea of energy transfer-induced photobleaching to be the underlying mechanism of inter-fluorophore distance-dependent PFA shapes.

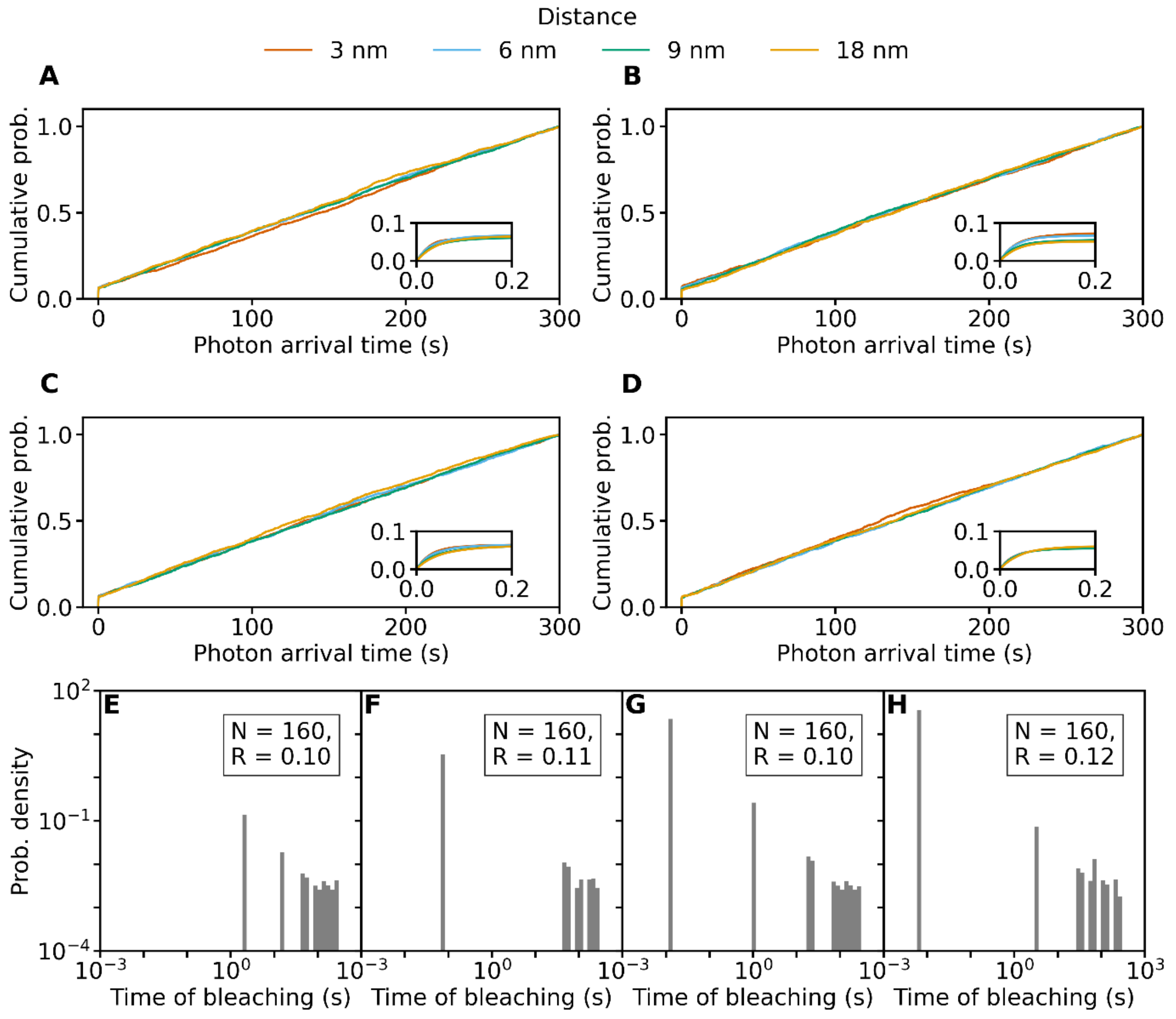


**Fig K. PFA of 4-fluorophore systems undergoing RISC following STA.** The following RISC efficiencies are simulated: 100% (A, E), 10% (B, F), 1% (C, G), 0.1% (D, H). For 0%, see Fig JD, JH. (A – D) The eCDF of photon arrival times. (E – H) Global bleaching times for 3 nm distances. N is the number of fluorophores simulated (40 runs × 4 fluorophores, each run 300 s), R is the relative number of photobleached fluorophores.

Given that $T_2$ (invoked by STA) is of higher energy than $S_1$ ($T_1$ at 1.35 eV[17] + $T_1$ absorption), RISC can have significant efficiency converting $T_2$ to $S_1$. Hence, STA can then quench the $T_1$ state (to be precise, STA always quenches the $T_1$ state, however given the short lifetime of $T_2$, $T_1$ is quickly restored if not considering RISC) and can therefore lower the OFF state probability. We demonstrate the effects using efficiencies of 100% (Fig KA, KE), 10% (Fig KB, KF), 1% (Fig KC, KG) and 0.1% (Fig KD, KH). RISC only prevents one of the two pathways that invoke the OFF state, since both $T_1$ and $S_1$ can serve as initial states. Additionally, it competes with $T_1$-photobleaching, potentially decreasing the global bleaching rate (Fig KE – KH). It should be noted that our simulations simplify reality by only including $T_1$ as initial state of photobleaching. Still, even if the global bleaching rate was increased by STA/RISC due to a high $S_1$-photobleaching probability, the issue with the lowered contribution

to the total photon count remains. Finally, as soon as a fluorophore enters the OFF state it can no longer serve as a donor nor an acceptor, limiting the time for energy transfer to a short range in the beginning (like STA + photoionization, Fig J). We can therefore rule out energy transfer-mediated decrease in OFF state probability as the underlying mechanism of inter-fluorophore distance-dependent PFA shapes.

# 6 Multi-fluorophore systems exhibiting OET

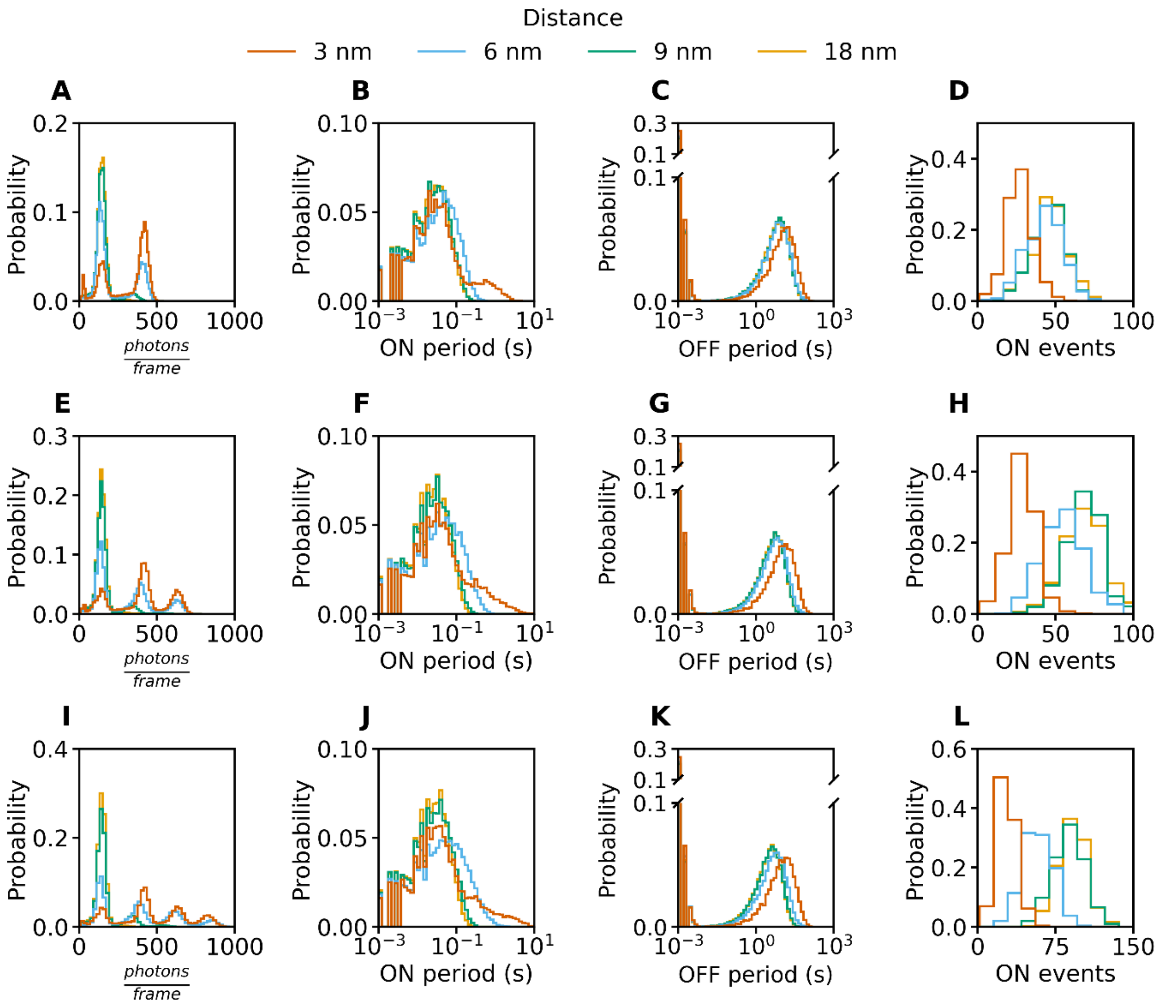


**Fig L. Extended analysis of Fig 4 part I: Statistics of multi-fluorophore systems undergoing OET.** (A – D) Two fluorophores, (E – H) three fluorophores, (I – L) four fluorophores as shown in Fig 4. (A, E, I) Probability histogram of photon counts per frame. (B, F, J) Probability histogram of ON periods. (C, G, K) Probability histogram of OFF periods. (D, H, L) Probability histogram of number of ON periods, i.e., ON events.

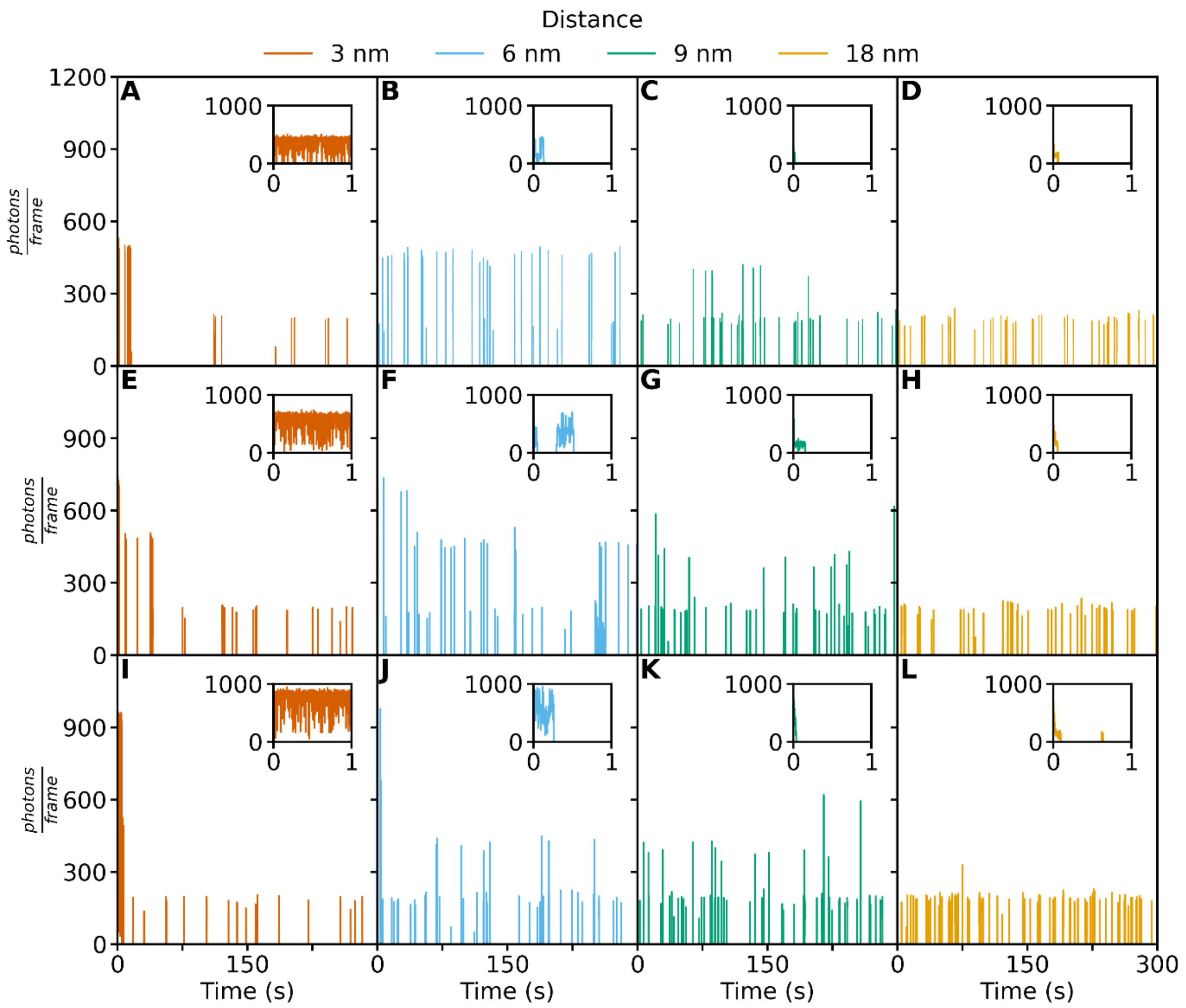


**Fig M. Extended analysis of Fig 4 part II: Fluorescence time traces of multi-fluorophore systems undergoing OET.** (A – D) Two fluorophores, (E – H) three fluorophores, (I – L) four fluorophores. The insets show the time interval from 0 to 1 s.

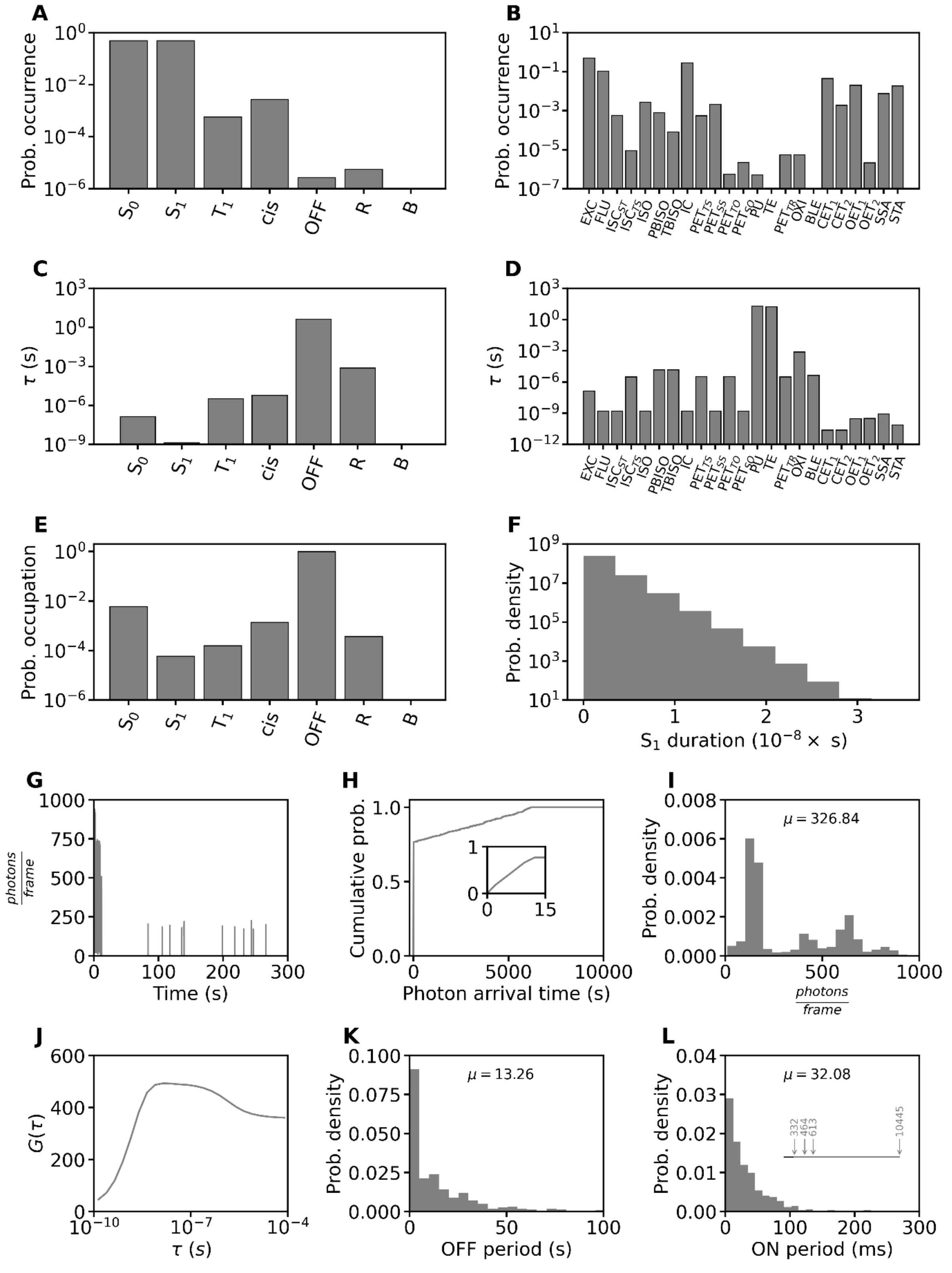


**Fig N. Photophysical statistics of four fluorophores at 3 nm distance with energy transfers enabled.** The energy transfers include SSA, STA, CET and OET. (A – F) Statistics that are experimentally not available. (A) The probability of occurrence of each photophysical state. (B) The probability of occurrence of each photophysical transition. Values of energy transfers represent 3 nm and 4.24 nm summarized. (C) The lifetime of each photophysical state. (D) The transition lifetime of each photophysical transition. Values of energy transfers represent 3 nm and 4.24 nm summarized. (E) The probability of occupation of each photophysical state. (F) The ePDF of durations of the photophysical state $S_1$. (G – L) Analyses that are experimentally available. (G) The fluorescence trajectory showing photons per frame over time. (H) The eCDF of photon arrival times. (I) The ePDF of photon counts per frame. (J) The fluorescence correlation curve $G(\tau)$. (K) The ePDF of OFF periods (consecutive frames

with photon count < 10). (L) The ePDF of ON periods (consecutive frames with photon count ≥ 10). The inset shows a logarithmic timescale with outliers that are excluded from the histogram and its mean.

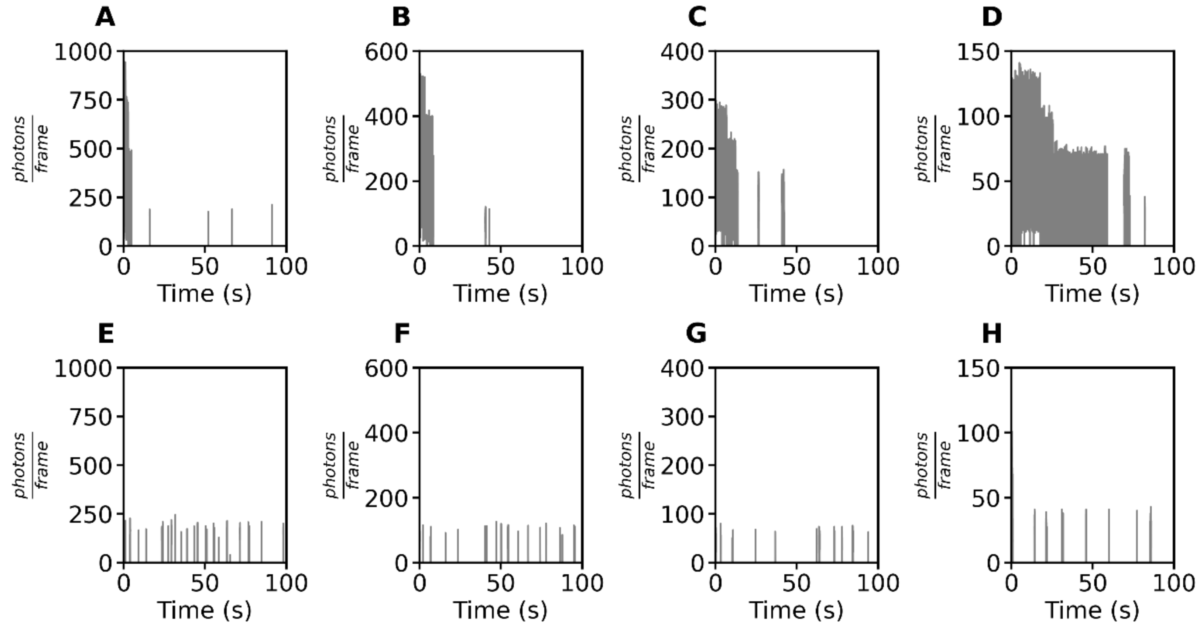


**Fig O. Effects of different irradiances on fluorescence time traces.** Four fluorophores are simulated at 3 nm distance. (A – D) OET enabled. (E – H) OET disabled. (A, E) 100%, i.e., 2.5 kW $cm^{-2}$; (B, F) 50%; (C, G) 25%; (D, H) 10%.

Fig O can be compared to extended data Fig 5 of Helmerich et al.[10] where lower irradiances (50%, 25%) slow down the short-distance-induced accelerated blinking kinetics and decrease fluorescence intensity, both of which can be confirmed by simulation. Additionally, disabling OET does not show the accelerated blinking and hence, decreased irradiances only result in a decrease in fluorescence intensity and an increase in OFF state lifetime (i.e., the ‘standard’ blinking is slowed down).

# 7 Multi-fluorophore systems exhibiting OET and FRET to the radical anion

We successfully recreated PFAs with simulations based on the proposed photophysical model including the energy transfer between ON and OFF. However, other results and analyses were not captured with the same accuracy. In the following we discuss these discrepancies and explore potential explanations. Minor differences, such as generally higher fluctuations in the experimental data, are not examined further. These fluctuations likely arise from transition rates being sensitive to small environmental changes.

First, we discuss the effective absorption cross section for photoinduced uncaging of the OFF state at 640 nm, where OET requires a minimum value larger than reported by Gidi et al.[3] We proceed with discussing fluorescence lifetimes in more detail than in the main article. The discrepancy of fluorescence lifetimes of simulation and experiment brought us to the conclusion that there must be another energy transfer available in the photophysical model. The radical anion R as acceptor is an exclusive candidate. We then examine second order coherence results, where simulations of four fluorophores at 6 nm distance produced too large $g^2(0)$ values. Again, the previously identified energy transfer to R is the most likely solution to the discrepancy. Lastly, we show that including the energy transfer to R decreases fluorescence intensities and increases short OFF periods at 3 nm distance, further improving agreement with experimental data. We show that distance-dependent shapes of PFA are maintained in the altered photophysical model, confirming energy transfer with R as acceptor to be compatible with our other findings.

## 7.1 Effective absorption cross section of OET

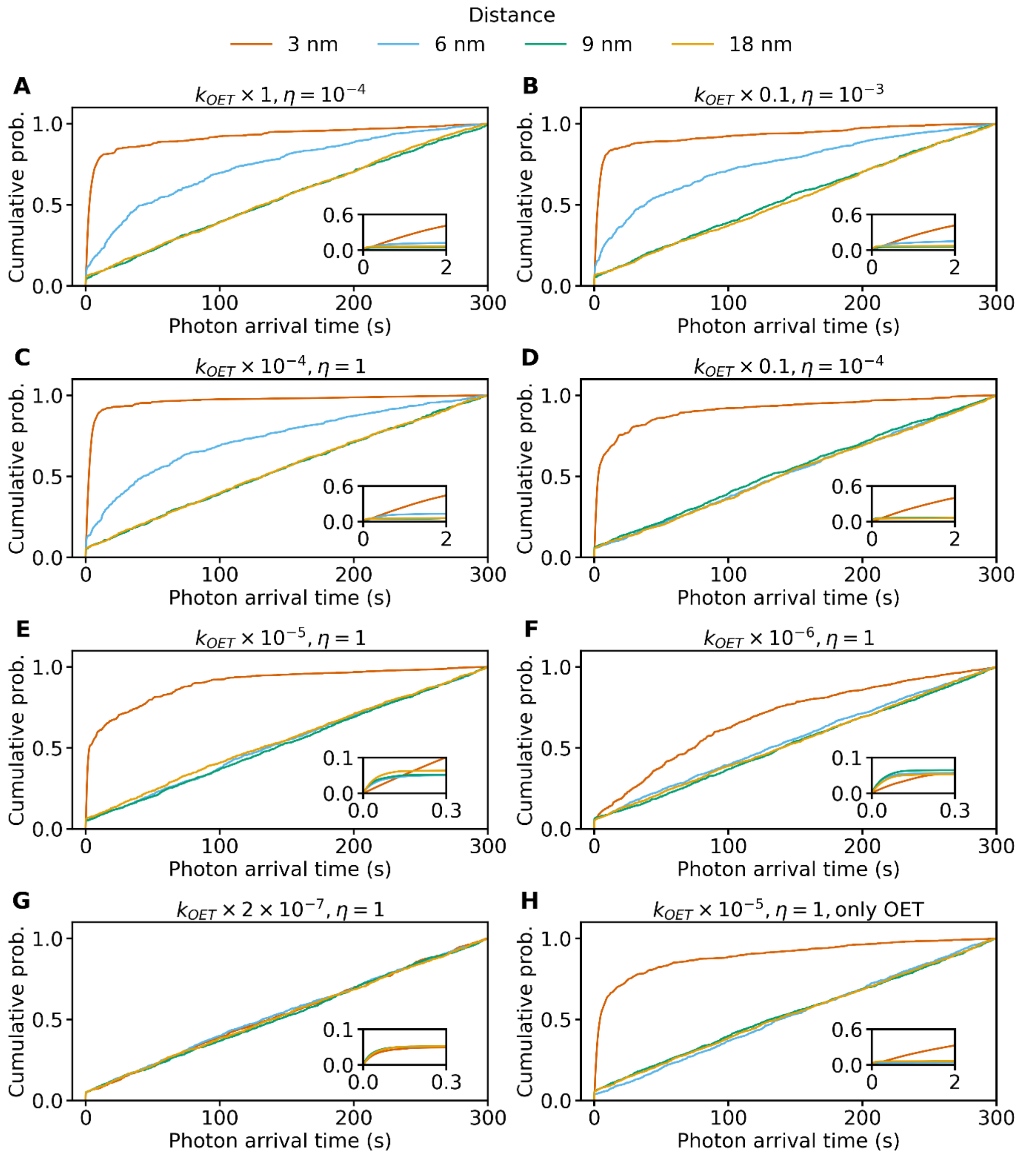


**Fig P. PFA of 4-fluorophore systems exhibiting OET in different variations.** Each condition is simulated 40 times. Here, $k_{OET}$ denotes the sum of $k_{OET_1}$ and $k_{OET_2}$ and is multiplied with a factor $f$, $\eta$ denotes the efficiency of $k_{OET_2}$, i.e., $\frac{k_{OET_2}}{k_{OET}}$. (A) $f = 1, \eta = 10^{-4}$, (B) $f = 0.1, \eta = 10^{-3}$, (C) $f = 10^{-4}, \eta = 1$, (D) $f = 0.1, \eta = 10^{-4}$, (E) $f = 10^{-5}, \eta = 1$, (F) $f = 10^{-6}, \eta = 1$, (G) $f = 2 * 10^{-7}, \eta = 1$, (H) $f = 10^{-5}, \eta = 1$, no energy transfers other than OET enabled.

As mentioned in Table A, we used an effective absorption cross section for photoinduced uncaging of $\sigma_{PU,640\,nm} = 6 * 10^{-24} cm^2$ as derived from Gidi et al[3]. For OET transitions we estimated a rate constant of $k_{OET} = 4 * 10^9 s^{-1}$ at 3 nm distance using the absorption spectrum of the OFF state provided by Gidi et al. We then needed an efficiency of around 0.01% for OET to induce a transition of OFF to $S_0$ (Fig PA). Considering the shape of the absorption spectrum, the efficiency corresponds to an absorption cross section of $\sigma_{PU,640\,nm} = 3 * 10^{-21} cm^2$, i.e.,

500-fold higher than the one mentioned above. The effective absorption cross section is simply $\sigma_{PU,640\,nm} = \eta_{PU} * \sigma_{640\,nm}$, such that Fig PA, PB and PC show the same $\sigma_{PU,640\,nm}$. The strongest effect of OFF state rescue can be observed with $\eta_{PU} = 1$, so we tested lowering $\sigma_{PU,640\,nm}$ using this efficiency (Fig PE – PG). Already at one order of magnitude lower, we lose any signatures for 6 nm (Fig PE) and when arriving at $\sigma_{PU,640\,nm} = 6 * 10^{-24} cm^2$, even that of 3 nm. Interestingly, removing energy transfers other than OET as potential competitors does not have a significant impact (Fig PE, PH). We conclude that we cannot lower the efficiency of OET without lowering the ON to OFF probabilities.

An absorption cross section of $3 * 10^{-21} cm^2$ is very small compared to absorption cross sections of standard energy transfer acceptors (e.g., $\sigma_{640\,nm}$ of Cy5 $S_0$ is $2.7 * 10^5$-fold), which suggests that our result is within a comparable regime. Nevertheless, to explore possible solutions to the discrepancy we need to be aware that we rely on the smaller value ($6 * 10^{-24} cm^2$) to explain long OFF states using excitation light of 640 nm. Still, the energy transfer considers the absorption across a range of wavelengths such that a red-shifted OFF state absorption in our experimental conditions can account for both the needed minimum $\eta_{PU}$ and the smaller value of $\sigma_{PU,640\,nm}$. More precisely, the red shift could be explained by any of the following three factors:

(1) Gidi et al. reported a second OFF state which had the tendency to show smaller lifetimes and yields, however there were significant differences between different experimental conditions. Considering that we use MEA instead of β-ME on top of all the other differences to their experimental setup it may well be the case that their minor population is our major population. They reported a red-shifted absorption for the second OFF state, consistent with our requirements.

(2) On the same note, the general differences between experimental conditions may cause differences in the shape of the absorption spectrum – such as a red-shifted absorption of the MEA-mediated OFF state.

(3) The linking of Cy5 to DNA and their immobilization via streptavidin-biotin can lead to a more rigid fluorophore. We may have a red shift in absorption due to an increase in planarity and a narrower absorption due to a decrease in vibrational broadening. It should be noted that Gidi et al. determined $\sigma_{PU,640\,nm}$ using immobilized Cy5-ssDNA and the absorption spectrum using free Cy5.

The requirements for a small $\sigma_{PU,640\,nm}$ imposed by the literature also provides a lower bound for the photobleaching rate. Decreasing the photobleaching rate leads to more linear PFA. Although this could be compensated by a higher $OET_2$ rate, restoring an apparently correct PFA, such an increase would require a larger effective absorption cross section $\sigma_{PU,640\,nm}$, thereby increasing the discrepancy with literature values. Consequently, experimental PFA provide constraints for both the OFF rescue efficiency and the photobleaching rate.

## 7.2 Fluorescence lifetimes

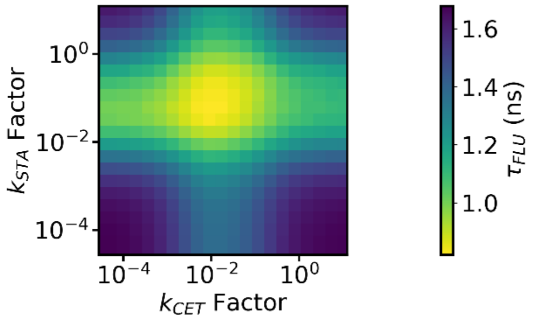


**Fig Q. Rate constants of STA and CET and corresponding fluorescence lifetimes.** The lowest possible fluorescence lifetime in conditions without dSTORM-specific transitions, given that the lifetime of $T_1$ and *cis* are not altered and that the energy transfers STA and CET have 100% acceptor state recycling, is 0.81 ns. It is achieved with 0.1 × $k_{STA}$ and 0.01 × $k_{CET}$.

**Table D. Fluorescence lifetimes in different simulated conditions.** The fluorescence lifetimes are recorded using only $S_1$ durations that deexcite via fluorescence. Each condition is simulated 10 times for $10^8$ steps. N is the number of fluorophores, the distance is set to 3 nm, photobleaching is disabled. The adjustment numbers represent different changes to the photophysical model, either qualitatively or quantitatively: 1 – no CET; 2 – $k_{CET}$ × 0.1; 3 – $\eta_{CET_2}$ is 0, meaning that no CET promotes the *cis* to *trans* transition; 4 – $k_{CET}$ × 0.05 and $\eta_{CET_2}$ is 0.005; 5 – no STA; 6 – $\kappa^2$ is distributed according to the static $\kappa^2$ distribution for energy transfers that have a rate constant > $10^9$; 7 – $k_{OET}$ × 0.1 and $\eta_{OET_2}$ is 0.001; 8 – $k_{OET}$ × 10 and $\eta_{OET_2}$ is 1; 9 – $k_{CET}$ × 0.01 and $\eta_{CET_2}$ is 0, $k_{STA}$ × 0.1, i.e., CET and STA are set such that their impact on fluorescence lifetime is maximized (see Fig Q); 10 – energy transfer to R ($k_{3nm} = 10^9 s^{-1}$), where 10.1 only considers $S_1|R$ to $S_0|R$, 10.2 also considers $S_1|R$ to $S_0|S_0$ with efficiency $10^{-4}$ and 10.3 with efficiency $10^{-3}$; 11 – $k_{PET_{TR}}$ × 10; 12 – $k_{PET_{TR}}$ × 100.

| ID | N | Irradiance (kW cm$^{-2}$) | dSTORM | Adjustment | Energy transfer | $\bar{\tau}_{FLU}$ $(10^{-9}s)$ | $\sigma_{\tau_{FLU}}$ $(10^{-13}s)$ |
|---|---|---|---|---|---|---|---|
| 1 | 4 | 2.5 | - | - | - | 1.70 | 4.5 |
| 2 | 4 | 2.5 | - | - | + | 1.31 | 37.2 |
| 3 | 4 | 0.01 | - | - | + | 1.70 | 5.5 |
| 4 | 4 | 2.5 | - | 1 | + | 1.35 | 45.1 |
| 5 | 4 | 2.5 | - | 2 | + | 1.30 | 47.3 |
| 6 | 4 | 2.5 | - | 3 | + | 1.29 | 37.6 |
| 7 | 4 | 2.5 | - | 4 | + | 1.18 | 34.7 |
| 8 | 4 | 2.5 | - | 4, 5 | + | 1.55 | 6.7 |
| 9 | 4 | 0.01 | - | 4, 5 | + | 1.68 | 4.5 |
| 10 | 4 | 2.5 | - | 6 | + | 1.25 | 28.6 |
| 11 | 4 | 2.5 | - | 4, 6 | + | 1.20 | 27.7 |
| 12 | 4 | 2.5 | + | - | - | 1.69 | 4.9 |
| 13 | 4 | 2.5 | + | - | + | 1.63 | 19.6 |
| 14 | 4 | 2.5 | + | 7 | + | 1.65 | 6.5 |
| 15 | 4 | 2.5 | + | 8 | + | 1.65 | 2.0 |
| 16 | 4 | 2.5 | + | 4 | + | 1.44 | 10.7 |
| 17 | 4 | 2.5 | + | 9 | + | 1.17 | 14.4 |
| 18 | 4 | 2.5 | + | 10.1, 11 | + | 1.14 | 47.5 |
| 19 | 4 | 2.5 | + | 10.1, 12 | + | 0.72 | 37.0 |
| 20 | 3 | 2.5 | + | 10.1, 12 | + | 0.92 | 32.5 |
| 21 | 2 | 2.5 | + | 10.1, 12 | + | 1.07 | 41.2 |
| 22 | 4 | 2.5 | + | 10.2, 12 | + | 0.81 | 32.9 |
| 23 | 4 | 2.5 | + | 10.3, 12 | + | 1.24 | 39.8 |

Experimentally measured 4-fluorophore systems with 3 nm inter-fluorophore distance showed significantly lowered fluorescence lifetimes compared to a reference fluorophore. Furthermore, photoswitching buffer and trolox buffer behaved differently (Fig 2 g of Helmerich et al.[10]). The system initially consisting of four fluorophores showed increasing fluorescence lifetimes as photobleaching progressed (Fig 2 j of Helmerich et al.[10]).

Our simulations show only a small decrease of fluorescence lifetimes (Table D ID13) for four fluorophores with 3 nm inter-fluorophore distance compared to a reference (ID12). Therefore, we explored possible adjustments to the photophysical model that could lead to fluorescence lifetime signatures as observed experimentally.

Without taking dSTORM-specific transitions into account, simulations show much shorter $\tau_{FLU}$ (ID2) compared to a reference (ID1). At first, one may expect a similar $\tau_{FLU}$ in dSTORM conditions, if the energy transfer-mediated rescue of OFF is efficient enough. However, even when setting the rate of OET 10-fold higher and with a 100% conversion rate of OFF to $S_0$ (i.e., shortening the OFF state lifetime from s to µs), $\tau_{FLU}$ is still much larger (ID15). This is because the $T_1$ lifetime in dSTORM conditions is much smaller ($10^{-5}$ s instead of $10^{-3}$ s, this assumes oxygen scavenging role of MEA to be mimicked), and we implemented STA without considering RISC, hence STA can happen much more often when mimicking buffer conditions that contain no MEA. The lifetime of *cis* is not directly affected by MEA, however the energy transfer at 3 nm has a rate high enough to successfully compete with virtually any fluorescence, which is why it does not make much difference whether to include CET or not (ID4 vs ID2). Adjusting either the rate of CET or the efficiency of CET promoting the *cis* to *trans* transition does not make much difference (ID5, ID6). Adjusting both can increase the impact of CET on fluorescence lifetimes with (ID16) and without dSTORM (ID7). Note that both CET and STA-driven fluorescence lifetime signatures are irradiance dependent (ID3, ID8, ID9) as irradiance dictates the number of $S_1$ states during the acceptor state lifetime. While the lifetime of *cis* is increased at lower irradiances, thermal back-isomerization limits such an increase, confirming experimental observations (Fig S10 of Helmerich et al.[10]).

We can further decrease $\tau_{FLU}$ in dSTORM conditions by setting both CET and STA rates low enough to allow for fluorescence to occur and high enough to make an impact on the fluorescence lifetime, and by enabling 100% acceptor state recycling for both transitions (ID17). Adjusting OET itself does not work (ID14) as a minimum OFF rescue rate is required which either limits the number of energy transfers (e.g., every 1.000 – 10.000 OET transition, the acceptor state is converted to $S_0$) or the rate of OET is too high to allow for fluorescence.

The initial parameter values were selected based on established literature. The proposed adjustments were made solely to explore the model's behavior and to guide the simulation towards specific outcomes of interest. These adjustments are not supported by empirical evidence and should therefore be interpreted with caution. Additionally, even with the adjustments to OET, STA and CET, simulated $\tau_{FLU}$ in dSTORM conditions do not get below 1 ns. Experimental data shows $\tau_{FLU}$ of as low as 0.66 ns. We therefore need energy transfers that are available for a lot of the time while also having a rate that is in a reasonable range (balance of allowing for fluorescence and making an impact on fluorescence lifetimes). A good candidate is the radical anion R as acceptor: it has a long lifetime ($10^{-4}$ – $10^{-3}$ s) and the absorption of R in regions of Cy5 emission can be expected to be low, allowing for a rate of around $10^9$ $s^{-1}$ at distances of 3 nm. Indeed, pairing this energy transfer with an increase of the transition rate of $T_1$ to R (supported by Srambickal et al.[5]) can lead to $\tau_{FLU}$ signatures like those observed experimentally (ID18, ID19). For three and two fluorophores, an expected increase of $\tau_{FLU}$ can be observed (ID20, ID21). Incorporation of energy transfer-mediated rescue of R lowers the impact on $\tau_{FLU}$ depending on the rescue efficiency (ID22, ID23). However, the

efficiency used for ID22 still allows for enough energy transfer to push $\tau_{FLU}$ well below 1 ns, and it is the same efficiency as assumed to be the case for OFF rescue.

Another solution to bring simulated results closer to experimentally observed $\tau_{FLU}$ may be a different dipole orientation factor $\kappa^2$. Experimentally, we found that fluorophores are randomly oriented (circular and linear polarized excitation light made no difference), which points towards an unrestricted rotation regime. However, for $\kappa^2$ to be equal to the dynamic isotropic limit (as generally assumed in this study), the rotational diffusion must be fast compared to the fluorescence lifetime. Since the rates of CET and STA at 3 nm distance are large, even fast rotational diffusion may not be enough to visit enough orientation configurations in time t << τ. Hence, a time-independent $\langle\kappa^2\rangle = \frac{2}{3}$ may not be justified. Additionally, the linking of Cy5 to DNA and their immobilization via streptavidin-biotin is known to increase the fluorescence lifetime of Cy5 (e.g., from 1 ns to 1.7 ns) due to a decrease of non-radiative deexcitation, indicating a slower rotational speed compared to free Cy5. The intermediate rotational speed regime is difficult to simulate due to the cyclic dependence of $\kappa^2$ and τ. We therefore focus only on the static regime, which we assume to be the case for all FRET transitions that have a certain minimum rate. In each simulation step (see SI section 1.3.4) the rates of selected energy transfers, which are given for $\langle\kappa^2\rangle = \frac{2}{3}$, are multiplied with $\frac{3\kappa^2}{2}$, where $\kappa^2$ is drawn from the static $\kappa^2$ distribution. It is assumed that between such transitions, enough time for a new random orientation has passed. ID10 and ID11 show that the influence in the given simulation conditions is relatively low, indicating that it is an unlikely candidate to be responsible for too large $\tau_{FLU}$ in our simulations.

# 7.3 Second order coherences

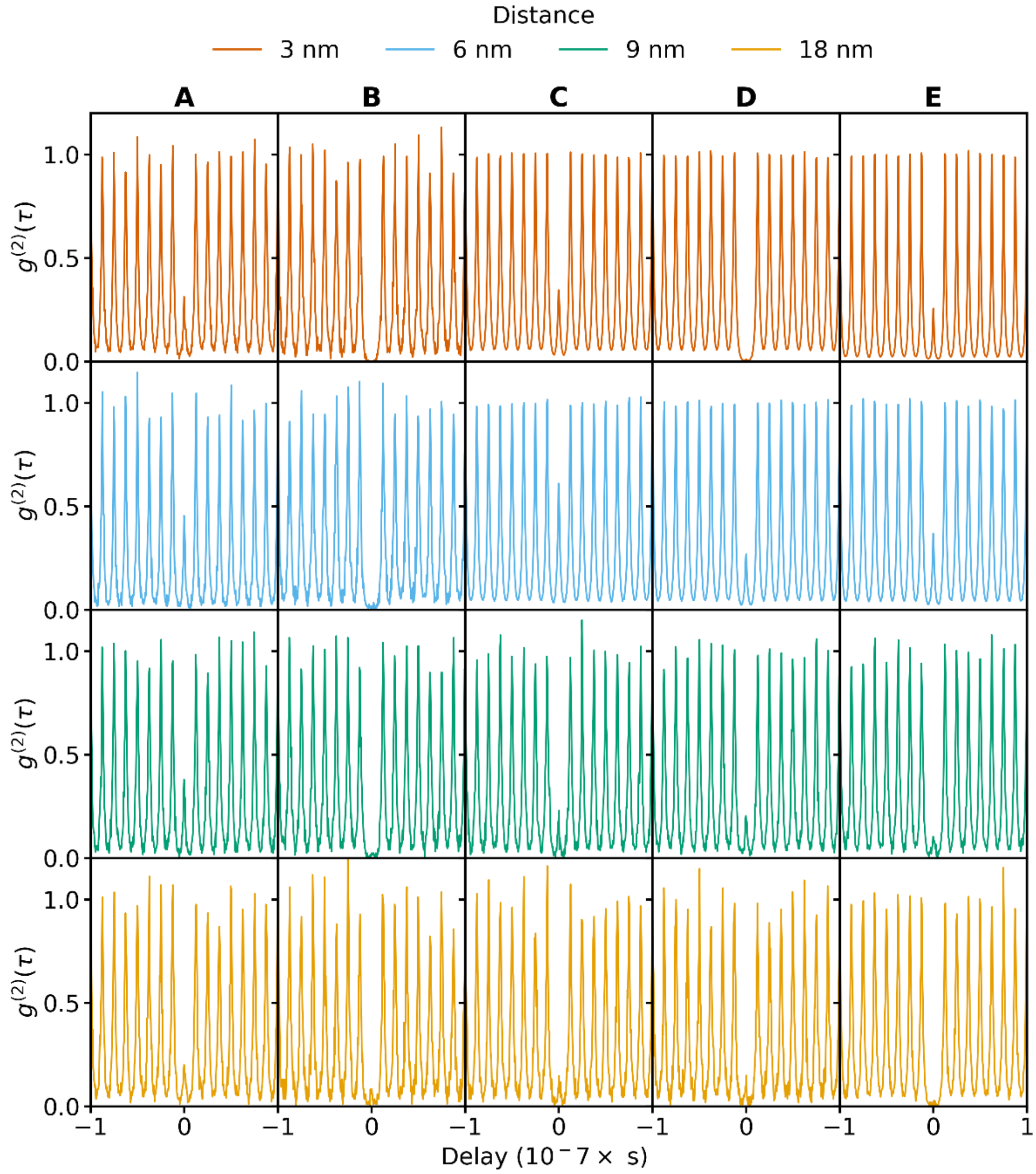


**Fig R. Second-order coherence of four fluorophores in different simulated conditions.** Pulse frequency is set to 80 MHz and time-averaged irradiance to 2.5 kW cm$^{-2}$. Pulse duration is set to 50 ps. The first 20 ms of the total 50 s are discarded. (A) Without MEA, $k_{SSA} \times 0.01$. (B) Without OET, $k_{SSA} \times 0.01$. (C) $k_{SSA} \times 0.01$. (D) Standard conditions. (E) $k_{SSA} \times 0.01$, $k_{PET_{TR}}$ × 100, energy transfer to R ($k_{3nm} = 10^9 s^{-1}$). Corresponding fluorescence time traces are displayed in Fig S.

$g^2(0)$ reveals how likely it is to observe multiple photons at the same time. The probability depends on many factors, as visible in Fig R. For example, coinciding ON states, as mediated by OET, increases the probability. However, energy transfers and non-emitting photophysical states lower the probability. Especially SSA can significantly decrease the probability of finding multiple photons at the same time, as it depletes one of two coinciding $S_1$ states. Therefore, when analyzing the degree of antibunching of fluorophores at different distances, it must be considered that at higher distances, the probability of OET is lower, but the probability of SSA is also lower. It is also important to know what time interval of a measurement is being analyzed: in the very beginning, all fluorophores are in the ON state, so even high distance configurations that have a vanishingly small chance to rescue OFF states via OET can still show diminished photon antibunching. Later, fluorophores may have already photobleached, decreasing OFF state rescue and maximum $g^2(0)$.

Photon antibunching can be used to adjust the rate of SSA, for which estimates are rarely available due to the short $S_1$ lifetime. For example, Fig RD shows that with our standard estimate of the SSA rate constant, complete photon antibunching is observed at 3 nm distance. Experiments show different results (Fig S8 of Helmerich et al.[10]), so we adjusted our estimate (Fig RC).

Our photophysical model does not necessarily lead to lower $g^2(0)$ values at higher distances as it is the case in experimental data (Extended Data Fig 4 of Helmerich et al.[10]). For example, at 6 nm, we still observe significant OET-mediated OFF state rescue (and therefore, PFA signatures). Considering the impact of positive and negative feedback loops of OET (that is, many coinciding ON states can stabilize through a higher OFF state rescue probability), we can either expect many or no coinciding ON states. If we consider that the SSA rate is lower than at 3 nm, the only explanation for smaller $g^2(0)$ at 6 nm is an energy transfer like mechanism that rescues non-emitting photophysical states other than the OFF state. Such an energy transfer could rescue states more efficiently at shorter distances, possibly compensating for the increased SSA probability. $CET_2$, which has a higher rate at 3 nm leading to a higher probability of converting non-emitting *cis* to emitting *trans* is not a good candidate because it is still large enough at 6 nm to outcompete other $S_1$-outgoing transitions like IC. Therefore, another rescue (e.g., $T_1$, R) would be required to explain the data. Note that as photobleaching progresses, fluorophores at 3 nm may still be able to rescue OFF efficiently while 6 nm no longer can, leading to more ON periods consisting of only a single fluorophore in the case of 6 nm. This effect may also contribute to experimentally observed lower $g^2(0)$ values, where fluorescence time traces of 300 instead of 50 s were analyzed.

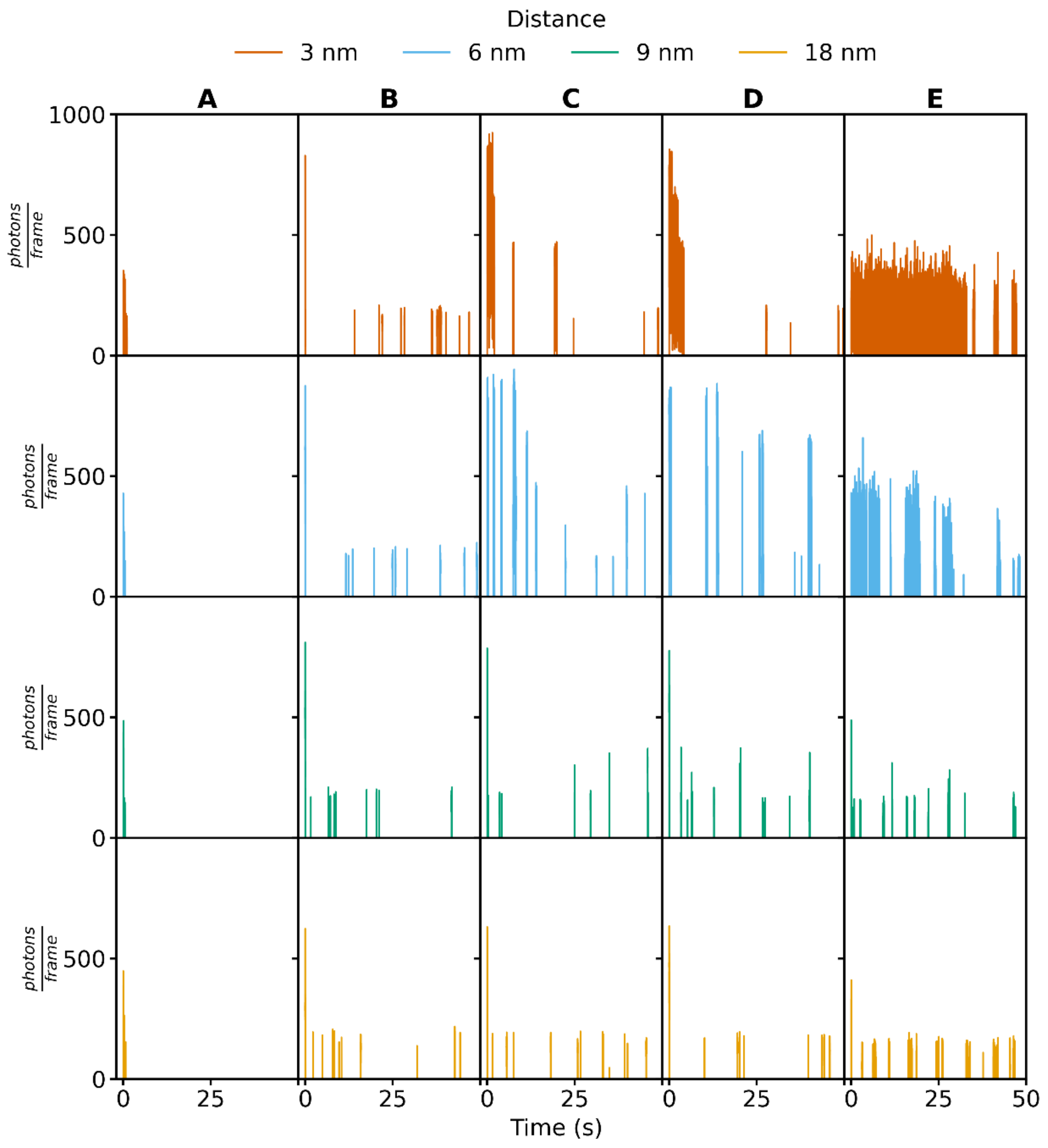


**Fig S. Fluorescence time traces of four fluorophores in different simulated conditions.** Pulse frequency is set to 80 MHz and time-averaged irradiance to 2.5 kW cm$^{-2}$. Pulse duration is set to 50 ps. (A) Without MEA, $k_{SSA} \times 0.01$. (B) Without OET, $k_{SSA} \times 0.01$. (C) $k_{SSA} \times 0.01$. (D) Standard conditions. (E) $k_{SSA} \times 0.01$, $k_{PET_{TR}}$ × 100, energy transfer to R ($k_{3nm} = 10^9 s^{-1}$). Corresponding second-order coherences are displayed in Fig R.

## 7.4 Adjusted photophysical model

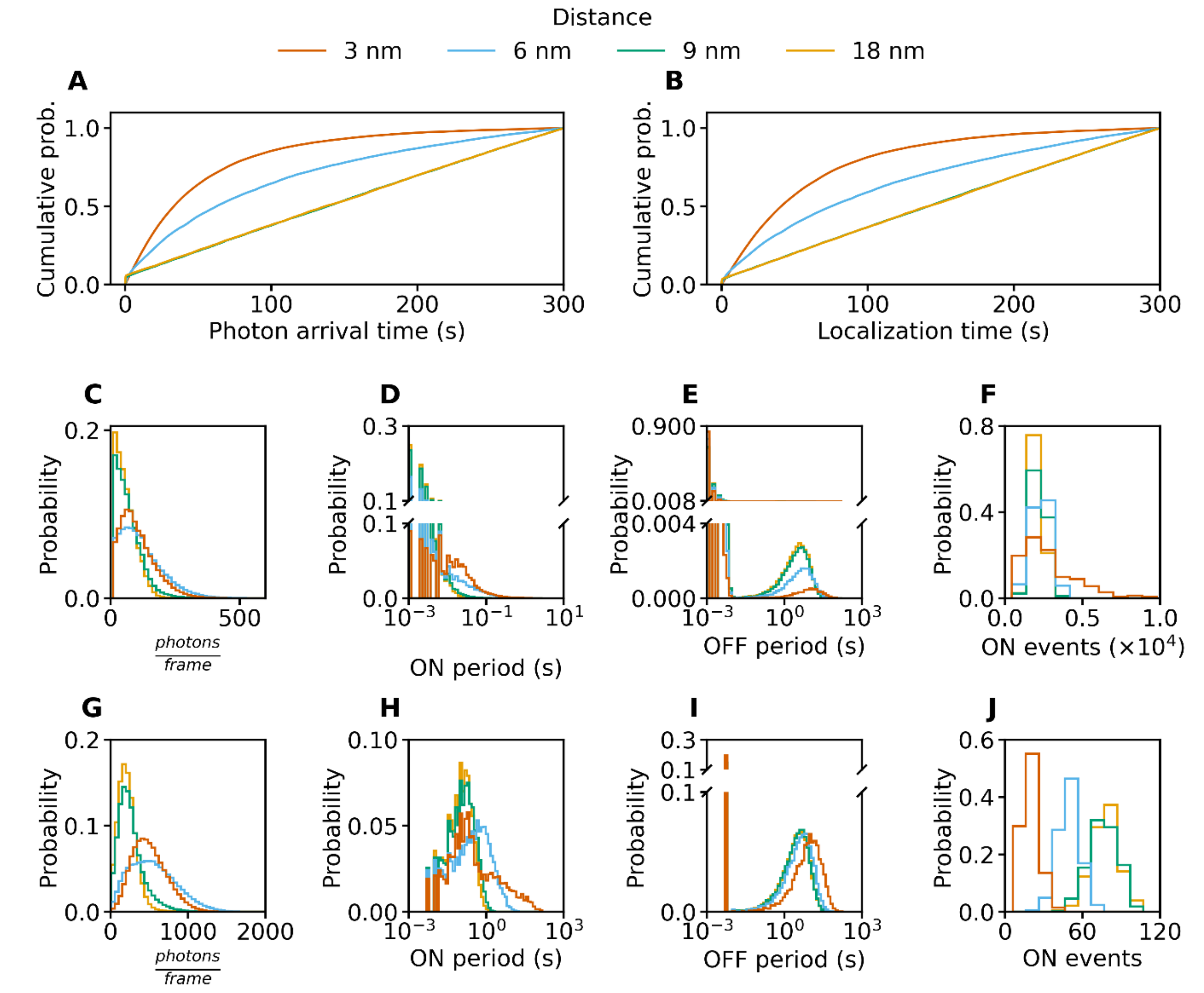


**Fig T. PFA and statistics of 4-fluorophore systems exhibiting OET and energy transfer to R.** Adjustments to photophysical model: $k_{PET_{TR}}$ × 100, energy transfer to R ($k_{3nm} = 10^9 s^{-1}$). Each distance is simulated 325 times. (A) The eCDF of photon arrival times. (B) The eCDF of localization times. (C – F) Frame integration time is set to 1 ms, (G – J) frame integration time is set to 5 ms. (C, G) Probability histogram of photon counts per frame. (D, H) Probability histogram of ON periods. (E, I) Probability histogram of OFF periods. (F, J) Probability histogram of number of ON periods, i.e., ON events.

Experimentally measured fluorescence time traces at short interfluorophore distances did not show largely increased intensities compared to larger distances (Fig 2 a – e and Fig 1 e 1st plot of Helmerich et al.[10]). In contrast, our simulations indicate that coinciding ON states of four fluorophores produce 4-fold intensities (Fig 4E, M). Incorporating the energy transfer with the radical anion as acceptor combined with an increased formation rate solves this issue, leading to only slightly larger fluorescence intensities at short distances (Fig TC, Fig U).

Additionally, experiments showed shorter OFF periods at shorter interfluorophore distances (Fig 2 e 3rd plot of Helmerich et al.[10]). At first, shortening OFF periods via energy transfer mediated rescue of OFF states may seem intuitive, however energy transfer is only possible with an ON state as donor and hence ongoing fluorescence signal. Therefore, the more immediate consequence is the shortening of OFF state durations of individual fluorophores, making the link between shorter OFF periods and shorter inter-fluorophore distances less straightforward. Indeed, our simulations do not confirm this observation (Fig 4G), where the distribution of OFF periods even shifts to longer durations. The simulation result is due to most

collected OFF periods coming from the time where all fluorophores except one have photobleached, i.e., the distribution closely resembles the one of a single fluorophore.

Therefore, there can either be a relative enrichment of an already existing short OFF state that has a likelihood high enough to counter the increase in large OFF periods due to early photobleaching. Or it can be energy transfer-mediated such as (1) an acceptor that gets excited and has an increased probability of transitioning to a non-emitting state with significant lifetime or (2) efficient and consistent $S_1$-quenching. Incorporation of the energy transfer to R and an increased R formation rate combines two of those concepts and indeed increases the relative population of short OFF periods at short interfluorophore distances (Fig TE).

Note that OFF and ON periods are susceptible to many photophysical, imaging- and analysis-related parameters. For example, the result is very different if instead of 1 ms frame integration time, 5 ms is used (Fig TI), where the temporal resolution is not enough for the $10^{-4}$ – $10^{-3}$ s living radical anion. A larger lifetime of the radical anion (as reported by Srambickal et al.[5], depending on the experimental conditions) on the other hand would lead to OFF periods even at lower temporal resolution.

The adjusted photophysical model, including the additional energy transfer to R and the increased R formation rate, still produces the distance-dependent shapes of PFA (Fig TA, TB), confirming that the adjustment is compatible with our other findings.

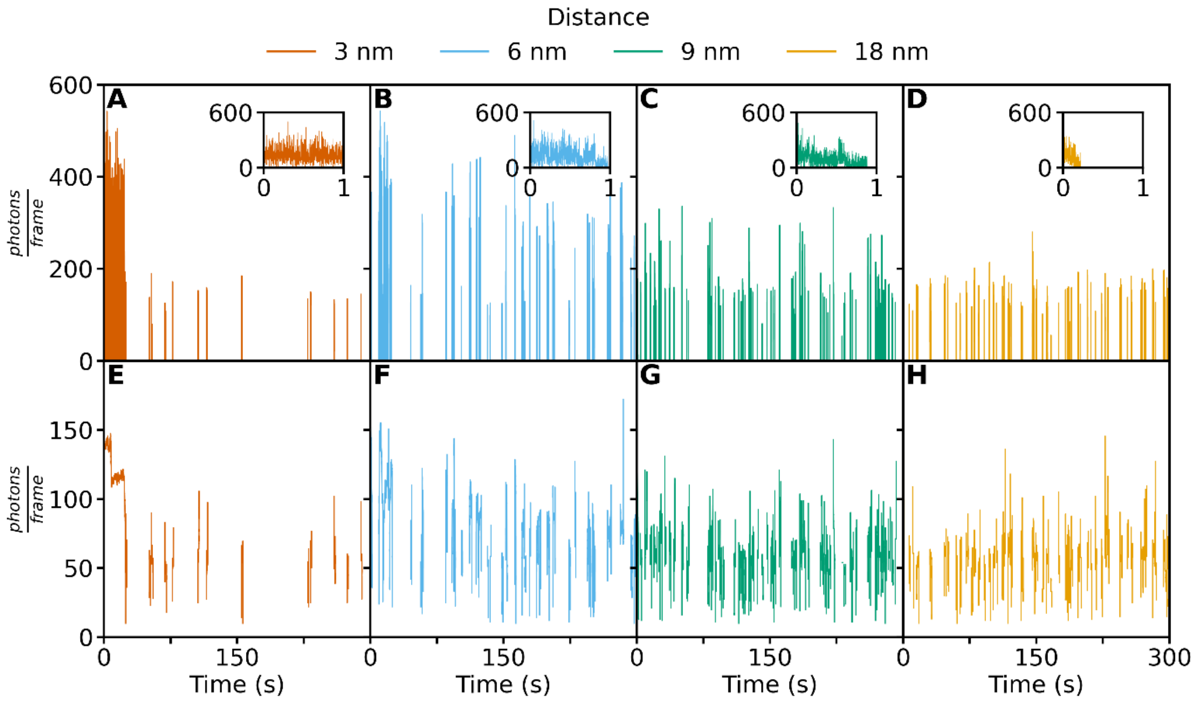


**Fig U. Fluorescence time traces of 4-fluorophore systems exhibiting OET and energy transfer to R.** Adjustments to photophysical model: $k_{PET_{TR}}$ × 100, energy transfer to R ($k_{3nm} = 10^9 s^{-1}$). (A – D) Standard fluorescence time traces. (E – H) Fluorescence time traces using a rolling average of 1 s.

# 8 PFA analytical expression

We provide an analytical expression to describe PFA given that some assumptions hold. The PFA model is not intended to be used for fitting experimental data, as the number of parameters grows with the number of underlying fluorophores, introducing a high degree of freedom. This is especially true, if knowledge about parameter value ranges is limited. However, given that the parameters are known (see SI section 8.4), it does outperform simpler expressions (Fig WE – WH).

Additionally, if a n-fluorophore system decays into unique configurations, that is, there is only a single configuration for the (n-k)-fluorophore system where $0 < k < n$, this configuration's PFA can be well approximated using a subset of parameters of the n-fluorophore system (Fig X). In other words, the parameter set of a 4-flurophore system arranged like a tetrahedron of side length h fully contains the parameters needed to describe the 3-fluorophore system arranged like an equilateral triangle of side length h, which in turn contains the parameters needed to describe the 2-fluorophore system of distance h. In this case, simulations of the n-fluorophore system are sufficient to predict the PFA of fluorophore systems of smaller number of fluorophores.

Another use case is to construct a surrogate model of an n-fluorophore system for parameters as a function of the distance, e.g., by simulating and fitting parameters for distances [3, 4, ..., 9 nm] to interpolate parameters and hence the shape of PFA of e.g., 4.2 nm. Those PFA predictions can be compared with experimental PFA to either confirm a photophysical model or a fluorophore arrangement (number of fluorophores, distances). A surrogate model could also be established for other variables, e.g., a varying photophysical rate constant. This may also allow for deducing parameter variation given the varying experimental conditions, creating a range of PFA shapes representing uncertain photophysical or fluorophore arrangement characteristics.

When it comes to sampling from the PFA model, it must be considered that the PFA model describes data on the ensemble level. The more individual fluorophore systems make up the data, the better it can be described by the PFA model. Therefore, the PFA model cannot be used to directly sample photon arrival times that represent a single fluorophore system. Furthermore, experimental PFAs may only contain photons of an intermediate or low amount of clusters, which may not be as accurately described by the PFA model.

## 8.1 Assumptions

**(1) The data represents ensembles of fluorophore systems.**

In a single system (i.e. a photophysical system that consists of one or more fluorophores for an extended time) under dSTORM conditions, photons are emitted in bursts (ON periods) separated by long periods of time without any emission taking place (OFF periods). Such correlations in time are getting less significant when averaged over an increasing number of systems, as done in ensemble fluorescence observation. Additionally, in a single system, each fluorophore can either be photobleached during bias or non-bias (i.e., it is non-ergodic, see SI section 8.2), whereas the PFA model describes a mixture of those. Finally, photon arrival times and global bleaching times follow the same distribution (see below), only that the absolute number of sampled photon arrival times is much larger than the global bleaching times (which are limited to $n$ in an $n$-fluorophore system).

**(2) Photon arrival times, photobleaching times, localization times and ON state times are distributed identically**

While the PFA model can be applied without this assumption, the assumption is required for the PFA model to hold uniformly across all entities assumed to be identically distributed. It requires assumption (1) to hold. Due to their different sample size, the assumption can also be phrased as: photobleaching times are a subset of ON state times, which in turn are a subset of localization times, which themselves are a subset of photon arrival times.

The PFAs shown in this study are based on photon arrival times. They are experimentally available and inherently reflect increased photon rates, whereas localization times may mask such changes due to grouping. Hence, if the number of photons per frame is increased, PFA using photon arrival times can be more sensitive compared to using localization times instead (see Fig 4 and Fig V). However, if the number of photons per frame does not change significantly, localization times and photon arrival times are interchangeable.

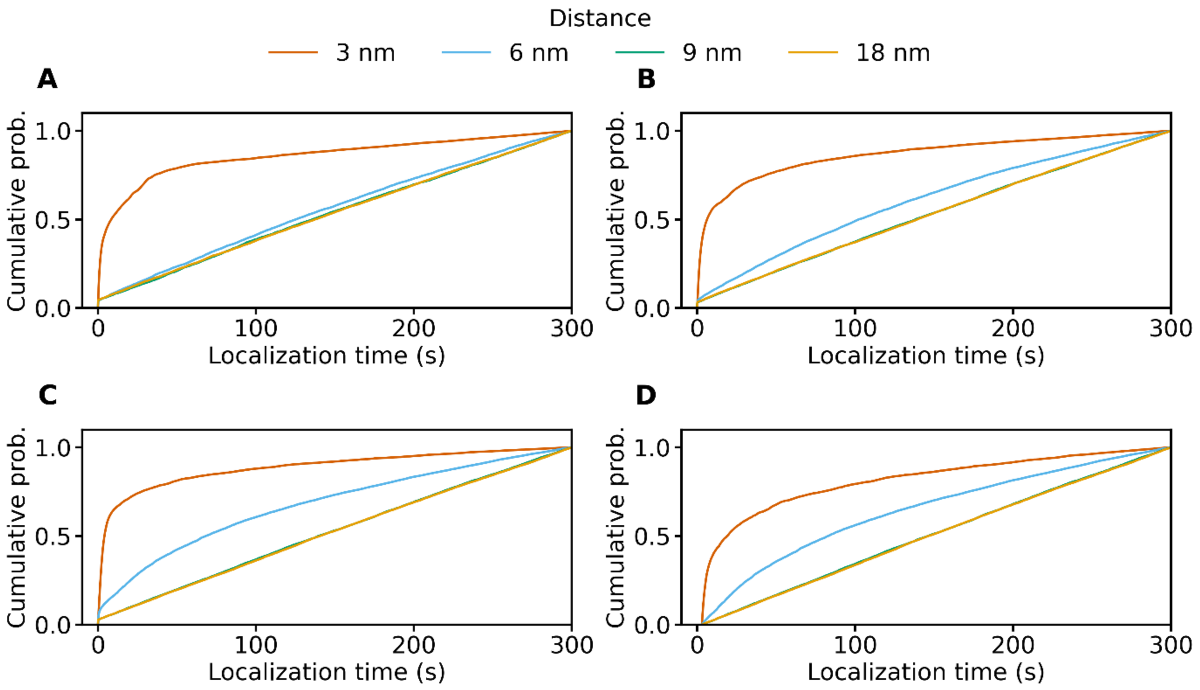


**Fig V. PFA of multi-fluorophore systems undergoing OET using localization times instead of photon arrival times.** This figure is based on the same data as Fig 4. (A) Two fluorophores, (B) three fluorophores, (C, D) four fluorophores. In case of (D), the data is truncated such that only times ≥ 3 s are included.

If the ratio of probability of photobleaching per excitation $P_{BLE}$ to the probability of fluorescence per excitation $P_{FLU}$ is constant throughout a measurement, photon arrival times and photobleaching times are identically distributed. For example, at the beginning of a measurement all fluorophores are intact, and energy transfers are more likely. If these energy transfers compete with fluorescence the same way they compete with photobleaching (per $S_1$), then our assumption is met. This is the case for all energy transfers that are implemented in our standard photophysical model (see Fig WA – WD, WF). However, energy transfer mediated photoionization and subsequent photobleaching (Fig J) increases $P_{BLE}$ and decreases $P_{FLU}$, and RISC following STA (Fig K) decreases $P_{BLE}$ more than it decreases $P_{FLU}$. Note that these considerations concern systems of identical fluorophores, where fluorescence is measured equally for both donor and acceptor.

Regarding the ON states, note that they are not equal to ON periods. Here, the same argument as in the case of photobleaching applies: if the ratio of probability of entering the OFF state per excitation $P_{OFF}$ to the probability of fluorescence per excitation $P_{FLU}$ is constant throughout the measurement, photon arrival times and ON state times are identically distributed. This assumes that the exact time points within an ON state are negligible – this is not necessarily

the case if the OFF state lifetime is reduced due to OET ($\tau_{ON} \ll \tau_{OFF}$ no longer applies). In other words, the expected number of photons per ON state needs to be constant. This is the case for our standard simulation routine. Again, RISC following STA deviates from this assumption as it decreases $P_{OFF}$ more than it decreases $P_{FLU}$.

**(3) The time difference of photobleaching events of the $n$th to the $n-1$th fluorophore is a mixture distribution of two exponential distributions.**

*Single-fluorophore system*

The two components arise due to a bias towards the beginning of a measurement, where all fluorophores are in the ON state. Hence, in a single-fluorophore system, the bias concerns photobleaching within the first ON state. The waiting time $\tau$ for photobleaching is a series of steps in the Markov chain. The number of trials $K$, where it is decided whether to photobleach or not, is geometrically distributed. As the success rate $p$ gets lower, $K$ increases and dominates $\tau = \sum_i^K t_i$, such that the distribution of $t$ (the time between trials) becomes insignificant and the distribution of $\tau$ becomes exponential with rate $\frac{p}{\mu_t}$. Additionally, projected onto photon arrival times, the time resolution is limited by integration bins such that potential skewing is unresolved.

The non-biased part can be described as multiple OFF state durations interrupted by short ON state durations. The durations of the latter can be neglected. The number of OFF state durations $K$ before photobleaching is again geometrically distributed. Since the OFF state is believed to be comprised of at most two different photophysical states that live on a similar time scale, we chose a single exponential to approximate the OFF state durations $T$. Hence, we are looking for the compound distribution of $S = \sum_i^K T_i$, which resembles the non-biased photobleaching times. This is equivalent to the Erlang distribution (which results from convolution of identical exponential distributions) $Erlang(k, \lambda)$, where shape parameter $k$ is geometrically distributed as $Geom_{shifted}(p)$. It can be shown that this is also equivalent to the exponential distribution with rate $\lambda p$.

Note that the geometric distribution of the number of OFF states before photobleaching holds even if multiple photobleaching pathways (photophysical or photochemical) are present, as long as a constant probability of photobleaching per ON state exists. Our photophysical model comprises only one photobleaching pathway.

*Multi-fluorophore system*

Without OET, the scenario is the same as above – a single cluster or simulated system with $n$ fluorophores then represents $n$ clusters or simulated systems of one fluorophore. In the following, multi-fluorophore systems exhibiting OET is discussed.

In multi-fluorophore systems, the bias in the beginning of a measurement can consist of multiple ON states per fluorophore as the stochastically occurring OFF states can be rescued by ON states of other fluorophores. The OFF states that get rescued are short lived such that $\tau = K * t$ can again be assumed to be dominated by the geometrically distributed $K$. If they are long lived it indicates that other fluorophores also entered the OFF state marking the onset of the non-bias.

During non-bias, each ON state has a nonzero probability to convert other potential OFF states to the ON state. Hence, the distribution of OFF state durations $T$ deviates from the exponential distribution. The degree of deviation depends on the number of fluorophores and the probability of rescue per ON state. The number of OFF states before photobleaching $K$ is again geometrically distributed (which follows from assumption (2)).

To be more precise, $T$ is a mixture distribution of different exponential distributions. There is the possibility for OFF states to live on the timescale of ON states, for example if a fluorophore's OFF state is rescued multiple times within an ON period (by any other fluorophore currently in the ON state), however they are short enough to be treated as 0 (they still need to be included as they represent one of $K$). Other than that, there are $n$ different exponential components with rates $\lambda \in \{\frac{m}{D} | m \in \{1,2,\dots,n\}\}$ in a $n$-fluorophore system, where $D$ is the OFF state lifetime in a single-fluorophore system. The compound distribution $S = \sum_i^K T_i$ is a mixture of different hypoexponential distributions.

Hence, the assumption of a monoexponential distribution is an approximation and its goodness of fit depends on the number of fluorophores and inter-fluorophore distance. For example, if OET is very efficient most fluorophores photobleach within the bias time interval. If some do not photobleach, there is a very high chance of establishing another positive feedback loop when one fluorophore reenters the ON state. If the efficiency of OET is low, it can be approximated with the single-fluorophore scenario. Therefore, it is an approximation mainly for scenarios where efficiencies are of a moderate level. Note that efficiency depends on the number of fluorophores left, so if a moderate efficiency applies for a $n$-fluorophore system, it is likely that the efficiency is high for $n + 1$ and low for $n - 1$. Also, each combination is assigned to its own global photobleaching rate. Finally, if only one fluorophore is left, the single-fluorophore system considerations take effect.

Note that, additionally to the altered OFF state lifetimes due to OET, a multi-fluorophore system can also exhibit altered ON state lifetimes (e.g., energy transfer induced *cis* to *trans* conversion). A single global photobleaching rate associated with the corresponding number of fluorophores can represent both altered OFF and ON state lifetimes.

**(4) Photobleaching of the nth fluorophore during bias can only happen if the n-1th fluorophore photobleached during bias**

The bias component of the two exponential mixture distribution describing the time differences of photobleaching events of the nth to the n-1th fluorophore is assumed to fully arise in the beginning of a measurement. This is an approximation, since non-biased photobleaching events of the n-1th fluorophore can indicate other fluorophores to be in their ON state following OET. However, the beginning of a measurement guarantees all fluorophores to be in their ON state, whereas non-biased photobleaching may only be accompanied by a fraction of other fluorophores in their ON state, if at all.

**(5) No system-to-system differences**

Each system is assumed to have the same parameters, that is the same number of fluorophores, the same set of transitions and the same transition rates.

**(6) There is only one configuration for each fluorophore that has photobleached**

The symmetry of the fluorophore system should be close to a Euclidean simplex, meaning that if fluorophores photobleach, each time the system can adopt only one possible configuration. In other words, the 4-fluorophore system should be a tetrahedron. A deviation is encountered in our standard 4-fluorophore system (a square), since whenever two fluorophores have photobleached, two different configurations are possible. One workaround is the introduction of a three-exponential mixture distribution (see SI section 8.2) instead of a two-exponential mixture distribution as discussed in assumption (3).

## 8.2 Derivation

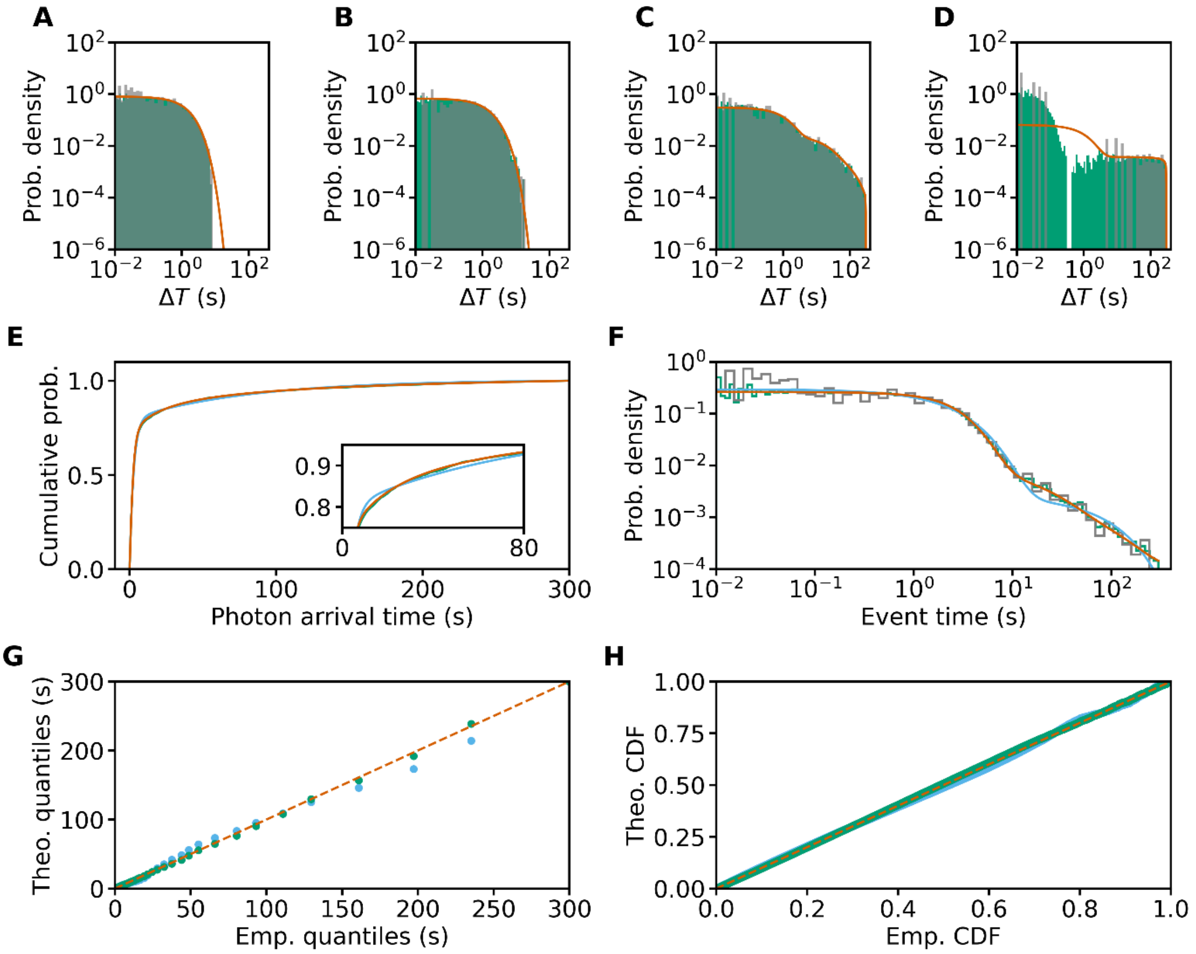


**Fig W. Modeling of PFAs.** This figure is based on the same data as Fig 4C 3 nm. (A – D) ePDF of event times of photons (green) and photobleaching (grey) up until the 1st (A), 2nd (B), 3rd (C) and 4th (D) photobleaching event (or until the upper truncation limit – 300 s) starting from the previous photobleaching event. The fits shown in red are mixtures of 2 (A, B, D) and 3 (C) exponential distributions marginalized over the distribution of the variable truncation limits. (E – H) Evaluation of goodness of fit of the PFA model. The PFA model combines the parameters of the mixture distributions of (A – D) to describe the photon arrival times or photobleaching times on a global timescale. For comparison, a 2-exponential mixture distribution is shown in blue. (E) eCDF (green) and CDF (orange). (F) ePDF of photon arrival times (green), ePDF of photobleaching times (grey) and PDF (orange). (G) QQ-Plot with dotted reference line. (H) PP-Plot with dotted reference line.

Photoswitching fingerprints in experimental data are empirical cumulative distribution functions (eCDF) of localization times over the full measurement duration. Here we use photon arrival times as they are independent of variations in photon count per localization (see SI section 8.1). We now aim at finding an analytical model that fully describes the underlying probability distribution and thus the eCDF.

It would be straightforward to use global photon arrival rates as parameters that determine the probability distribution for photon arrival times. However, given that energy transfers in multi-fluorophore systems are responsible for increased global photon arrival rates, photobleaching changes these rates throughout a measurement. Additionally, without photobleaching, each eCDF would be linear on a large timescale.

We therefore suggest using global bleaching rates $\lambda_{gB}$ instead as each photobleaching event is associated with a change of global photon arrival rate. Again 'global' is used to distinguish the global bleaching rate from rate constants for selected transitions (e.g., $k_{T1\to B}$). For a system with $n$ number of fluorophores, we have $n$ different $\lambda_{gB}$, where

$$\lambda_{gB|1} > \lambda_{gB|2} > \cdots > \lambda_{gB|n}. \tag{1}$$

Simulations indeed show that both photon arrival times and photobleaching event times result in the same probability distribution (Fig WF).

To derive the analytical expression for the bleaching time probability distribution, we must consider that each measurement starts in a non-equilibrium with all fluorophores being in their ground state, and thus in the ON state. The onset of illumination introduces an equilibration period (we call it bias) making photon arrival more likely until OFF states are populated. This is particularly important for multi-fluorophore systems with small inter-fluorophore distances where rescuing the OFF state requires other fluorophores to be in the ON state. Given that photobleaching can already happen during the equilibration (bias) period, we need to introduce a bias ($b$) and non-bias ($nb$) version of $\lambda_{gB}$ with

$$\lambda_{gB|b|i} > \lambda_{gB|nb|i}, \forall i \in [1, \dots, n]. \tag{2}$$

Note that most experimental procedures require an alignment step that exposes fluorophores to light, thus mitigating the bias.

We can assume most individual global photobleaching processes to be exponential (see SI section 8.1). Therefore, the distribution of

$$\Delta t_i = \begin{cases} t_i, \ if\ i = 1 \\ t_i - t_{i-1}, \ if\ i \geq 2 \end{cases} \tag{3}$$

where $t_i$ is the time of fluorophore $i$ being photobleached, is a mixture of two exponential distributions with parameters $\lambda_{gB|b|i}, \lambda_{gB|nb|i}, p_i$ (Fig WA – WD). Hence, the lifetime of a fluorophore, potentially starting from a previous photobleaching event, is $\frac{1}{\lambda_{gB}}$. The distribution of $t_i$ is then a mixture distribution of all valid convolved tuples, e.g.,

$$t_2 \sim p_2 * Conv\left(Exp(\lambda_{gB|b|1}), Exp(\lambda_{gB|b|2})\right) + p_1 * (1 - p_2) * Conv\left(Exp(\lambda_{gB|b|1}), Exp(\lambda_{gB|nb|2})\right) + (1 - p_1) * (1 - p_2) * Conv\left(Exp(\lambda_{gB|nb|1}), Exp(\lambda_{gB|nb|2})\right) \tag{4}$$

The tuple $\left(Exp(\lambda_{gB|nb|1}), Exp(\lambda_{gB|b|2})\right)$ is not valid, because if the first fluorophore has not photobleached during bias, no other fluorophore can (see assumption (4), SI section 8.1). Another consequence is the constraint

$$p_i > p_{i+1} > \cdots > p_n. \tag{5}$$

The CDF of $t_i$ is then

$$F_i(t) = \sum_{\substack{(j_1,\dots,j_i) \in \{b,nb\}^i \\ s.t.\ j_k = nb\ if\ j_{k-1} = nb}} \left(\prod_{h=1}^{i} w_h\right) F_{Hypo}(\lambda_{gB|j_1|1}, \dots, \lambda_{gB|j_i|i}), \tag{6}$$

Where $w_h \begin{cases} p_h\ if\ h = i, j_i = b \\ 1\ if\ h \neq i, j_i = b \\ p_h\ if\ j_h = b, j_i = nb \\ 1 - p_h\ if\ j_h = nb, j_{h-1} = b, j_i = nb \\ 1\ if\ h \neq i, j_h = nb, j_{h-1} = nb \end{cases}$ and $F_{Hypo}(t, \lambda_{gB|j_1|1}, \dots, \lambda_{gB|j_i|i})$ is the CDF of the hypoexponential distribution and equal to

$$\left(1-\sum_{l=1}^{i}\frac{\left(\prod_{g\neq l}^{i}\lambda_{gB|j_g|g}\right)e^{-\lambda_{gB|j_l|l}*t}}{\prod_{g\neq l}^{i}\left(\lambda_{gB|j_g|g}-\lambda_{gB|j_l|l}\right)}\right). \quad (7)$$

Each fluorophore eventually photobleaches, therefore all $n$ components mix with the same weight and the total CDF becomes

$$F(t,n)=\sum_{i=1}^{n}\frac{1}{n}F_i(t). \quad (8)$$

Finally, a measurement often misses the very beginning of an experiment such that $t_1>0$ and both simulation and experiment end at a certain point in time $t_2<\infty$. This can be accounted for using truncation:

$$F(t|t_1<T\leq t_2)=\frac{F(t)-F(t_1)}{F(t_2)-F(t_1)}. \quad (9)$$

For some $q\in\{1,\dots,n\}$ of an $n$-component system, the non-biased $\Delta t_{i=q}$ may be much better described using a mixture of two exponential distributions instead of a single exponential distribution. $\Delta t_{i=q}$ is then a mixture of three exponential distributions with parameters $\lambda_{gB|b|q},\lambda_{gB|nb_1|q},\lambda_{gB|nb_2|q},p_{q,b},p_{q,nb_1},p_{q,nb_2}$. The CDF of all $t_i$ is then

$$F_i(t)=\sum_{\substack{(j_1,\dots,j_i)\in\prod_{k=1}^{i}S_k\\ s.t.\ j_k=nb\ if\ j_{k-1}=nb}}\left(\prod_{h=1}^{i}w_h\right)F_{Hypo}(\lambda_{gB|j_1|1},\dots,\lambda_{gB|j_i|i})$$

With $S_k=\begin{cases}\{b,nb_1,nb_2\},k=q\\ \{b,nb_0\},k\neq q\end{cases}$, $w_h\begin{cases}p_h\ if\ h=i,j_i=b\\ 1\ if\ h\neq i,j_i=b\\ p_h\ if\ j_h=b,j_i=nb\\ 1-p_h\ if\ j_h=nb_0,j_{h-1}\in\{b,None\},j_i=nb\\ p_{q,j_h} if\ j_h\in\{nb_1,nb_2\},j_{h-1}\in\{b,None\},j_i=nb\\ 1-p_h\ if\ h=i,j_h=nb_0\\ p_{q,j_h}\ if\ h=i,j_h\in\{nb_1,nb_2\}\\ 1\ if\ h\neq i,j_h=nb_0,j_{h-1}=nb,j_i=nb\\ \frac{p_{q,j_h}}{p_{q,nb_1}+p_{q,nb_2}}\ if\ h\neq i,j_h\in\{nb_1,nb_2\},j_{h-1}=nb,j_i=nb\end{cases}$

This may be the case if the fluorophore system can decay into multiple different configurations.

# 8.3 Properties

PDF: $f(t,z,n)=\frac{d^z f(t,n)}{dt^z}=\sum_{i=1}^{n}\frac{1}{n}f_i(t,z)$ for $t\in[0,\infty)$, with $f_i(t,z)=$

$$\sum_{\substack{(j_1,\dots,j_i)\epsilon\{b,nb\}^i\\ s.t.\ j_k=nb\ if\ j_{k-1}=nb}}\left(\prod_{h=1}^{i}w_h\begin{cases}1\ if\ j_{h-1}=nb\\ p_h\ if\ j_h=b\\ 1-p_h\ if\ j_h=nb\end{cases}\right)f_{Hypo}(t,z,\lambda_{gB|j_1|1},\dots,\lambda_{gB|j_i|i})$$

where $f_{Hypo}\left(t,z,\lambda_{gB|j_1|1},\dots,\lambda_{gB|j_i|i}\right)=\sum_{l=1}^{i}\frac{\left(\prod_{l}^{i}\lambda_{gB|j_l|l}\right)g(t,z,\lambda_{gB|j_l|l})}{\prod_{g\neq l}^{i}\left(\lambda_{gB|j_g|g}-\lambda_{gB|j_l|l}\right)}$, with $g\left(t,z,\lambda_{gB|j_l|l}\right)=\frac{d^z e^{-\lambda_{gB|j_l|l}*t}}{dt^z}$

CDF: $F(t,n)=\sum_{i=1}^{n}\frac{1}{n}F_i(t)$, with $F_i(t)=$

$$\sum_{\substack{(j_1,\dots,j_i)\epsilon\{b,nb\}^i\\ s.t.\ j_k=nb\ if\ j_{k-1}=nb}}\left(\prod_{h=1}^{i}w_h\begin{cases}1\ if\ j_{h-1}=nb\\ p_h\ if\ j_h=b\\ 1-p_h\ if\ j_h=nb\end{cases}\right)F_{Hypo}(\lambda_{gB|j_1|1},\dots,\lambda_{gB|j_i|i})$$

where $F_{Hypo}\left(t, \lambda_{gB|j_1|1}, \ldots, \lambda_{gB|j_i|i}\right) = (1 - \sum_{l=1}^{i} \frac{(\prod_{g\neq l}^{i} \lambda_{gB|j_g|g}) e^{-\lambda_{gB|j_l|l} * t}}{\prod_{g\neq l}^{i}\left(\lambda_{gB|j_g|g} - \lambda_{gB|j_l|l}\right)})$

**Parameter constraints**

$\lambda_{gB|b|i} > \lambda_{gB|nb|i}, \forall i \in [n]$

$\lambda_{gB|b|i} > \lambda_{gB|b|i+1}, \forall i \in [n]$

$\lambda_{gB|nb|i} > \lambda_{gB|nb|i+1}, \forall i \in [n]$

$p_i > p_{i+1}, \forall i \in [n]$

Numerical experiments suggest that $f(t, z, n)$ is unimodal with $x^* = 0$, convex and bounded, although we do not provide a formal proof. Additionally, the distribution is neither log-concave nor $\frac{1}{\sqrt{}}$-concave. The quantile function has no closed form.

## 8.4 Fitting procedure and parameters

The parameters were determined by subdividing the fitting process into several components, fitting individual sets of Δ-photon arrival times each with a 2- or 3-exponential mixture distribution. A set of Δ-photon arrival times are times of photon arrival collected between the previous photobleaching event up until the next photobleaching event (or until the end of the measurement), i.e., a 4-fluorophore system has 4 sets of Δ-photon arrival times. Therefore, the upper truncation limits are variable within a single set if a set consists of multiple simulation runs (i.e., clusters), so the mixture distributions were marginalized over the distribution of the truncation limits.

We used the SciPy implementation for the differential evolution algorithm with a population size of 200 and up to 1,000 iterations. We set the bounds for all $\lambda_{gB|i}$ to [$10^{-9}$, 5] and applied the constraints given in Eq. 1, 2 and 5.

The PFA model then combines those parameters to describe the photon arrival times globally. This kind of fitting procedure is only available for simulated data, as it requires knowledge of photobleaching time points.

Table E shows some example parameters resulting from fitting two (2F), three (3F), and four fluorophores (4F) at 3 nm distance. As the 4F decays into a 3F system, the parameters yielded by the 3F can be viewed as a subset of the 4F. However, this is an approximation since the simulation of only 3F starts in an all-ON state whereas the 3F that are left from the 4F are not necessarily all-ON when the photobleaching event happens. The 2F system is less similar to the corresponding subset of 4F and 3F because the 2F they decay into have some variability in their configuration (3 nm and 4.24 nm), whereas the 2F is always at 3 nm.

**Table E. PFA Model parameters of simulated multi-fluorophore systems at 3 nm distance.**

| $p_1$ | $\lambda_{gB|b|1}$ | $\lambda_{gB|nb|1}$ | $p_2$ | $\lambda_{gB|b|2}$ | $\lambda_{gB|nb|2}$ | $p_{3|b}$ | $p_{3|nb_1}$ | $\lambda_{gB|b|3}$ | $\lambda_{gB|nb_1|3}$ | $\lambda_{gB|nb_2|3}$ | $p_4$ | $\lambda_{gB|b|4}$ | $\lambda_{gB|nb|4}$ |
|---|---|---|---|---|---|---|---|---|---|---|---|---|---|
| / | / | / | / | / | / | 2.3e-2 | 4.1e-1 | 2.5e0 | 8.1e-1 | 5.2e-2 | 9.8e-3 | 2.1e0 | 3.5e-4 |
| / | / | / | 4.4e-1 | 9.7e-1 | 4.1e-1 | 2.4e-1 | 4.8e-1 | 9.6e-1 | 4.7e-2 | 1.0e-2 | 7.9e-3 | 6.4e-1 | 3.0e-4 |
| 3.7e-1 | 9.5e-1 | 7.3e-1 | 3.7e-1 | 9.5e-1 | 5.2e-1 | 3e-1 | 3.3e-1 | 9.5e-1 | 5.7e-2 | 1.3e-2 | 6.7e-3 | 9.4e-1 | 3.8e-4 |

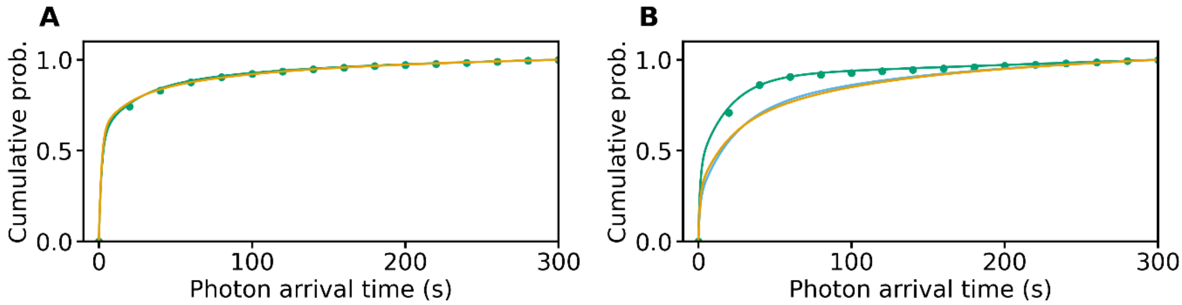


**Fig X. Fitting PFA of n fluorophores at 3 nm distance with parameters yielded by fitting PFAs of m > n fluorophores.** The eCDF of photon arrival times (markers) fit with different parameter sets (solid lines). (A) Simulation of three fluorophores (green markers, equivalent to Fig 4B 3 nm) fit with parameters of 3F fit (green line) and the corresponding subset of parameters of 4F fit (orange line, fit of Fig 4C 3 nm). (B) Simulation of two fluorophores (green markers, equivalent to Fig 4A 3 nm) fit with parameters of 2F fit (green line) and the corresponding subset of parameters of 3F fit (blue line, fit of Fig 4B 3 nm) and 4F fit (orange line, fit of Fig 4C 3 nm).

# 9 References

1. Xu, J. X., Hu, J. & Zhang, D. Quantification of Material Fluorescence and Light Scattering Cross Sections Using Ratiometric Bandwidth-Varied Polarized Resonance Synchronous Spectroscopy. *Analytical chemistry* **90,** 7406–7414; 10.1021/acs.analchem.8b00847 (2018).
2. Widengren, J. & Schwille, P. Characterization of Photoinduced Isomerization and Back-Isomerization of the Cyanine Dye Cy5 by Fluorescence Correlation Spectroscopy. *J. Phys. Chem. A* **104,** 6416–6428; 10.1021/jp000059s (2000).
3. Gidi, Y. *et al.* Unifying Mechanism for Thiol-Induced Photoswitching and Photostability of Cyanine Dyes. *Journal of the American Chemical Society* **142,** 12681–12689; 10.1021/jacs.0c03786 (2020).
4. Hübner, C. G., Renn, A., Renge, I. & Wild, U. P. Direct observation of the triplet lifetime quenching of single dye molecules by molecular oxygen. *The Journal of chemical physics* **115,** 9619–9622; 10.1063/1.1421382 (2001).
5. Srambickal, C. V. *et al. Near-infrared MINFLUX imaging enabled by suppression of fluorophore blinking* (2024).
6. Wu, P. & Brand, L. Resonance energy transfer: methods and applications. *Analytical biochemistry* **218,** 1–13; 10.1006/abio.1994.1134 (1994).
7. Hofkens, J. *et al.* Revealing competitive Forster-type resonance energy-transfer pathways in single bichromophoric molecules. *Proceedings of the National Academy of Sciences of the United States of America* **100,** 13146–13151; 10.1073/pnas.2235805100 (2003).
8. Ploetz, E. *et al.* A new twist on PIFE: photoisomerisation-related fluorescence enhancement. *Methods and applications in fluorescence* **12**; 10.1088/2050-6120/acfb58 (2023).
9. Mujumdar, R. B., Ernst, L. A., Mujumdar, S. R., Lewis, C. J. & Waggoner, A. S. Cyanine dye labeling reagents: sulfoindocyanine succinimidyl esters. *Bioconjugate chemistry* **4,** 105–111; 10.1021/bc00020a001 (1993).
10. Helmerich, D. A. *et al.* Photoswitching fingerprint analysis bypasses the 10-nm resolution barrier. *Nature methods* **19,** 986–994; 10.1038/s41592-022-01548-6 (2022).
11. Gillespie, D. T. Stochastic simulation of chemical kinetics. *Annual review of physical chemistry* **58,** 35–55; 10.1146/annurev.physchem.58.032806.104637 (2007).
12. Pati, A. K. *et al.* Recovering true FRET efficiencies from smFRET investigations requires triplet state mitigation. *Nature methods* **21,** 1222–1230; 10.1038/s41592-024-02293-8 (2024).
13. Basché, T., Moerner, W. E., Orrit, M. & Talon, H. Photon antibunching in the fluorescence of a single dye molecule trapped in a solid. *Physical review letters* **69,** 1516–1519; 10.1103/PhysRevLett.69.1516 (1992).
14. Tinnefeld, P., Müller, C. & Sauer, M. Time-varying photon probability distribution of individual molecules at room temperature. *Chemical Physics Letters* **345,** 252–258; 10.1016/S0009-2614(01)00883-1 (2001).
15. Tinnefeld, P. *et al.* Antibunching in the emission of a single tetrachromophoric dendritic system. *Journal of the American Chemical Society* **124,** 14310–14311; 10.1021/ja027343c (2002).

16. Hübner, C. G. *et al.* Photon antibunching and collective effects in the fluorescence of single bichromophoric molecules. *Physical review letters* **91,** 93903; 10.1103/PhysRevLett.91.093903 (2003).

17. Ma, H. *et al.* New Cy5 photosensitizers for cancer phototherapy: a low singlet-triplet gap provides high quantum yield of singlet oxygen. *Chemical science* **12,** 13809–13816; 10.1039/d1sc04570a (2021).
